# Evaluation of an Optimal Epidemiological Typing Scheme for Legionella pneumophila with Whole-Genome Sequence Data Using Validation Guidelines

**DOI:** 10.1128/JCM.00432-16

**Published:** 2016-07-25

**Authors:** Sophia David, Massimo Mentasti, Rediat Tewolde, Martin Aslett, Simon R. Harris, Baharak Afshar, Anthony Underwood, Norman K. Fry, Julian Parkhill, Timothy G. Harrison

**Affiliations:** a Wellcome Trust Sanger Institute, Wellcome Genome Campus, Hinxton, Cambridge, United Kingdom; b Public Health England, London, United Kingdom; c The European Programme for Public Health Microbiology Training, European Centre for Disease Prevention and Control, Stockholm, Sweden; Medical College of Wisconsin

## Abstract

Sequence-based typing (SBT), analogous to multilocus sequence typing (MLST), is the current “gold standard” typing method for investigation of legionellosis outbreaks caused by Legionella pneumophila. However, as common sequence types (STs) cause many infections, some investigations remain unresolved. In this study, various whole-genome sequencing (WGS)-based methods were evaluated according to published guidelines, including (i) a single nucleotide polymorphism (SNP)-based method, (ii) extended MLST using different numbers of genes, (iii) determination of gene presence or absence, and (iv) a kmer-based method. L. pneumophila serogroup 1 isolates (*n* = 106) from the standard “typing panel,” previously used by the European Society for Clinical Microbiology Study Group on Legionella Infections (ESGLI), were tested together with another 229 isolates. Over 98% of isolates were considered typeable using the SNP- and kmer-based methods. Percentages of isolates with complete extended MLST profiles ranged from 99.1% (50 genes) to 86.8% (1,455 genes), while only 41.5% produced a full profile with the gene presence/absence scheme. Replicates demonstrated that all methods offer 100% reproducibility. Indices of discrimination range from 0.972 (ribosomal MLST) to 0.999 (SNP based), and all values were higher than that achieved with SBT (0.940). Epidemiological concordance is generally inversely related to discriminatory power. We propose that an extended MLST scheme with ∼50 genes provides optimal epidemiological concordance while substantially improving the discrimination offered by SBT and can be used as part of a hierarchical typing scheme that should maintain backwards compatibility and increase discrimination where necessary. This analysis will be useful for the ESGLI to design a scheme that has the potential to become the new gold standard typing method for L. pneumophila.

## INTRODUCTION

Legionellosis is an infection that ranges from a mild respiratory illness (Pontiac fever) to a severe and potentially fatal pneumonia (Legionnaires' disease). It is caused by members of the genus Legionella, consisting of 59 species (http://www.bacterio.cict.fr/l/legionella.html) and 70 serogroups (sgs) (1). While many of these species and serogroups have been implicated in disease (2), over 90% of Legionnaires' disease cases are caused by Legionella pneumophila sg1 (3).

Humans are most commonly infected with L. pneumophila via inhalation of contaminated aerosols from an environmental source (4). Common sources include cooling towers (5–7), spa pools (8, 9), decorative fountains (10, 11), and hot- and cold-water systems of large buildings (12, 13). When one or more cases are recognized, rapidly establishing the source of infection is important in order to implement corrective measures and prevent further infection. Together with epidemiological information, the rapid microbiological characterization of clinical and epidemiologically linked environmental isolates is essential to this process.

Many methods have been used for the characterization or epidemiological “typing” of L. pneumophila, including pulsed-field gel electrophoresis (PFGE) (14–16), amplified fragment length polymorphism (AFLP) analysis (17), and monoclonal antibody (MAb) subgrouping (18), and many of these are still used today as part of a combinatory approach by some laboratories. However, the current “gold standard” method developed by the European Working Group for Legionella Infections (EWGLI), now the European Society for Clinical Microbiology Study Group on Legionella Infections (ESGLI), is sequence-based typing (SBT) (19–22), a scheme analogous to multilocus sequence typing (MLST). The scheme uses a combination of seven housekeeping and virulence genes, and over 2,000 sequence types (STs) have now been reported (http://bioinformatics.phe.org.uk/legionella/legionella_sbt/php/sbt_homepage.php). The major advantage of SBT over all previous methods has been the ease of exchanging data between laboratories, a significant aid in the investigation of the many cases associated with either domestic or international travel. However, since a large proportion of cases are caused by just a small number of common STs (e.g., ST1) (23–25), the method can lack discriminatory power and investigations may remain unresolved.

Whole-genome sequencing (WGS) is playing an increasingly important role in public health microbiology and has applications in both outbreak investigations and surveillance of pathogens (26, 27). The most significant advantage of WGS over previous and current typing techniques is the extremely high level of discrimination that can be achieved. Crucially, the emergence of next-generation sequencing (NGS) technologies in recent years has led to sharp decreases in both cost and turnaround time, making WGS a viable option in public health reference laboratories (28).

Several studies have now demonstrated the feasibility of using WGS as a tool for investigating local point source outbreaks of legionellosis (29–33). All but one of these studies have used a single nucleotide polymorphism (SNP)-based (also known as mapping-based) approach for analyzing WGS data, which involves mapping sequence reads to an appropriate reference genome and detecting SNPs between isolates of interest. The exception is a study that described the development and application of an extended version of an MLST/SBT scheme, utilizing 1,521 core genes, and compared isolates using the number of allele differences, rather than the number of SNPs (32). Both types of approach have demonstrated that outbreak isolates are highly similar, with one SNP-based study describing differences of <15 SNPs between clinical and environmental isolates from a point source outbreak (29). They have also demonstrated that outbreak isolates can usually be distinguished from isolates that are temporally and spatially unassociated with the outbreaks. Furthermore, the application of WGS to a cluster of Legionnaires' disease cases in Edinburgh, United Kingdom, in 2012 (in which no environmental source was found) demonstrated that the clinical isolates consisted of multiple subtypes, despite being of the same uncommon ST, ST191, as defined by SBT (34). The authors suggested that this may represent a more complex outbreak involving multiple sources and the evolution of outbreak isolates over a long period, a conclusion that would not have been drawn without the increased resolution of WGS analysis.

In addition to the extended MLST scheme developed by Moran-Gilad and colleagues (32), another scheme utilizing 1,896 genes has been created in a recent study and shown to provide high resolution in subtyping ST1 isolates (35). However, no studies have yet evaluated the workability of any method (extended MLST or otherwise) as a standardized and portable typing tool for L. pneumophila. The development of a new typing scheme poses a number of additional challenges. First, it should be possible to obtain results (i.e., a type) for all, or almost all, isolates. Second, the methodology must produce reproducible results when performed at different times or by different laboratories. Third, the extremely high discrimination offered by WGS allows an almost unlimited number of types to be defined. The methodology chosen must therefore ensure that an appropriate level of epidemiological concordance is maintained and the number of types remains within a practical and useful range. Furthermore, the typing designations should remain stable for each isolate and not change rapidly during laboratory storage and subculture. Finally, the results should be in a form that can be easily standardized, exchanged between laboratories, and stored in a central database, as with the current SBT scheme.

The aim of this study was to evaluate the use of WGS for the epidemiological typing of L. pneumophila. We have determined the typeability (*T*), reproducibility (*R*), epidemiological concordance (*E*), discriminatory power (*D*), and stability (*S*) of several WGS-based methods, including (i) a mapping/SNP-based method, (ii) extended MLST using various numbers of genes, (iii) gene presence or absence, and (iv) a kmer-based method. Using published guidelines (36), we have compared their performance to that of the current gold standard (SBT), used with and without additional MAb subgrouping, and finally proposed the most appropriate and convenient WGS-based typing methodology for future development.

## MATERIALS AND METHODS

### Bacterial isolates.

One hundred six clinical and environmental L. pneumophila sg1 isolates from 10 European countries, obtained from the ESGLI culture collection, served as our primary test population (see Table S1 in the supplemental material). These comprise an epidemiologically “unrelated” panel of 79 isolates and an epidemiologically “related” panel of 44 isolates, with 17 isolates in both panels. This collection of isolates, known as the typing panel, was established by the ESGLI for the purpose of evaluating new typing methods, and the criteria on which they were selected are described elsewhere (37). All isolates have been extensively characterized by previous typing methods (19, 37–39). One isolate (EUL 112) from panel 1 yielded a different ST from that recorded (both *in silico* and via traditional SBT) and was therefore replaced with a related environmental isolate (EUL 114), which produced the expected ST. Draft genomes for 48 of these isolates have been previously published (S. David, C. Rusniok, M. Mentasti, L. Gomez-Valero, S. R. Harris, P. Lechat, J. Lees, C. Ginevra, P. Glaser, L. Ma, C. Bouchier, A. Underwood, S. Jarraud, T. G. Harrison, J. Parkhill, and C. Buchrieser, submitted for publication), while 58 were newly sequenced for this study.

In addition to the typing panel, a further 229 clinical and environmental isolates were analyzed (see Table S2 in the supplemental material). These include 6 isolates belonging to serogroups other than sg1 (comprising three epidemiologically “related” sets), 28 isolates from three well-defined point-source outbreaks in the United Kingdom (BBC, Portland Place [1988], Barrow-in-Furness [2002], and Hereford [2003]), and a further 195 isolates from major disease-associated STs (ST1, -37, -42, -47, and -62). The last group includes both epidemiologically “unrelated” isolates and further sets of epidemiologically “related” isolates. Complete genomes for two of these isolates (Paris and Lorraine) have been previously published (40, 41) together with a further 199 draft genomes (29, 42) (David et al., submitted). Draft genomes for 28 of these isolates from the culture collection at Public Health England (PHE) are newly sequenced.

### Study design.

Each WGS-based typing method was evaluated in accordance with the guidelines produced by the European Society for Clinical Microbiology and Infectious Diseases (ESCMID) Study Group on Epidemiological Markers (ESGEM) (36) and as in previous studies (19, 20, 37–39). Typeability (*T*) is defined as the proportion of isolates that can be assigned to a type using a particular method. For each of the WGS-based methods, we determined a specific set of criteria that isolates must meet in order to be deemed typeable (see individual sections on the methods). Reproducibility (*R*) was calculated as the proportion of pairs of sequencing replicates that were assigned to the same type (or in which no differences were observed) using each method. Epidemiological concordance (*E*) was defined as the proportion of epidemiologically related sets of isolates assigned to the same type (or in which no differences were observed) by each method. The index of discrimination (*D*) of each method was calculated using Simpson's index of diversity, as first described by Hunter and Gaston (43). The stability (*S*) of each method was assessed by analysis of three sets comprising isolates sampled from the same patient. These include two isolates sampled 15 days apart, three isolates cultured either via direct plating, via amoebal coculture, or from a fecal sample, and three isolates picked from single colonies on the primary isolation plate.

### Culture and DNA extraction.

L. pneumophila isolates, stored at −80°C in either the culture collections of the ESGLI or the PHE National Legionella Reference Laboratory, were grown at 37°C on buffered charcoal-yeast extract (BCYE) agar for 48 to 72 h prior to DNA extraction. High-quality DNA was extracted using either the Wizard (Promega UK, Southampton, United Kingdom) or PurElute (VH Bio, Gateshead, United Kingdom) kit according to the manufacturer's instructions. DNA was eluted in 1× Tris-EDTA (TE), pH 8.0, and quantified using Qubit (Life Technologies Ltd., Paisley, United Kingdom).

### Whole-genome sequencing.

Isolates were sequenced by the core sequencing facilities at either the Wellcome Trust Sanger Institute (WTSI) or PHE. Paired-end libraries were constructed as described previously (44, 45), and sequencing was performed on all samples using the Illumina HiSeq platform and paired-end reads of 100 bases.

Four isolates (EUL 28, EUL 120, EUL 165, and H044120014, belonging to ST23, -42, -37, and -62, respectively) were also sequenced on the Pacific Biosciences (PacBio) RSII sequencer at the WTSI, in order to produce high-quality reference genomes for some of the major disease-associated STs. Between 1 and 2 μg of DNA per isolate was sheared using a 26G blunt-ended needle (Thermo Fisher, United Kingdom) and used in library preparation according to the manufacturer's protocol. The sequencing was performed using C2 chemistry with the P4 polymerase. The total number of mapped reads ranged from 45,460 to 100,833, and the mean N50 (the length of the shortest contig such that the sum of contigs of equal lengths or longer is at least 50% of the sum of all contig lengths) of the mapped reads ranged from 2.97 kb to 4.59 kb. The mean coverage of each genome ranged from 62.0× to 121.8×. Details for each isolate are provided in Table S3 in the supplemental material.

### *De novo* assembly.

*De novo* assemblies from Illumina sequence data were generated using an in-house pipeline at the WTSI. This uses Velvet Optimizer (http://bioinformatics.net.au/software.velvetoptimiser.shtml) to determine the optimal kmer size to use for the assembly, Velvet (46) for the initial assembly, iterations of SSPACE (47) to scaffold the contigs of the assembly, and 120 iterations of GapFiller (48) to close gaps of 1 or more uncalled bases (“Ns”). The mean number of contigs of all *de novo* assemblies was 39.9 (range, 12 to 140), the mean N50 value was 249,103 (range, 81,272 to 2,134,649), and the mean length was 3,476,414 bp (range, 3,229,839 to 3,710,927 bp). The ST was derived from the *de novo* assembly using an in-house script at the WTSI and compared with the previously designated ST as determined via traditional SBT, to help verify that no sample mix-ups had occurred *en route* to and during the sequencing procedures. Due to the presence of multiple copies of the *mompS* (SBT) gene that are occasionally nonidentical, it was not possible to determine the *mompS* allele number for some isolates *in silico*.

HGAP.3 (Pacific Biosciences) was used to perform the *de novo* assemblies from the PacBio sequence data. For each of the four isolates, the reads assembled into either one or two contigs, and one isolate (EUL 28) also possessed a single extrachromosomal plasmid (see Table S3). The assemblies consisting of one chromosomal contig were circularized using the overlap at either end, and the start was set at *dnaA*. The final genome was confirmed by the remapping of Illumina sequence data.

### Mapping/SNP-based analysis.

Due to the high diversity of L. pneumophila sequences, the mapping of all sequence reads to a single reference genome can result in large amounts of unmapped reads as well as a high number of false-positive SNP calls. Therefore, each pair of sequence reads was mapped to a closely related reference genome, as determined using KmerID (available from https://github.com/phe-bioinformatics/kmerid). This is a very rapid kmer-based program that compares raw sequence reads against a collection of predefined reference genomes. The reference genomes used included previously published genomes of L. pneumophila and the four newly sequenced PacBio genomes (see Table S5). If no close reference genome was found to a particular set of sequence reads (i.e., the kmer similarity to any reference was <90%), a *de novo* assembly of that isolate was used and added to the collection of reference genomes (see Table S5). This procedure allows SNP differences to be compared between closely related isolates for which the same reference genome will be selected. While it does not allow for comparison between isolates mapped to different reference genomes, the allocation to different references by KmerID already rapidly signals that isolates are distantly related. Details of the reference genomes used for each isolate are provided in Table S6.

Sequence reads were mapped to the chosen reference genome using BWA-MEM (49), and an in-house pipeline was used to identify SNPs using SAMtools (50), mpileup and BCFtools, as described previously (51). For a base to be called, at least 8 (and >75%) high-quality mapped reads with at least 3 on each strand must agree with the base call, the base quality score must be at least 50, and the mapping quality score must be at least 20. Reads that mapped equally well to more than one region were discarded to avoid repetitive regions. An isolate was considered typeable by this method if bases were called at a minimum of 90% of the positions in the reference genome. Second, in order to consider two isolates to belong to the same type, bases must be called in both isolates in at least 90% of variant positions identified among all isolates that are designated the same mapping reference. This excluded variants in mobile genetic elements, which were identified by comparing reference genomes with the Artemis Comparison Tool (ACT) (52) and manually curated using Artemis (53). This criterion ensured that isolates were not assigned the same type due to large amounts of missing data. Isolates considered nontypeable were still analyzed for the purpose of this study but would unlikely be used in a clinical setting.

Maximum likelihood trees were constructed from the variable sites using the general time-reversible (GTR) evolutionary model in RAxML v7.0.3 (54). A gamma correction was also applied to account for among-site rate variation. One hundred random bootstrap replicates were performed to analyze the support for nodes in a tree.

### Extended MLST.

Three hundred seventy L. pneumophila genomes (see Table S7) produced for this study or previously available, including a published set of isolates chosen to represent the known species diversity (42), were used to define the total core gene content of the species with Roary (55). This software automatically discards any genes shorter than 120 bp or without a start or stop codon. Further to this, we discarded any genes that were found in multiple copies in one or more genomes or that contained regions susceptible to sequence-specific errors (i.e., repeat regions) (56). A total of 1,455 core genes were defined that are present in all 370 genomes, using the Philadelphia-1 type strain genome (57) as a reference, and used in a core genome multilocus sequencing typing (cgMLST) scheme. Additionally, we randomly extracted nested subsets of 50, 100, and 500 genes from the 1,455 core genes to generate smaller cgMLST schemes. The genes used in each of these schemes are listed in Table S8.

In addition to the four cgMLST schemes above, we also tested a ribosomal MLST (rMLST) scheme (58), which uses 53 ribosomal genes universal among bacteria, as well as a recently described cgMLST scheme for L. pneumophila that uses 1,521 core genes (32). These six extended MLST schemes were set up using BIGSdb software (59). *De novo* assemblies were uploaded to the database, and the Genome Comparator tool was used to identify loci. This used a BLASTn search with a 70% identity cutoff, a 50% length cutoff, and a word size of 15. Any absent loci or loci that were truncated at a contig break were considered nontypeable. The remaining loci identified by BIGSdb were then subjected to further quality control (QC) testing, not currently available using BIGSdb software, using an in-house pipeline at PHE. Any loci that contained either 1 or more “N”s, or that contained less than 20 nucleotides, were considered nontypeable in the affected isolates. The raw sequence reads were also mapped to the extracted loci to validate the allele. Any loci that contained insufficient mapping coverage to validate all bases, or that possessed a discrepancy between the mapping data and assembly in one or more base positions, were considered nontypeable in the affected isolates. Only isolates with 100% typeable loci for a particular scheme were considered fully typeable. For the purpose of this analysis, isolates with 95 to 100% typeable loci were still analyzed with any nontypeable loci excluded, but these could not be used to yield a “type” in a clinical setting (although the number of allele differences could still be compared with other isolates). Isolates with <95% typeable genes for a particular scheme were not analyzed.

Distance matrices were computed, ignoring any nontypeable loci in affected isolates. These were used to construct neighbor-net trees that were inferred and visualized using SplitsTree4 (60).

### Gene presence/absence profiling.

Two hundred “accessory” genes that were present in 150 to 250 isolates (from the 370 isolates listed in Table S7), as defined by Roary (55), were selected for the gene presence/absence scheme (see Table S9). An in-house script (available from https://github.com/simonrharris/map_resistome) was used to determine whether each gene was present or absent in the *de novo* assembly of each isolate (or in the complete genomes of the Paris [40] and Lorraine [41] isolates). This attempted to map each gene to the *de novo* assembly of every isolate using SMALT (v0.7.4) and determined the percentage length and nucleotide identity of any match. Loci with ≥90% nucleotide identity and ≥90% of the length of the reference sequence were considered to be present, while loci with <90% nucleotide identity or <90% of the reference length were considered absent. Loci with ≥90% nucleotide identity and with a length between 20 and 90% of the reference sequence and at the end of a contig were considered nontypeable. As with the extended MLST schemes, only isolates with 100% typeable loci for a particular scheme were considered fully typeable and could be used to yield a “type” in a clinical setting, although for the purpose of this study, isolates with 95 to 100% typeable loci were still analyzed with the nontypeable loci excluded.

### kmer-based analysis.

Pairwise comparisons between isolates were calculated using KmerID from the *de novo* assemblies. The dissimilarity between any two isolates was scored using the Jaccard distance between the kmer sets (i.e., the number of distinct kmers shared between two assemblies over the number of distinct kmers in both assemblies together). kmers with a length of 18 nucleotides were used. Isolates were considered typeable via the kmer-based method if the *de novo* assemblies were within ±3 standard deviations (SD) of the mean length of all assemblies used in this study (i.e., between 3,215,920 bp and 3,736,908 bp) and the number of contigs comprised ≤3 SD over the mean (93 contigs). Isolates considered nontypeable were still analyzed in this study, although the results would unlikely be used in a clinical setting.

### Nucleotide sequence accession numbers.

Raw reads for all newly sequenced isolates were deposited in the European Nucleotide Archive (ENA) under study accession numbers ERP002503 and ERP014074. Accession numbers for individual isolates can be found in Tables S1, S2, and S3. Quality metrics and accession numbers for all *de novo* assemblies derived from Illumina data and used in this study are provided in Table S4 in the supplemental material. Reference sequences for the gene presence/absence scheme were deposited in the ENA under the accession numbers FJOD01000001 to FJOD01000200.

## RESULTS

A number of WGS-based typing methods were evaluated, including (i) a SNP/mapping-based method, (ii) extended MLST using various numbers of genes, (iii) gene presence or absence, and (iv) a kmer-based method. The extended MLST schemes tested include an rMLST scheme comprising 53 genes universal among all bacteria (58), a previously published cgMLST scheme for L. pneumophila using 1,521 genes (32), and newly designed cgMLST schemes using 50, 100, 500, or 1,455 core genes. Of the 1,455 core genes chosen for the full cgMLST scheme designed in this study, 1,114 (76.6%) were also used in the previously published scheme using 1,521 genes. All methods were evaluated in accordance with the guidelines produced by the ESGEM (36), and five performance criteria were considered: typeability (*T*), reproducibility (*R*), epidemiological concordance (*E*), discriminatory power (*D*), and stability (*S*). They were primarily tested using the standard typing panel consisting of the epidemiologically “unrelated” (*n* = 79) and “related” (*n* = 44) panels of L. pneumophila sg1 isolates (see Table S1 in the supplemental material). We also tested a further 229 isolates comprising isolates belonging to serogroups 6, 8, and 10, isolates from well-defined point source outbreaks, and multiple isolates from some of the major disease-associated STs (see Table S2).

### Typeability.

In order to type isolates using the SNP-based method, a close reference genome was determined for each of the isolates using KmerID (see Materials and Methods). This resulted in the use of 25 reference genomes for the 106 typing panel isolates and 27 reference genomes for the total collection of 335 isolates (see Table S6). This large number of reference genomes reflects the high diversity within L. pneumophila. Isolates mapped to different reference genomes automatically constituted different types, while isolates mapped to the same reference genome were subdivided into types based on the number of SNP differences. To be considered typeable, however, isolates must first map to ≥90% of the length of the respective reference genome. Second, for any isolates categorized into the same type using a particular similarity threshold, we should be able to call ≥90% of total variant positions (i.e., those identified in all isolates mapped to the same reference), excluding those in mobile genetic elements. This was exceeded by all typing panel isolates (*T* = 1.0) and 225 (98.3%) of the 229 additional isolates (*T* = 0.983), including all non-sg1 isolates (Table 1; see also Tables S6 and S10).

**TABLE 1:** Typeability of the WGS-based methods using the typing panel (*n* = 106) and all isolates analyzed in this study (*n* = 335)^*a*^

Typing method (no. of genes)	Typeability (*T*)		Gene-based schemes (typing panel isolates only)			
	Typing panel only (*n* = 106)	All isolates (*n* = 335)	% of isolates with ≥98% genes typeable	% of isolates with ≥95% genes typeable	No. (%) of genes with 100% typeability	No. (%) of genes with <100% typeability
SNP based	1.0	0.988	NA	NA	NA	NA
rMLST (53)	0.906	0.899	100	100	50 (94.3)	3 (5.7)
cgMLST (50)	0.991	0.988	99.1	100	48 (96.0)	2 (4.0)
cgMLST (100)	0.991	0.988	100	100	98 (98.0)	2 (2.0)
cgMLST (500)	0.972	0.973	100	100	495 (99.0)	5 (1.0)
cgMLST (1,455)	0.868	0.916	100	100	1,444 (99.2)	11 (0.8)
cgMLST (1,521)	0.396	0.379	100	100	1,462 (96.1)	59 (3.9)
Gene presence or absence	0.415	0.522	98.1	100	179 (89.5)	21 (10.5)
kmer based	1.0	0.997	NA	NA	NA	NA

a Typeability of isolates using the SNP-based method was calculated assuming that one or more differences between isolates constitute different types (as different thresholds can alter the typeability). NA, not applicable.

The six extended MLST schemes were set up using BIGSdb software, and loci were identified from the *de novo* assemblies using the integrated Genome Comparator tool. Using a single virtual machine with 1 gigabyte (GB) of random-access memory (RAM), the lengths of time taken for the loci to be determined by this tool were ∼1 min per isolate using the 50-gene scheme and ∼20 to 25 min per isolate using the 1,455-gene or 1,521-gene scheme. At this stage, all loci were identified in every typing panel isolate, with the exception of two loci from the 1,521-gene cgMLST scheme that were absent or truncated (therefore considered nontypeable) in two isolates (see Table S11). Furthermore, all loci were identified in 94.8% (1,521-gene scheme) to 100% (50-gene scheme) of the additional 229 isolates. All extracted loci were then subjected to an in-house QC pipeline at PHE that included validation of alleles by mapping data and the identification of alleles containing one or more “N”s or consisting of <20 bases. These validation steps are not currently implemented in the Genome Comparator tool of BIGSdb. A substantially higher number of loci were considered nontypeable by these criteria (see Table S11), and in all schemes, at least one typing panel isolate lacked a full profile. Generally, the more genes included in an extended MLST scheme, the lower the proportion of fully typeable isolates, with 99.1% of the typing panel isolates producing a full profile with the 50-gene scheme (*T* = 0.991) and only 86.8% of the isolates with the 1,455-gene scheme (*T* = 0.868) (Table 1). The relatively low typeability score of the rMLST scheme (*T* = 0.906), which uses 53 ribosomal genes, can be mostly explained by the inability of the QC stage to validate a particular gene (*lpg0328*) in 10 isolates due to the absence of adequate flanking regions in the assemblies, and it is therefore partially attributable to our method. Furthermore, in the previously published 1,521-gene cgMLST scheme, only 39.6% of isolates were fully typeable (*T* = 0.396). The proportion of the additional 229 isolates that were fully typeable by each of the extended MLST schemes was similar to that for the typing panel isolates, and *T* values for all 335 isolates are provided in Table 1.

Despite the significant numbers of isolates lacking a full extended MLST profile, 94.3 to 99.2% of genes belonging to each of the schemes were typeable in all typing panel isolates (Table 1). Furthermore, ≥96% of genes were typeable in every typing panel isolate by each scheme. A relatively small number of genes were thus responsible for incomplete profiles; only 61 genes from a total of 1,865 genes across all six extended MLST schemes were nontypeable in ≥1 isolates (see Table S12). Fifty-nine of these belong to the previously published 1,521-gene cgMLST scheme (including 49 exclusively), while 11 are part of the newly designed 1,455-gene cgMLST scheme. Of the total 61 nontypeable genes, 25 were considered nontypeable in >1 of the typing panel isolates, suggesting a problem with the choice of gene rather than a specific isolate, while 36 were nontypeable in one isolate only. We tested whether the ability of sequence data to yield a full profile in extended MLST schemes could be predicted by either the mean mapping coverage or the number of contigs or N50 values of the assemblies. However, no significant differences were found between isolates that produced a full profile in all six of the extended MLST schemes and those that produced an incomplete profile in ≥1 (Student's unpaired *t* test, *P* > 0.05) (see Table S13), suggesting that these metrics are not effective markers for typeability.

The gene presence/absence-based scheme involved scoring the presence or absence of 200 accessory genes from the *de novo* assemblies and constructing MLST-like profiles (using “0”s and “1”s) to yield a type. Any partially present genes identified on contig boundaries were considered nontypeable (see Materials and Methods). Only 41.5% of typing panel isolates and 57.2% of the additional 229 isolates possessed a full profile (see Table S14), yielding a low typeability value for all 335 isolates (*T* = 0.522) (Table 1). However, 89.5% of genes were typeable in all typing panel isolates and ≥97.5% of genes were typeable in each isolate, suggesting that a small number of problematic genes may be responsible for the low overall typeability, as with the extended MLST schemes. Indeed, we found a total of 21 genes that were nontypeable in ≥1 isolate, 15 of which were nontypeable in ≥2 isolates (see Table S15).

Isolates were compared to each other using the kmer-based method by calculating a dissimilarity score between each pair (see Materials and Methods) and clustering isolates with scores below a particular threshold. All typing panel isolates and all but one of the additional 229 isolates (H063860003) were considered typeable by the kmer-based method (*T* = 0.997) since the lengths of the *de novo* assemblies were within the required range (i.e., ±3 SD of the mean of all assemblies used in this study) and the number of contigs did not exceed the maximum permitted (i.e., 3 SDs over the mean) (Table 1; see also Tables S4 and S10).

### Reproducibility.

**FIG 1:**
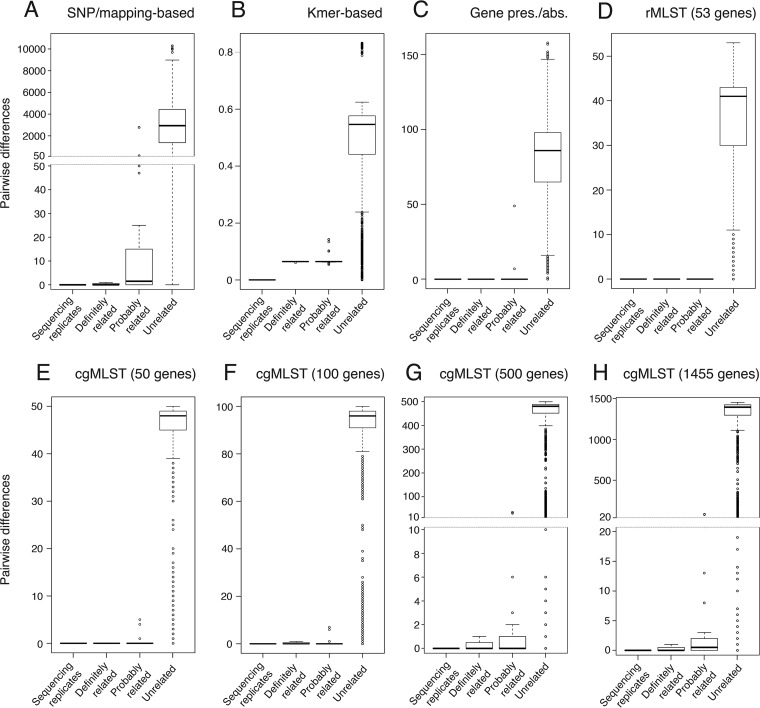
Box plots with pairwise differences between typing panel isolates as calculated by each of the WGS-based methods. Included are sequencing replicates (6 pairs), “definitely related” isolates (10 isolates comprising 4 sets), “probably related” isolates (34 isolates comprising 13 sets), and 79 epidemiologically “unrelated” isolates.

Six typing panel isolates (EUL 27, 33, 69, 75, 92, and 111) were sequenced twice in different runs, using the same methods at the same sequencing center to assess the reproducibility of the WGS-based methods (Fig. 1 and Table 2). The replicate isolates were chosen randomly and represent a range of STs. No differences were found among pairs using either the SNP-based method or the extended MLST and gene presence/absence schemes once the additional QC steps were implemented. The kmer-based dissimilarity scores calculated between sequencing replicates were extremely low (<0.001) and of the same order of magnitude in all six pairs. None were zero, however, suggesting very minor differences between the *de novo* assemblies. All methods were assigned reproducibility values (*R*) of 1.0 (Table 2).

**TABLE 2:** The number of differences identified between sequencing replicates using each of the WGS-based methods^*a*^

EUL no.	No. of differences between replicates								Difference between replicates by kmer-based method
	SNP-based method	rMLST (53 genes)	cgMLST (50 genes)	cgMLST (100 genes)	cgMLST (500 genes)	cgMLST (1,455 genes)	cgMLST (1,521 genes)	Gene presence or absence	
27	0	0 (0)	0 (0)	0 (0)	0 (0)	0 (0)	1 (0)	0 (0)	0.00029
33	0	0 (0)	0 (0)	0 (0)	0 (0)	0 (0)	0 (0)	0 (0)	0.00050
69	0	0 (0)	0 (0)	0 (0)	0 (0)	0 (0)	0 (0)	1 (0)	0.00051
75	0	0 (0)	0 (0)	0 (0)	0 (0)	0 (0)	1 (0)	0 (0)	0.00028
92	0	0 (0)	0 (0)	0 (0)	0 (0)	0 (0)	0 (0)	1 (0)	0.00052
111	0	0 (0)	0 (0)	0 (0)	0 (0)	0 (0)	4 (0)	0 (0)	0.00064
Reproducibility	1	1	1	1	1	1	0.5 (1)	0.66 (1)	1

a For each of the extended MLST schemes, both the number of differences identified by BIGSdb software (pre-QC) and the number identified after all alleles are validated by our QC stages are given, the latter in parentheses. For the gene presence/absence method, the numbers of differences identified before and after the exclusion of partially present genes on contig boundaries are given, the latter in parentheses. The difference between replicates as calculated by the kmer-based method is expressed using the Jaccard dissimilarity score.

### Epidemiological concordance.

We investigated the epidemiological concordance (*E*) of 17 sets of epidemiologically related isolates from the typing panel, including 4 definitely related sets (subdivision I) and 13 probably related sets (subdivision II) (see Table S1). The definitely related isolates include replicates and multiple isolates from the same patient, while the probably related isolates comprise clinical and environmental isolates that were chosen by our colleagues as epidemiologically related (i.e., isolated at similar times and places and/or associated with a point source) but not necessarily genotypically related. Previous studies have shown that all are concordant by MAb subgrouping, restriction fragment length polymorphism (RFLP) analysis, and AFLP analysis (37, 38) as well as 3- and 6-allele SBT. However, one set (EUL 37, 44, and 45) was later revealed to be discordant by the current gold standard 7-allele SBT (EUL 37 and 44 are ST1 and EUL 45 is ST72) and thus may have been falsely linked. For the purpose of this analysis, we included all isolates considered nontypeable by one or more methods, with the exception of isolates with <95% typeable loci in the gene-based schemes. This resulted in the inclusion of all typing panel isolates but also means that the epidemiological concordance values calculated may be slightly overestimated.

**FIG 2:**
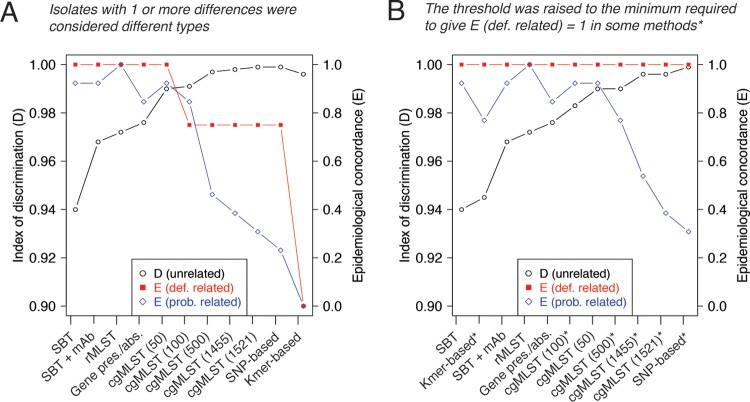
(A) Index of discrimination (*D*) and epidemiological concordance (*E*) of each of the current and WGS-based methods based on the 106 typing panel isolates. Isolates were classified as the same type if they shared no differences and a different type if they shared 1 or more differences, except using the kmer-based method, where isolates were categorized into types using single-linkage clustering with a threshold equal to the maximum difference detected between sequencing replicates. (B) *D* and *E* values of each of the current and WGS-based methods when single-linkage clustering was used for some methods with a threshold that maintains the *E* of at least definitely related isolates at 1. The threshold is one allele difference using the cgMLST schemes with 100 or more genes, one SNP using the SNP-based method, and 0.065 using the kmer-based method. Using the rMLST scheme, the 50-gene cgMLST scheme, and the gene presence/absence scheme, isolates were classified as different types if they shared 1 or more differences (as in panel A).

Using each WGS-based method, we first classified isolates into the same type if they shared no differences and different types if they shared one or more differences. This is the simplest and most practical method of assigning types and also allows maximum discrimination to be achieved with any given method. One exception was the kmer-based approach, which could detect a difference, albeit extremely small, between sequencing replicates, and thus, we categorized isolates into types using single-linkage clustering with a threshold equal to the maximum difference detected between replicates (0.00064). This means that any isolates with dissimilarity scores equal to or lower than the threshold would be considered the same type, along with any other isolates that are linked to the cluster through at least one isolate (thus permitting chains to arise). Based on these criteria, we calculated the epidemiological concordance (*E*) of the epidemiologically related sets (Table 3 and Fig. 2A). All four definitely related sets were concordant using only rMLST, 50-gene cgMLST, and the gene presence/absence method (*E* = 1.0).

**TABLE 3:** Index of discrimination and epidemiological concordance of the current and tested WGS-based typing methods^*a*^

Typing method	Threshold	No. of types	Index of discrimination	Epidemiological concordance score (no. of sets with concordance/total no. of sets)		
				Subdivision I (definitely related)	Subdivisions I and II (definitely related and probably related)	Subdivisions I and II excluding EUL 37, 44, and 45^*b*^
SBT	0	40	0.940	1 (4/4)	0.941 (16/17)	1 (16/16)
SBT + MAb subgrouping	0	43	0.968	1 (4/4)	0.941 (16/17)	1 (16/16)
SNP based	0	78	0.999	0.750 (3/4)	0.353 (6/17)	0.375 (6/16)
	1	77	0.999	1 (4/4)	0.471 (8/17)	0.500 (8/16)
rMLST (53 genes)	0	44	0.972	1 (4/4)	1 (17/17)	1 (16/16)
cgMLST (50 genes)	0	57	0.990	1 (4/4)	0.941 (16/17)	1 (16/16)
cgMLST (100 genes)	0	59	0.991	0.750 (3/4)	0.824 (14/17)	0.875 (14/16)
	1	53	0.983	1 (4/4)	0.941 (16/17)	1 (16/16)
cgMLST (500 genes)	0	71	0.997	0.750 (3/4)	0.529 (9/17)	0.563 (9/16)
	1	67	0.990	1 (4/4)	0.824 (14/17)	0.875 (14/16)
cgMLST (1,455 genes)	0	75	0.998	0.750 (3/4)	0.471 (8/17)	0.500 (8/16)
	1	72	0.996	1 (4/4)	0.647 (11/17)	0.688 (11/16)
cgMLST (1,521 genes)	0	76	0.999	0.750 (3/4)	0.412 (7/17)	0.438 (7/16)
	1	72	0.996	1 (4/4)	0.529 (9/17)	0.563 (9/16)
Gene presence or absence	0	53	0.976	1 (4/4)	0.882 (15/17)	0.938 (15/16)
kmer based	0.00064	71	0.996	0 (0/4)	0 (0/17)	0 (0/16)
	0.065	41	0.945	1 (4/4)	0.824 (14/17)	0.875 (14/16)

a The number of types and discrimination (*D*) values were calculated using 79 epidemiologically unrelated isolates from the typing panel. The epidemiological concordance (*E*) values were calculated using a total of 44 epidemiologically related isolates from the typing panel that include both definitely related (subdivision I) and probably related (subdivision II) isolates.

b The set of probably related isolates comprising EUL 37, 44, and 45 is not epidemiologically concordant via 7-allele SBT, suggesting that these may be falsely linked isolates, and thus, *E* values were also calculated excluding this set.

**FIG 3:**
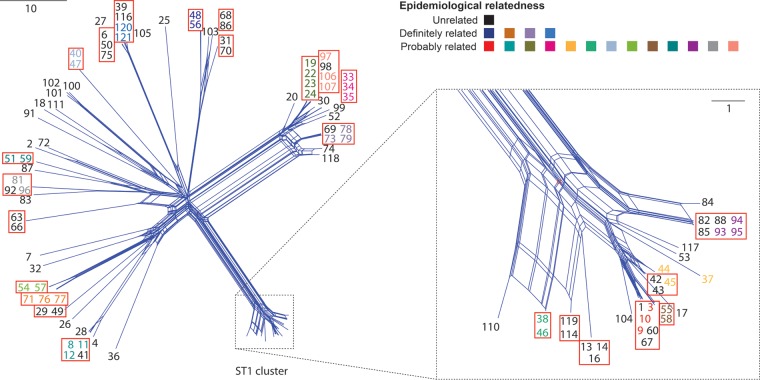
Neighbor-net tree of the 106 typing panel isolates constructed using the 50-gene cgMLST scheme. All isolates are colored by their epidemiological relatedness as indicated in the key. Isolates belonging to the same type (i.e., with no allele differences) are enclosed in a red box. The ST1 cluster, comprising both ST1 isolates and isolates derived from ST1, is shown at a higher resolution on the right. The scale bars indicate the number of allelic differences.

The 13 probably related sets were also all concordant using the rMLST scheme, including the set comprising EUL 37, 44 and 45, which is discordant by SBT. All probably related sets excluding EUL 37, 44, and 45, were concordant using the 50-gene cgMLST scheme. The epidemiological concordance achieved by the 50-gene cgMLST scheme is demonstrated in Fig. 3 using a neighbor-net tree inferred from the pairwise differences between isolates. However, as more genes were subsequently included in the cgMLST schemes, the number of probably related sets that were concordant decreased. The SNP-based approach also fared poorly, with just 5 of 13 probably related sets concordant, and the kmer-based approach achieved concordance with no sets at this threshold. The gene presence/absence scheme performed well, however, with 11 of the 13 sets concordant.

Since a typing scheme should maintain complete epidemiological concordance for at least definitely related sets of isolates, we next used single-linkage clustering with the lowest threshold possible for each of the methods to class all definitely related isolates as the same type (Fig. 2B). This meant allowing 1 allele difference in the cgMLST schemes using 100 or more genes, 1 SNP difference with the SNP-based method, and a threshold of 0.065 with the kmer-based method. Using each of these methods at the newly defined thresholds, the number of probably related sets of isolates that are concordant increased, particularly with the kmer-based scheme, by which 11 of 13 sets are concordant. However, a number of sets remained discordant, including 9 by the SNP-based method.

We therefore investigated the number of differences identified among probably related sets that are discordant using one or more of the WGS-based methods to determine how much further thresholds would need to be increased to reach full concordance (see Table S16). This also allowed us to identify any potentially falsely linked isolates with differences much larger than the majority. Indeed, the number of SNPs found between probably related isolates was highly variable, ranging from less than five to several thousand. The results suggest that isolates from the set comprising EUL 37, 44, and 45 were incorrectly linked, with SNP differences ranging from 179 to 2,786. A clinical isolate (EUL 19) from the set comprising EUL 19, 22, 23, and 24 may also have been falsely linked, since it differs by 25 to 50 SNPs from the other three isolates that otherwise differ by only 0 to 1 SNPs. Disregarding these two sets, SNP differences between isolates from the remaining 11 sets range from 0 to 16. Using the cgMLST schemes (with 100 or more genes), the set comprising EUL 37, 44, and 45 also showed substantially larger differences than the majority of sets, with up to 82 differences observed between isolates using the 1,455-gene cgMLST scheme. Interestingly, between 0 and 3 differences are observed within the set comprising EUL 19, 22, 23, and 24, a range that is similar to those seen in other probably related sets. Disregarding the set comprising EUL 37, 44, and 45, the ranges of allele differences observed in the cgMLST schemes with 100, 500, 1,455, and 1,521 genes are 0 to 1, 0 to 3, 0 to 8, and 0 to 13, respectively. The two sets that are discordant using the gene presence/absence method are also EUL 37, 44, and 45 and EUL 19, 22, 23, and 24, which contain up to 7 and 49 differences, respectively, showing that they differ by gene content as well as SNPs. Lastly, the kmer-based approach, using a threshold of 0.065, achieved concordance with all sets with the exception of three probably related sets, which include the two possibly falsely linked sets (EUL 37, 44, and 45 and EUL 19, 22, 23, and 24) and the set comprising EUL 51 and 59, which had a dissimilarity score of 0.066, only slightly higher than the threshold.

**FIG 4:**
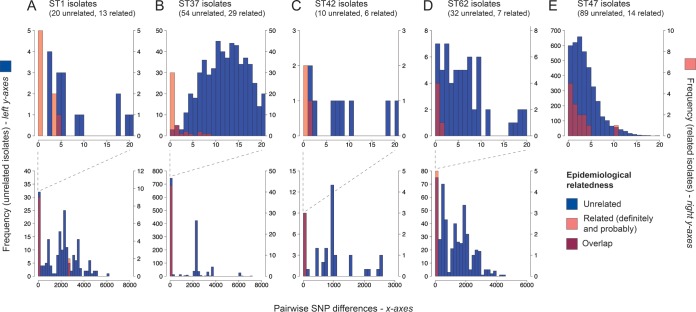
Histograms showing pairwise SNP differences between epidemiologically unrelated and related isolates belonging to some of the major disease-associated sequence types (STs): ST1 (A), ST37 (B), ST42 (C), ST62 (D), and ST47 (E). In panels A to D, the top histogram shows pairwise SNP differences up to 20 only, while the bottom portion presents the full range. The maximum pairwise SNP difference within the ST47 isolates is <20 SNPs, and thus, only one illustration is shown (E). Left and right *y* axes represent the frequency of epidemiologically unrelated and related isolates, respectively. The epidemiologically unrelated and related isolates are colored as indicated in the key at the bottom right.

**FIG 5:**
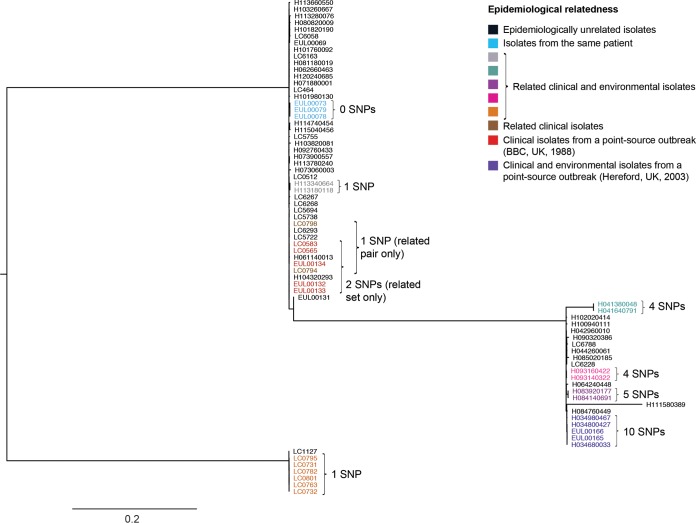
Maximum likelihood tree of 74 ST37 isolates constructed using 8,648 variable positions. Isolates are colored by their epidemiological relatedness as indicated in the key. The total number of SNPs identified between isolates of each epidemiologically related set is indicated. The scale shows the number of SNPs per variable site.

In addition to investigating the sets in the typing panel, we investigated the number of differences between isolates from three outbreaks in the United Kingdom with well-defined point sources (BBC, Portland Place, 1988; Barrow-in-Furness, 2002; and Hereford, 2003) and a further 17 epidemiologically related sets to determine whether (and at what threshold) each would be considered concordant by the WGS-based methods. The results are summarized in Table S17. Using each of the methods to their maximum discriminatory potential (i.e., allowing for no differences between isolates of the same type or using a threshold of 0.00064 with the kmer-based method), epidemiological concordance for all three point source outbreak sets and the majority of additional related sets was achieved using rMLST, cgMLST with either 50 or 100 genes, and the gene presence/absence method, but not with the cgMLST schemes using 500 or more genes or the SNP-based or kmer-based method. Pairwise SNP differences between isolates from sets belonging to major disease-associated STs are shown in Fig. 4. The range of SNP differences (0 to 10) observed between related ST37 isolates is also shown in the context of other epidemiologically unrelated isolates in an SNP-based phylogeny of 74 ST37 isolates (Fig. 5).

### Discriminatory power.

We investigated the discriminatory power of each of the WGS-based methods using 79 epidemiologically unrelated isolates from the typing panel (see Table S1). We first clustered isolates into types, allowing for no differences between isolates of the same type (or no greater differences than those observed between sequencing replicates in the case of the kmer-based method) and subsequently using the previously defined thresholds that maintain the epidemiological concordance of at least definitely related isolates. The results are summarized in Table 3 and Fig. 2. All methods had a higher index of discrimination (*D*) than the current gold standard, SBT, and the combination of SBT and MAb subgrouping, which is also frequently used. Individual *D* values for all genes used in the extended MLST and gene/presence absence schemes are provided in Tables S18, S19, and S20. It should be noted that as isolates with 95 to 100% typeable loci in the gene-based schemes were included in the analysis, some indices of discrimination might be slight underestimates.

Finally, we tested how well the WGS-based methods could differentiate between epidemiologically unrelated isolates of some of the major disease-associated STs (1, 37, 42, 47, and 62) (see Table S1 and Table S2), which, by definition, cannot be currently split by the current method, SBT. For each method, we allowed for no differences between isolates of the same type (or no greater differences than those observed between sequencing replicates with the kmer-based method) and thus calculated the maximum possible discrimination achieved by each method. We excluded one ST1 isolate, H034800423, from all analyses, since up to 30% of genes were nontypeable in the extended MLST schemes. The results are summarized in Table 4. Interestingly, we found that while all methods could mostly separate epidemiologically unrelated isolates of the same ST into further types and the most discriminatory methods (e.g., SNP-based) could almost completely differentiate between all isolates, some isolates were highly similar (i.e., <20 SNPs) and even identical (Fig. 4). This is most apparent in the ST47 lineage, in which all isolates share fewer than 20 SNPs and 16 epidemiologically unrelated isolates (from a total of 89) are identical (Fig. 4E). It is also illustrated by an SNP-based tree of 74 ST37 isolates, which shows the division of isolates into three highly clonal clusters in which some epidemiologically unrelated isolates are interspersed between related isolates (Fig. 5).

**TABLE 4:** Number of types that epidemiologically unrelated isolates from major disease-associated STs are divided into and indices of discrimination achieved by each of the WGS-based methods^*a*^

ST (no. of unrelated isolates)	No. of types/index of discrimination								
	SNP-based method	rMLST (53 genes)	cgMLST (50 genes)	cgMLST (100 genes)	cgMLST (500 genes)	cgMLST (1,455 genes)	cgMLST (1,521 genes)	Gene presence or absence	kmer-based method
1 (20)	20/1	4/0.721	10/0.879	12/0.911	17/0.979	20/1.00	19/0.995	5/0.668	20/1
37 (54)	53/0.999	9/0.473	10/0.368	12/0.426	36/0.909	50/0.997	49/0.999	13/0.592	54/1
42 (10)	10/1	4/0.778	4/0.733	5/0.800	10/1.00	10/1.00	10/1.00	5/0.667	10/1
47 (89)	66/0.958	1/0	2/0.022	2/0.022	18/0.365	41/0.857	40/0.848	5/0.229	89/1
62 (32)	30/0.994	4/0.333	8/0.790	12/0.849	21/0.942	28/0.980	31/0.998	15/0.915	27/0.982

a Isolates were classified as the same type if they shared no differences and a different type if they shared 1 or more differences, except when we used the kmer-based method, where isolates were categorized into types using single-linkage clustering with a threshold equal to the maximum difference detected between sequencing replicates.

### Stability.

The three definitely related sets comprising isolates from the same patient were also used to assess the stability of each of the WGS-based methods. These comprised sets with two isolates sampled 15 days apart (EUL 48 and 56), three isolates sampled either via direct plating, via amoebal coculture or from a fecal sample (EUL 71, 76, and 77), and three isolates picked from single colonies on a primary isolation plate (EUL 73, 78 and 79) (see Table S1). Isolates belonging to two of these sets (EUL 48 and 56 and EUL 73, 78, and 79) were identical (therefore stable) by all methods with the exception of the kmer-based method, in which they contained differences, albeit small but larger than those seen between sequencing replicates (see Table S16). Meanwhile, isolates belonging to the third set (EUL 71, 76, and 77) were stable only by rMLST, 50-gene cgMLST, and the gene presence/absence method (see Table S16).

## DISCUSSION

In recent years, WGS-based analysis has been shown to provide the ultimate resolution for studying the evolution, population structure, and transmission of important bacterial pathogens. It also represents a highly promising molecular typing tool that could supplement or even replace current methods. In some public health laboratories, WGS now costs as little as SBT, the current gold standard method for the epidemiological typing of L. pneumophila, but yields considerably more information. The major challenge to its implementation is now posed by the requirement for specialist computing infrastructure and bioinformatics expertise as well as the need for a scalable and portable classification scheme. The majority of WGS-based bacterial typing schemes proposed so far, including two for L. pneumophila, have been based on a scaled-up MLST (cgMLST) approach (32, 35, 61–63), which allows for easy standardization and exchange of data. While some SNP-based schemes have also been tested (64), there have been few studies that have compared the performances of different approaches. In this study, we used the guidelines produced by the ESGEM (36) considering the typeability, reproducibility, epidemiological concordance, discriminatory power, and stability of several WGS-based typing methods, with the aim of determining the optimal method for future development. To our knowledge, this is the first study to formally evaluate WGS for a bacterial typing scheme based on these criteria.

For each of the tested methods, we determined specific criteria that must be met for isolates to be deemed typeable, which included criteria aimed to reject isolates with low-quality sequence data. Thus, typeability scores produced in this study are linked both to isolates and to sequence data and would have the ability to change on resequencing. Overall, 98.8% and 99.7% of isolates were deemed typeable by the SNP- and kmer-based methods, respectively. Between 86.8% and 99.1% of typing panel isolates contained a full set of typeable genes using the rMLST scheme or the cgMLST schemes with 50, 100, 500, or 1,455 genes. However, using the previously published cgMLST scheme with 1,521 core genes, and the newly described gene presence/absence scheme, just 39.6% and 41.5% of typing panel isolates, respectively, were fully typeable. The low typeability of the gene presence/absence-based scheme compared with those of the majority of extended MLST schemes might reflect a higher proportion of accessory genes containing regions that are difficult to sequence or assemble. Across all gene-based schemes, however, further investigation revealed that the majority of genes were indeed typeable across all isolates and just a small subset of genes belonging to each scheme yielded typeability issues. Many of these were deemed nontypeable in more than one isolate, suggesting a problem with the choice of gene (e.g., because of repetitive regions making it difficult to sequence) rather than specific problems with an isolate or sequence data quality. Therefore, with carefully chosen core genes and the removal of problematic genes, we believe that newly designed gene-based schemes would be capable of achieving higher typeability than the values obtained in this study. Our evaluation of extended MLST schemes also highlighted the importance of further validating the alleles extracted from the *de novo* assemblies involving steps unavailable in BIGSdb software and not currently part of standard practice. For example, the use of mapping data to search for base discrepancies, as well as the identification of any alleles containing Ns or deletions, highlighted a significant number of alleles not identified by BIGSdb that should not be used in comparisons between isolates or to yield a type.

The resequencing of six typing panel isolates, using the same DNA but different sequencing libraries, indicated that all WGS-based methods are highly reproducible, given good-quality sequence data produced using the same methodologies and the QC filters as implemented in this study. This supports the findings from a previous study that showed an average difference of ≤0.39 (SNPs or indels) between the same type of replicates (64). Further work is required to determine the reproducibility of WGS-based methods when isolates are sequenced at different centers, using different library preparation and sequencing methodologies, and at different times (e.g., after prolonged storage or multiple passages of isolates).

All WGS-based methods were capable of achieving higher discrimination between epidemiologically unrelated isolates than the current gold standard method, SBT. Using the 79 unrelated typing panel isolates, the indices of discrimination achieved by the rMLST scheme (*D* = 0.972) and the gene presence/absence scheme (*D* = 0.976), however, were not much greater than that achieved using SBT in combination with MAb subgrouping (*D* = 0.968), and therefore neither provide much added benefit. There was a substantial increase in the discriminatory power achieved by the 50-gene cgMLST scheme (*D* = 0.990), and the addition of a further 50 genes to make the 100-gene scheme only marginally increased the discriminatory power from this (*D* = 0.991). The cgMLST schemes using either 500, 1,455, or 1,521 genes and the SNP-based and kmer-based methods could achieve almost complete differentiation between unrelated isolates from the typing panel, and even very high differentiation between epidemiologically unrelated isolates belonging to some of major disease-associated STs.

Inevitably, there is a trade-off between discriminatory power and epidemiological concordance. The least discriminatory methods, such as the rMLST and the gene presence/absence method, achieved excellent epidemiological concordance, classifying all definitely related typing panel isolates and isolates from well-described point source outbreaks into single types (allowing for no differences between isolates of the same type). The cgMLST schemes with 50 or 100 genes, which are more discriminatory, also achieved good epidemiological concordance, although one definitely related set (EUL 71, 76, and 77) was split up by the 100-gene scheme due to the presence of a single SNP. On the other hand, the highly discriminatory methods (i.e., the cgMLST schemes with 500 or more genes and the SNP- and kmer-based methods) achieved poor epidemiological concordance if no differences were allowed between isolates of the same type. Thus, in order to use these methods in a typing scheme and continue to categorize isolates into a useful number of types, a threshold would need to be determined for each, specifying the number of differences allowed between isolates of a particular type. In order for at least the “definitely related” typing panel isolates to be considered the same type, we needed to allow for one SNP in the SNP-based method, one allele in the cgMLST schemes with 100 or more genes, and a threshold of 0.065 using the kmer-based method. However, with these cutoffs, large proportions of probably related isolates are not considered the same type, and thus, the thresholds would likely need increasing further. In the previously published study describing the 1,521-gene cgMLST scheme, the authors suggested a threshold of four alleles (32), and in another SNP-based analysis, the authors discovered up to 15 SNPs between point source outbreak isolates (29). However, the use of thresholds would require implementation of a clustering algorithm that would most likely need to be rerun each time a new isolate was added to the collection. Clustering could also have the limitation of drawing arbitrary boundaries between types, leading to the misrepresentation of relationships (i.e., isolates on the boundary of a cluster could be more similar to those from other clusters than the same cluster). Therefore, we propose that types should ideally be defined using a less discriminatory method that can maintain good epidemiological concordance while classifying “identical” isolates as the same type and isolates with ≥1 difference as different types.

A further consideration in the design of a new WGS-based typing scheme for L. pneumophila is the ability to maintain backwards compatibility with the current method, SBT. This is important, first, because it is not always possible to isolate L. pneumophila and perform WGS (for example, due to sample contamination with background flora). However, in such cases, typing can be performed using nested-PCR-based SBT directly from primary samples (http://bioinformatics.phe.org.uk/legionella/legionella_sbt/php/protocols/ESGLI%20NESTED%20SBT%20GUIDELINE%20v2.0.pdf). A second reason is that WGS may not become routine in many public health laboratories for some years. Thus, regardless of the WGS-based method used, the typing procedure should also involve determining the seven SBT alleles. This could be performed automatically if the seven genes were part of a cgMLST scheme. However, as noted by Moran-Gilad and colleagues (32), a current problem is the inability to consistently determine the *mompS* allele number from short-read WGS data due to the presence of multiple gene copies that are occasionally different. To maintain full backwards compatibility, it may therefore be necessary to continue to perform PCR and Sanger sequencing of this one gene, regardless of the WGS-based methodology used.

Overall, our results suggest that L. pneumophila could be most usefully typed using a cgMLST scheme with approximately 50 genes. This offers the best compromise between improving upon the discrimination obtainable by current methods and maintaining good epidemiological concordance (without the need to use thresholds or clustering methods). However, we also propose that such a scheme could be used as part of a larger hierarchical scheme comprised of the 7 SBT genes and 50, 100, 500, and a full set of core genes (∼1,500). Isolates could be assigned a “type” at each level, allowing the extremely high discrimination offered by WGS to be exploited when needed while appreciating that some differences between related isolates are to be expected when using higher numbers of genes.

Together with members of the ESGLI, we are designing, testing, and implementing a new scheme that can take account of the performance of individual genes as calculated in this study, as well as those genes considered nontypeable among our collection of isolates, to determine new gene sets. This will also require the development of a central database in which alleles and types can be assigned and stored for use by the research and public health community. The result should be an improved scheme that can type all, or almost all, isolates given good-quality sequence data and can ultimately resolve a higher proportion of point source outbreaks caused by L. pneumophila than current methods.

## Supplementary Material

Supplemental material
