# Supplementary material for: Evaluation of an Optimal Epidemiological Typing Scheme for Legionella pneumophila with Whole-Genome Sequence Data Using Validation Guidelines
Source: J Clin Microbiol. 2016 Jul 25;54(8):2135–48. doi: 10.1128/JCM.00432-16 (PMC4963484; doi:10.1128/JCM.00432-16)
Supplement: Supplemental material [file supp_54_8_2135__index.html]

Supplemental material 

# Evaluation of an Optimal Epidemiological Typing Scheme for Legionella pneumophila with Whole-Genome Sequence Data Using Validation Guidelines

## Supplemental material

- Supplemental file 1 -

  Tables S1 (ESGLI standard typing panel of 106 isolates of *Legionella pneumophila* sg1 from 10 European countries, comprising epidemiologically “unrelated” and “related” panels), S2 (Additional 229 clinical and environmental isolates used in evaluation of WGS-based methods), S3 (Sequencing statistics for 4 isolates sequenced on the Pacific Biosciences RSII sequencer), S4 (Quality metrics and accession numbers for all *de novo* assemblies [derived from Illumina data] used in this study), S5 (Reference genomes used in SNP-based analysis), S6 (Reference genomes used for mapping of all isolates in this study and coverage achieved), S7 (370 *L. pneumophila* isolates used to define the total core gene content of the species), S8 (Genes used in cgMLST schemes with 50, 100, 500, or 1,455 core genes), S9 (200 “accessory” genes used in the gene presence/absence scheme), S10 (Summary of sequencing statistics for typing panel isolates and all isolates used in this study [excluding the 2 complete genomes]), S11 (Number of typable loci in each isolate for each extended MLST scheme), S12 (61 untypable genes in 6 extended MLST schemes and number of affected isolates in the typing panel), S13 (Mean and range of mapping coverage, number of contigs, and N50 values of isolates that produce complete or incomplete profiles in extended MLST schemes), S14 (Number of accessory genes scored as present, absent, or untypable using the gene presence/absence typing method), S15 (21 genes that were scored as untypable in 1 or more typing panel isolates using the gene presence/absence typing method and number of affected typing panel isolates), S16 (Number of differences identified between isolates from epidemiologically “related” sets in the typing panel using each of the WGS‐based methods), S17 (Number of differences between isolates belonging to an additional 20 epidemiologically “related” sets, as analyzed by each of the WGS‐based methods), S18 (Indices of discrimination for 53 ribosomal genes, calculated using 79 epidemiologically “unrelated” isolates from the typing panel), S19 (Indices of discrimination for 200 accessory genes, calculated using 79 epidemiologically “unrelated” isolates from the typing panel), and S20 (Indices of discrimination for 1,455 core genes, calculated using 79 epidemiologically “unrelated” isolates from the typing panel)

  PDF, 3.4M
