# Supplementary material for: Evaluation of an Optimal Epidemiological Typing Scheme for Legionella pneumophila with Whole-Genome Sequence Data Using Validation Guidelines
Source: J Clin Microbiol. 2016 Jul 25;54(8):2135–48. doi: 10.1128/JCM.00432-16 (PMC4963484; doi:10.1128/JCM.00432-16)
Supplement: Supplemental material [file JCM.00432-16_zjm999095097so1.pdf]

Supplemental Material

Evaluation of an optimal epidemiologic typing scheme for *Legionella pneumophila* with whole genome sequence data using validation guidelines

Sophia David, Massimo Mentasti, Rediat Tewolde, Martin Aslett, Simon R. Harris, Baharak Afshar, Anthony Underwood, Norman K. Fry, Julian Parkhill & Timothy G. Harrison

Table of Contents

Table S1. The ESGLI standard typing panel of 106 isolates of *Legionella pneumophila* sg1 from 10 European countries, comprising epidemiologically "unrelated" and "related" panels.................2

Table S2. An additional 229 clinical and environmental isolates used in the evaluation of the WGS-based methods...5

Table S3. Sequencing statistics for four isolates sequenced on the Pacific Biosciences (PacBio) RSII sequencer. 12

Table S4. Quality metrics and accession numbers for all *de novo* assemblies (derived from Illumina data) used in this study. 12

Table S5. Reference genomes used in the SNP-based analysis. 19

Table S6. Reference genomes used for the mapping of all isolates in this study and the coverage achieved. 20

Table S7. 370 *L. pneumophila* isolates used to define the total core gene content of the species.28

Table S8. Genes used in the cgMLST schemes with 50, 100, 500 or 1455 core genes......................31

Table S9. 200 "accessory" genes used in the gene presence/absence scheme...................................63

Table S10. A summary of sequencing statistics for the typing panel isolates and all isolates used in this study (excluding the two complete genomes). 68

Table S11. The number of typable loci in each isolate for each extended MLST scheme................68

Table S12. 61 untypable genes in the six extended MLST schemes and the number of affected isolates in the typing panel...75

Table S13. The mean and range of mapping coverage, number of contigs and N50 values of isolates that produce complete or incomplete profiles in the extended MLST schemes. 77

Table S14. The number of accessory genes scored as present, absent or untypable using the gene presence/absence typing method. 77

Table S15. 21 genes that were scored as untypable in one or more typing panel isolates using the gene presence/absence typing method and the number of affected typing panel isolates. ...85

Table S16. The number of differences identified between isolates from epidemiologically "related" sets in the typing panel using each of the WGS-based methods. 85

Table S17. The number of differences between isolates belonging to an additional 20 epidemiologically "related" sets, as analysed by each of the WGS-based methods. 86

Table S18. The indices of discrimination (*D*) for 53 ribosomal genes, calculated using 79 epidemiologically "unrelated" isolates from the typing panel. 87

Table S19. The indices of discrimination (*D*) for 200 accessory genes, calculated using 79 epidemiologically "unrelated" isolates from the typing panel. 88

Table S20. The indices of discrimination (*D*) for 1455 core genes, calculated using 79 epidemiologically "unrelated" isolates from the typing panel. 93

References 104

Table S1. The ESGLI standard typing panel of 106 isolates of *Legionella pneumophila* sg1 from 10 European countries, comprising epidemiologically "unrelated" and "related" panels.

EUL number	Country of origin	Isolation date	ST	Related strain	Evidence of relatedness	Accession number
**Epidemiologically "unrelated" panel (*n*=79)**						
1	Switzerland	01/02/1998	1			ERR376626
2	Switzerland	01/12/1989	2			ERR376627
3	Switzerland	01/10/1989	1			ERR376628
4	Switzerland	01/01/1991	23			ERR376721
6	Switzerland	01/01/1999	42*			ERR376631
7	Switzerland	01/05/1992	18			ERR376632
8	Switzerland	01/08/1993	23			ERR376633
13	Scotland	01/01/1983	5			ERR376646
14	Scotland	06/06/1984	5			ERR376639
16	Scotland	06/06/1984	5			ERR376641
17	Scotland	01/01/1993	7			ERR376642
18	Scotland	01/01/1994	26			ERR376643
19	Scotland	01/01/1994	9			ERR376644
20	Scotland	01/01/1995	28			ERR376645
25	France	01/01/1994	44			ERR376650
26	France	Unknown	22			ERR376651
27	France	Unknown	42			ERR376652
28	France	01/01/1994	23			ERR376722
29	France	01/01/1994	20*			ERR376654
30	France	Unknown	38			ERR376655
31	France	01/01/1994	47			ERR376656
32	France	01/01/1994	16			ERR376657
33	France	Unknown	40			ERR376658
36	Italy	01/01/1999	21			ERR332122
37	Italy	01/01/1999	1			ERR376723
38	Italy	01/01/1999	1*			ERR376663
39	Italy	01/01/1999	42			ERR376664
40	Italy	01/01/1999	12			ERR376665
41	Italy	01/01/1999	23			ERR376666
42	Italy	01/01/1999	1			ERR376667

43	Italy	01/01/1999	1			ERR376668
48	Spain	01/03/1996	48			ERR332134
49	Spain	01/02/1996	20			ERR376724
50	Spain	01/03/1996	42*			ERR376675
51	Spain	01/11/1995	1156*			ERR376676
52	Spain	01/09/1995	107*			ERR376677
53	Spain	01/05/1995	1*			ERR376725
54	Spain	01/02/1994	62			ERR376679
55	Spain	01/04/1994	1*			ERR332141
60	Greece	01/01/1992	1			ERR376685
63	Greece	01/01/1993	77*			ERR332149
66	Greece	01/01/1986	77*			ERR376728
67	Greece	01/01/1995	1			ERR376692
68	England and Wales	01/09/1995	46			ERR376693
69	England and Wales	21/11/1995	37			ERR376694
70	England and Wales	09/01/1996	47			ERR376695
71	England and Wales	10/05/1996	62			ERR332157
72	England and Wales	05/02/1996	4			ERR332158
73	England and Wales	01/04/1996	37			ERR376698
74	England and Wales	14/03/1995	29			ERR376729
75	England and Wales	09/01/1995	42			ERR376700
81	Denmark	28/03/1994	53			ERR376732
82	Denmark	29/08/1994	1			ERR376733
83	Denmark	01/02/1995	50			ERR376734
84	Denmark	03/04/1995	1			ERR376735
85	Denmark	01/05/1995	1			ERR376710
86	Denmark	01/09/1995	46			ERR332172
87	Denmark	02/10/1995	2122*			ERR376712
88	Denmark	11/10/1995	1			ERR332174
91	Denmark	01/10/1995	63			ERR376737
92	Denmark	11/06/1991	53			ERR376717
93	Denmark	19/10/1992	1			ERR332179
97	Sweden	16/06/1994	9			ERR376741
98	Sweden	01/01/1996	9			ERR376629
99	Sweden	01/01/1995	34			ERR376704
100	Sweden	01/01/1995	59			ERR376742
101	Sweden	01/01/1994	60			ERR376743
102	Sweden	01/01/1993	59			ERR376714
103	Sweden	01/01/1993	45			ERR376744
104	Sweden	01/01/1992	1*			ERR376745
105	Sweden	01/01/1991	42			ERR376746
110	Germany	01/01/1993	10			ERR376674
111	Germany	01/01/1981	25			ERR376749
114 **	Germany	27/02/1995	7			ERR376752

116	Germany	01/05/1996	42			ERR376754
117	Germany	Unknown	6			ERR376755
118	Germany	01/10/1989	36			ERR340981
119	Germany	Unknown	1			ERR376757
120	Germany	01/01/1999	42			ERR376758
**Epidemiologically "related" panel (*n*=44)**						
Subdivision I ("definitely related")						
48	Spain	01/03/1996	48		Clinical isolate from patient	ERR332134
56	Spain	01/03/1996	48	EUL 48	Clinical isolate from same patient (15 days later)	ERR376726
71	England and Wales	10/05/1996	62		Clinical isolate from patient (sputum *via* direct culture)	ERR332157
76	England and Wales	10/05/1996	62	EUL 71	Clinical isolate from same patient (isolated *via* amoebae)	ERR376701
77	England and Wales	10/05/1996	62	EUL 71	Clinical isolate from same patient (isolated from faeces)	ERR376702
73	England and Wales	01/04/1996	37		Clinical isolates from the same patient - each is a single colony picked from the isolation plate	ERR376698
78	England and Wales	01/04/1996	37	EUL 73		ERR376730
79	England and Wales	01/03/1996	37	EUL 73		ERR376731
120	Germany	01/01/1999	42		Clinical isolate from patient	ERR376758
121	Germany	01/01/1999	42	EUL 120	Duplicate of EUL 120	ERR376678
Subdivision II ("probably related")						
3	Switzerland	01/10/1989	1		Clinical isolate from patient	ERR376628
9	Switzerland	01/10/1989	1	EUL 3	Environmental isolate from water (spa-pool)	ERR376634
10	Switzerland	01/10/1989	1	EUL 3	Environmental isolate from water (spa-pool)	ERR376635
8	Switzerland	01/08/1993	23		Clinical isolate from patient	ERR376633
11	Switzerland	01/08/1993	23	EUL 8	Environmental isolate from water (rest-home)	ERR376636
12	Switzerland	01/03/1993	23	EUL 8	Environmental isolate from water (rest-home)	ERR376637
19	Scotland	01/01/1994	9		Clinical isolate from patient 1	ERR376644
22	Scotland	01/01/1994	9	EUL 19	Clinical isolate from patient 2 (same outbreak)	ERR376647
23	Scotland	01/01/1994	9	EUL 19	Clinical isolate from patient 3 (same outbreak)	ERR376648
24	Scotland	01/01/1994	9	EUL 19	Related environmental isolate	ERR332110
33	France	Unknown	40		Clinical isolate from patient	ERR376658
34	France	Unknown	40	EUL 33	Related environmental isolate	ERR376659
35	France	01/01/1996	40	EUL 33	Related environmental isolate	ERR376660
37	Italy	01/01/1999	1		Clinical isolate from patient 1	ERR376723
44	Italy	01/01/1999	1	EUL 37	Related environmental isolate	ERR376669
45	Italy	01/01/1999	72	EUL 37	Clinical isolate from patient 2	ERR376670
38	Italy	01/01/1999	1*		Clinical isolate from patient	ERR376663
46	Italy	01/01/1999	1	EUL 38	Related environmental isolate	ERR376671
40	Italy	01/01/1999	12		Clinical isolate from patient	ERR376665
47	Italy	01/01/1999	12	EUL 40	Related environmental isolate	ERR376672
54	Spain	01/02/1994	62		Clinical isolate from patient (hotel-associated)	ERR376679
57	Spain	01/01/1995	62	EUL 54	Environmental isolate from shower water of hotel	ERR376682
55	Spain	01/04/1994	1*		Clinical isolate from patient (nosocomial)	ERR332141

58	Spain	01/01/1994	1	EUL 55	Environmental isolate from shower water of hospital	ERR376683
51	Spain	01/11/1995	1156*		Clinical isolate from patient (hotel-associated)	ERR376676
59	Spain	01/01/1993	1156*	EUL 51	Environmental isolate from shower water of hotel	ERR376684
93	Denmark	19/10/1992	1		Clinical isolate from patient (hotel-associated)	ERR332179
94	Denmark	08/12/1992	1	EUL 93	Clinical isolate from a related patient	ERR376738
95	Denmark	21/01/1993	1	EUL 93	Related environmental isolate	ERR376739
81	Denmark	28/03/1994	53		Environmental isolate	ERR376732
96	Denmark	01/01/1994	53	EUL 81	Clinical isolate from patient (community-acquired)	ERR376740
97	Sweden	16/06/1994	9		Clinical isolate from patient (community-acquired)	ERR376741
106	Sweden	01/01/1994	9	EUL 97	Clinical isolate from same patient	ERR332181
107	Sweden	01/01/1994	9	EUL 97	Related environmental isolate	ERR376747

ST: sequence type

*The STs of all typing panel isolates were re-called using the latest sequence-based typing (SBT) protocol (version 5.0) and from the whole genome assemblies. While all are concordant using these two methods, those marked with an asterisk are discordant with the originally designated ST, as assigned by older SBT protocols prior to the introduction of the sequence quality tool and using less optimal primers. In most of these isolates, just one allele has changed, although in some, up to three alleles have been re-designated.
**EUL 114 was used as a substitute for EUL 112, which yielded a different ST to that recorded (both *in silico* and *via* traditional SBT).

Table S2. An additional 229 clinical and environmental isolates used in the evaluation of the WGS-based methods.

Isolate number	Country of origin	Date of isolation	ST	Sg	Related isolate	Evidence of relatedness	Accession number
**3 pairs of epidemiologically related non-sg1 isolates**							
LC 202/ EUL 153	UK	17/12/1986	68	6		Clinical isolate from patient (nosocomial)	ERR376775
LC 206/ EUL 158	UK	01/12/1986	68	6	EUL 153	Related environmental isolate	ERR376780
LC 569/ EUL 154	UK	21/05/1988	1326	8		Clinical isolate from patient (nosocomial)	ERR376776
LC 606/ EUL 155	UK	01/06/1988	1326	8	EUL 154	Related environmental isolate	ERR376777
LC 384/ EUL 156	Belgium	13/07/1987	1362	10		Clinical isolate from patient (nosocomial)	ERR376778
LC 395/ EUL 159	Belgium	01/07/1984	1362	10	EUL 156	Related environmental isolate	ERR352158
**Point-source outbreak (Barrow-in-Furness, 2002)**							
LC6379-1/ EUL 145	UK	09/08/2002	78	1		Environmental isolate from cooling tower 2 pond (recently working)	ERR376769
LC6376	UK	09/08/2002	78	1	LC6379-1/ EUL 145	Environmental isolate from cooling tower 1 pond (not recently	ERR376790

						working)	
LC6382	UK	09/08/2002	78	1	LC6379-1/ EUL 145	Environmental isolate from tower 2 water cascade (working)	ERR376792
LC6391	UK	09/08/2002	78	1	LC6379-1/ EUL 145	Environmental isolate from tower 1 water cascade (not working)	ERR376793
LC6394	UK	09/08/2002	78	1	LC6379-1/ EUL 145	Environmental isolate from tower 1 water cascade (not working) (different sample to one above)	ERR376794
LC6397	UK	12/08/2002	78	1	LC6379-1/ EUL 145	Clinical isolate from patient 1	ERR376795
LC6406	UK	01/08/2002	78	1	LC6379-1/ EUL 145	Clinical isolate from patient 2	ERR376796
LC6407	UK	15/08/2002	78	1	LC6379-1/ EUL 145	Clinical isolate from patient 3	ERR376797
LC6408	UK	12/08/2002	78	1	LC6379-1/ EUL 145	Clinical isolate from patient 4	ERR341023
LC6411	UK	01/08/2002	78	1	LC6379-1/ EUL 145	Clinical isolate from patient 5	ERR376799
LC6412	UK	15/08/2002	78	1	LC6379-1/ EUL 145	Clinical isolate from patient 6	ERR376800
LC6413	UK	15/08/2002	78	1	LC6379-1/ EUL 145	Clinical isolate from patient 7	ERR376801
LC6416	UK	01/08/2002	78	1	LC6379-1/ EUL 145	Clinical isolate from patient 8	ERR376802
LC6418	UK	15/08/2002	78	1	LC6379-1/ EUL 145	Clinical isolate from patient 9	ERR376804
LC6385	UK	09/08/2002	78	1	LC6379-1/ EUL 145	Environmental isolate from tower 2 water cascade (working)	ERR352162
LC6388	UK	09/08/2002	78	1	LC6379-1/ EUL 145	Environmental isolate from tower 2 water cascade (working)	ERR352163
LC6409	UK	15/08/2002	78	1	LC6379-1/ EUL 145	Clinical isolate from patient 10	ERR352164
LC6410	UK	15/08/2002	78	1	LC6379-1/ EUL 145	Clinical isolate from patient 11	ERR352165
**Point-source outbreak (BBC, Portland Place, 1988)**							
LC0537/ EUL 132	UK	01/05/1988	37	1		Clinical isolate from patient 1	ERR332168
LC0539/ EUL 133	UK	01/05/1988	37	1	LC0537/ EUL 132	Clinical isolate from patient 2	ERR332169
LC0540/ EUL 134	UK	01/05/1998	37	1	LC0537/ EUL 132	Clinical isolate from patient 3	ERR332170
LC0565	UK	01/05/1988	37	1	LC0537/ EUL 132	Clinical isolate from patient 4	ERR363880
LC0583	UK	01/05/1988	37	1	LC0537/ EUL 132	Clinical isolate from patient 5	ERR363881
**Point-source outbreak (Hereford, 2003)**							
H034680033	UK	01/11/2003	37	1		Clinical isolate from patient 1	ERR1232479
H034680035/ EUL 165	UK	01/11/2003	37	1	H034680033	Clinical isolate from patient 2	ERR376785
H034690056/ EUL 166	UK	01/11/2003	37	1	H034680033	Environmental isolate from site A cooling tower 1	ERR376786
H034800427	UK	01/11/2003	37	1	H034680033	Environmental isolate from site A cooling tower 2	ERR1232480
H034980467	UK	01/11/2003	37	1	H034680033	Environmental isolate from domestic spa pool	ERR1232481
**Additional ST1 isolates**							
Paris	France	Unknown	1	1			Cazalet *et al.*

							(2004)
H034800423	UK	01/11/2003	1	1			Reuter *et al.* (2013)
OLDA1 (NCTC12008)	USA	01/01/1947	1	1			ERR434061
EUL 109	Sweden	01/01/1992	1	1			ERR376662
**Additional ST37 isolates**							
H064240448	UK	12/10/2006	37	1			ERR363849
LC0731	UK	01/02/1989	37	1		Clinical isolate from patient 1	ERR363882
LC0732	UK	01/02/1989	37	1	LC0731	Clinical isolate from patient 2	ERR363883
LC0763	UK	01/02/1989	37	1	LC0731	Related environmental isolate	ERR363884
LC0782	UK	01/02/1989	37	1	LC0731	Clinical isolate from patient 3	ERR363885
LC0795	UK	01/02/1989	37	1	LC0731	Clinical isolate from patient 4	ERR363887
LC0801	UK	01/02/1989	37	1	LC0731	Clinical isolate from patient 5	ERR363889
LC5694	UK	12/07/2000	37	1			ERR363891
LC5722	UK	31/08/2000	37	1			ERR363892
LC5738	UK	05/10/2000	37	1			ERR363893
LC5755	UK	01/11/2000	37	1			ERR363894
LC6163	UK	15/02/2002	37	1			ERR363897
LC6267	UK	10/07/2002	37	1			ERR363899
LC6268	UK	05/07/2002	37	1			ERR363900
LC6228	UK	10/04/2002	37	1			ERR363898
H041380048	UK	30/04/2004	37	1		Clinical isolate from patient	ERR363843
H041640791	UK	12/04/2004	37	1	H041380048	Related environmental isolate	ERR363844
H042960010	UK	10/08/2004	37	1			ERR363845
H061140013	UK	19/04/2006	37	1			ERR363847
H071880001	UK	08/06/2007	37	1			ERR363850
H073060003	UK	30/08/2007	37	1			ERR363851
H080820009	UK	15/03/2008	37	1			ERR363853
LC6058	Unknown (travel-associated)	19/10/2001	37	1			ERR363896
LC6293	Unknown (travel-associated)	24/07/2002	37	1			ERR363901
LC6788	Unknown (travel-associated)	30/07/2003	37	1			ERR363902
H062660463	Unknown (travel-associated)	03/07/2006	37	1			ERR363848
H073900557	Unknown (travel-associated)	21/09/2007	37	1			ERR363852
LC1127	UK	26/12/1989	37	1			ERR363890
H084760449	UK	17/11/2008	37	1			ERR363857
H085020185	UK	15/12/2008	37	1			ERR363858
H090320386	UK	12/01/2009	37	1			ERR363859
H044260061	UK	11/10/2004	37	1			ERR363846
H093140322	UK	01/07/2009	37	1		Clinical isolate from patient	ERR363861
H093160422	UK	17/07/2009	37	1	H093140322	Related environmental	ERR363862

						isolate	
H092760433	Unknown (travel-associated)	06/07/2009	37	1			ERR363860
H100940111	UK	08/03/2010	37	1			ERR363863
H101760092	UK	03/05/2010	37	1			ERR363864
H101820190	UK	11/05/2010	37	1			ERR363865
H102020414	UK	24/05/2010	37	1			ERR363867
H101980130	Unknown (travel-associated)	17/05/2010	37	1			ERR363866
H103820081	UK	24/09/2010	37	1			ERR363868
H120240685	Slovenia	15/09/2010	37	1			ERR363992
H104320293	UK	26/10/2010	37	1			ERR363869
H113180118	UK	01/08/2011	37	1		Clinical isolate from patient	ERR363871
H113340664	UK	05/08/2011	37	1	H113180118	Related environmental isolate	ERR363873
H113280076	UK	05/08/2011	37	1			ERR363872
H113660550	UK	12/09/2011	37	1			ERR363874
H114740454	UK	20/11/2011	37	1			ERR363876
H115040456	UK	11/12/2011	37	1			ERR363877
H111580389	UK	18/04/2011	37	1			ERR363870
H113780240	Unknown (travel-associated)	19/08/2011	37	1			ERR363875
H083920177	UK	26/09/2008	37	1		Clinical isolate from patient	ERR363855
H084140691	UK	03/10/2008	37	1	H083920177	Related environmental isolate	ERR363856
H081180019	UK	11/03/2008	37	1			ERR363854
H103260667	Greece	16/08/2010	37	1			ERR363938
LC464	UK	01/11/1987	37	1			ERR363878
LC0512	Unknown (travel-associated)	01/01/1988	37	1			ERR363879
LC0794	UK	01/02/1989	37	1		Clinical isolate from patient 1	ERR363886
LC0798	UK	01/02/1989	37	1	LC0794	Clinical isolate from patient 2	ERR363888
LC0536/EUL 131	UK	01/05/1988	37*	1			ERR332167
**Additional ST42 isolates**							
LC230/ EUL 122	Unknown	01/03/1987	42	1		Clinical isolate from patient, isolated *via* direct plating	ERR376759
LC231/ EUL 123	Unknown	01/03/1987	42	1	LC230/ EUL 122	Isolate from same patient, isolated *via* amoebal enrichment	ERR332142
LC0462/ EUL 124	UK	01/11/1987	42	1		Clinical isolate from patient, isolated via direct plating	ERR332150
LC0463/ EUL 125	UK	01/11/1987	42	1	LC0462/ EUL 124	Isolate from same patient, isolated *via* amoebal enrichment	ERR376760
**Additional ST47 isolates**							
Lorraine	France	20/08/2004	47	1			Gomez-Valero *et al.* (2011)
H063920004/ EUL 169	UK	25/09/2006	47	1		Clinical isolate from patient	Underwood *et al.* (2013)
H064160534/	UK	10/10/2006	47	1	H063920004	Environmental isolate	ERR363994

EULV0410					/EUL 169	from swimming pool	
H064160538/ EUL 170	UK	10/10/2006	47	1	H063920004 /EUL 169	Environmental isolate from spa pool (attached to swimming pool)	ERR376788
H034700617	UK	20/11/2003	47	1			Reuter *et al.* (2013)
H043580159	UK	01/09/2004	47	1			ERR363943
H043580160	UK	01/09/2004	47	1			ERR363959
H043660021	UK	01/09/2004	47	1			ERR363946
H043680663	UK	01/09/2004	47	1			ERR363949
H043700021	UK	01/09/2004	47	1			ERR363944
H043790008	UK	01/09/2004	47	1			ERR363945
H052920051	UK	01/07/2005	47	1			ERR363961
H053540106	UK	01/08/2005	47	1			ERR363948
H063660005	UK	01/09/2006	47	1		Clinical isolate from patient 1	ERR363904
H063660006	UK	09/09/2006	47	1	H063660005	Clinical isolate from patient 2, clustered in time and space with patient 1	ERR363922
H063760006	UK	14/09/2006	47	1	H063660005	Clinical isolate from patient 3, clustered in time and space with patient 1	ERR363915
H063660009	UK	01/09/2006	47	1			ERR363911
H063680006	UK	10/09/2006	47	1		Clinical isolate from patient 1	ERR363918
H063680007	UK	10/09/2006	47	1	H063680006	Clinical isolate from patient 2, clustered in time and space with patient 1	ERR363913
H063740003	UK	01/09/2006	47	1			ERR363929
H063740018	UK	01/09/2006	47	1			ERR363906
H063780007	UK	01/09/2006	47	1		Clinical isolate from patient 1	ERR363934
H063780008	UK	01/09/2006	47	1	H063780007	Clinical isolate from patient 2, clustered in time and space with patient 1	ERR363916
H063860003	UK	21/09/2006	47	1			ERR363930
H063960001	UK	01/09/2006	47	1			ERR363928
LC5759	Unknown (travel-associated)	23/10/2000	47	1			ERR363995
H070420013	UK	26/02/2007	47	1			ERR363968
LC5822	UK	07/02/2001	47	1			ERR363996
H040260015	UK	10/02/2004	47	1			ERR363903
H055140095	UK	15/01/2006	47	1			ERR363947
H060780053	UK	12/03/2006	47	1			ERR363907
H061120064	UK	10/04/2006	47	1			ERR363914
H062840608	UK	15/08/2006	47	1			ERR363917
H062940111	UK	22/08/2006	47	1			ERR363919
H064320006	UK	20/11/2006	47	1			ERR363923
H064280005	UK	24/11/2006	47	1			ERR363924
H064380002	UK	22/11/2006	47	1			ERR363926
H064380001	UK	30/11/2006	47	1			ERR363921
H064560527	UK	12/12/2006	47	1			ERR363925
H064660638	UK	20/12/2006	47	1			ERR363964

H070160015	UK	07/02/2007	47	1			ERR363970
H071120010	UK	16/04/2007	47	1			ERR363931
H071360036	UK	02/05/2007	47	1			ERR363908
H072740002	UK	08/08/2007	47	1			ERR363935
H073000045	UK	26/08/2007	47	1			ERR363932
H073380007	UK	13/09/2007	47	1			ERR363940
H073600182	UK	06/10/2007	47	1			ERR363976
H073640185	UK	09/10/2007	47	1			ERR363933
H074960018	UK	02/01/2008	47	1			ERR363920
H080780059	UK	13/03/2008	47	1			ERR363910
H053840008	UK	01/10/2004	47	1			ERR363954
H072520002	UK	22/06/2007	47	1			ERR363927
H081340222	UK	29/03/2007	47	1			ERR363909
H082520613	UK	20/06/2008	47	1			ERR363912
H083120262	UK	01/08/2008	47	1			ERR363941
H083620580	UK	05/09/2008	47	1			ERR363936
H083960064	UK	29/09/2008	47	1			ERR363937
H084620118	UK	17/11/2008	47	1			ERR363939
H090140214	UK	05/01/2009	47	1			ERR363963
H090440226	UK	26/01/2009	47	1			ERR363966
H040960441	UK	19/02/2004	47	1			ERR363953
H041120007	UK	05/03/2004	47	1			ERR363942
H093480403	Unknown (travel-associated)	24/08/2009	47	1			ERR363973
H094340202	UK	26/10/2009	47	1			ERR363971
H095060125	UK	14/12/2009	47	1			ERR363972
H100140151	UK	18/01/2010	47	1			ERR363965
H100660110	UK	15/02/2010	47	1			ERR363962
H100700025	UK	19/02/2010	47	1			ERR363958
H103140121	UK	02/08/2010	47	1			ERR363967
H103620160	UK	10/09/2010	47	1			ERR363950
H103660126	UK	23/09/2010	47	1			ERR363974
H103660121	UK	11/09/2010	47	1			ERR363956
H104420240	UK	04/11/2010	47	1			ERR363957
H110480273	UK	03/01/2011	47	1			ERR363969
H112320437	UK	06/06/2011	47	1			ERR363951
H112080616	UK	23/05/2011	47	1			ERR363952
H112380374	UK	13/06/2011	47	1			ERR363960
H120160499	UK	09/01/2012	47	1			ERR363985
H120200371	UK	12/01/2012	47	1			ERR363984
H105140391	UK	28/12/2010	47	1			ERR363993
H121040204	UK	09/03/2012	47	1			ERR363982
H121420445	UK	03/04/2012	47	1			ERR363983
H102240357	UK	19/05/2010	47	1			ERR363955
H122500497	UK	21/06/2012	47	1			ERR363981
H122820408	Unknown (travel-associated)	06/07/2012	47	1			ERR363980
H123620597	UK	04/09/2012	47	1			ERR363979
H123840629	UK	21/09/2012	47	1			ERR363978
H123940534	UK	28/09/2012	47	1			ERR363975

H124920387	UK	06/12/2012	47	1			ERR363991
H131340777	UK	29/03/2013	47	1		Clinical isolate from patient	ERR363990
H131480353	UK	01/04/2013	47	1	H131340777	Related environmental isolate	ERR363989
H131480354	UK	01/04/2013	47	1	H131340777	Related environmental isolate	ERR363988
H131840211	UK	01/04/2013	47	1	H131340777	Related environmental isolate	ERR363986
H131460248	UK	06/04/2013	47	1			ERR363987
H132140863	UK	24/05/2013	47	1			ERR364031
H053640534/ EUL 168	UK	01/09/2005	47	1			ERR352161
**Additional ST62 isolates**							
H064180002	UK	01/10/2006	62	1		Clinical isolate from patient	Underwood *et al.* (2013)
H064180019	UK	09/10/2006	62	1	H064180002	Related environmental isolate	ERR364004
H043540106	Unknown (travel-associated)	01/08/2004	62	1			ERR363997
H044120014	Bulgaria	01/10/2004	62	1			ERR363999
H052780022	UK	01/07/2005	62	1			ERR363998
H054280040	UK	01/11/2005	62	1			ERR364028
H063680003	UK	01/09/2006	62	1			ERR364002
H063840008	UK	04/09/2006	62	1			ERR364001
H073660582	UK	01/09/2007	62	1			ERR364008
LC5804	UK	01/11/2000	62	1			ERR364029
H063760005	UK	10/10/2006	62	1			ERR364000
H064240003	UK	14/11/2006	62	1			ERR364005
H065040012	UK	07/01/2007	62	1			ERR364012
H070140635	UK	06/02/2007	62	1			ERR364011
H073020039	UK	28/08/2007	62	1			ERR364022
H073320399	UK	10/09/2007	62	1			ERR364010
H073440003	UK	18/09/2007	62	1			ERR364009
LC6009	Unknown (travel-associated)	26/07/2001	62	1			ERR364030
H083140015	UK	25/07/2008	62	1			ERR364007
H093400182	UK	31/07/2009	62	1			ERR364006
H094760070	UK	23/11/2009	62	1			ERR364003
H094800237	UK	26/11/2009	62	1			ERR364020
H110480715	UK	21/01/2011	62	1			ERR364018
H112840293	UK	13/07/2011	62	1			ERR364017
H114100406	Greece	13/10/2011	62	1			ERR364016
H120240362	UK	16/01/2012	62	1			ERR364025
H104640262	Unknown (travel-associated)	19/11/2010	62	1			ERR364019
H123140428	UK	31/07/2012	62	1			ERR364015
H123460520	UK	27/08/2012	62	1			ERR364014
H124360642	UK	27/10/2012	62	1			ERR364013
Pontiac-1	USA	01/07/1968	62	1			ERR1232478

ST: sequence type
Sg: serogroup

*The ST of LC0536/EUL 131 has been re-designated as 37 (from 13), as determined using the latest sequence-based typing (SBT) protocol (v. 5.0) and using the whole genome assembly.

Table S3. Sequencing statistics for four isolates sequenced on the Pacific Biosciences (PacBio) RSII sequencer.

**Isolate**	**No. SMRT cells**	**No. of contigs**	**Extra-chromosomal plasmid?**	**Mean coverage**	**Total mapped reads**	**Mapped subread N50**	**Accession numbers**
EUL 28 (ST23)	2	2 (3514605bp and 149271bp)	Yes	79.3x	67032	2.97kb	ERR660551 ERR663930
EUL 120 (ST42)	4	2 (2732926bp) and 697556bp)	No	121.8x	100833	3.46kb	ERR663926 ERR663929 ERR671908 ERR690961
EUL 165 (ST37)	3	1 (3486389bp)	No	101.3x	74378	3.47kb	ERR663927 ERR676880 ERR676882
H044120014 (ST62)	2	1 (3541412bp)	No	62x	45460	4.59kb	ERR663928 ERR676881

Table S4. Quality metrics and accession numbers for all *de novo* assemblies (derived from Illumina data) used in this study.

**EUL/isolate number**	**Assembly length (bp)**	**No. of contigs**	**N50 (bp)**	**Accession number**
**Typing panel**				
1	3582272	43	221291	FJAR01000001-FJAR01000043
2	3467814	21	441390	FJAF01000001-FJAF01000021
3	3584140	42	168231	FJAN01000001-FJAN01000042
4	3682698	37	180190	FJBD01000001-FJBD01000037
6	3387307	32	248780	FJBM01000001-FJBM01000032
7	3516217	36	198712	FJAI01000001-FJAI01000036
8	3489430	37	188012	FJBU01000001-FJBU01000037
13	3606063	38	221291	FJBF01000001-FJBF01000038
14	3606338	42	168264	FJAG01000001-FJAG01000042
16	3605510	44	168255	FJBH01000001-FJBH01000044
17	3440178	39	219509	FJBJ01000001-FJBJ01000039
18	3229839	38	143054	FJAW01000001-FJAW01000038
19	3422384	46	197885	FJAL01000001-FJAL01000046
20	3348748	18	336934	FJBO01000001-FJBO01000018
25	3353388	19	298032	FJAO01000001-FJAO01000019
26	3330854	36	164985	FJAB01000001-FJAB01000036
27	3493198	24	250279	FJAY01000001-FJAY01000024

27 (replicate)	3493923	30	250250	FJNG01000001-FJNG01000030
28	3624059	37	204558	FJBP01000001-FJBP01000037
29	3547570	32	199980	FJBR01000001-FJBR01000032
30	3295143	19	401585	FJAE01000001-FJAE01000019
31	3541152	62	103738	FJAT01000001-FJAT01000062
32	3545771	38	242920	FJAD01000001-FJAD01000038
33	3294148	23	333325	FJAH01000001-FJAH01000023
33 (replicate)	3294679	23	338768	FJNK01000001-FJNK01000023
36	3507348	36	200628	FJAM01000001-FJAM01000036
37	3446273	42	235781	FJBN01000001-FJBN01000042
38	3570332	33	235792	FJBQ01000001-FJBQ01000033
39	3347795	20	264182	FJAU01000001-FJAU01000020
40	3438629	22	275955	FJAV01000001-FJAV01000022
41	3488391	34	188086	FJBW01000001-FJBW01000034
42	3581764	43	168249	FJAP01000001-FJAP01000043
43	3575213	37	168244	FJBL01000001-FJBL01000037
48	3503195	65	101284	FJBT01000001-FJBT01000065
49	3517591	57	237958	FJBE01000001-FJBE01000057
50	3385882	24	214368	FJBG01000001-FJBG01000024
51	3405363	15	726453	FJBB01000001-FJBB01000015
52	3318517	28	639347	FJBX01000001-FJBX01000028
53	3578730	41	168217	FJBY01000001-FJBY01000041
54	3453348	34	183458	FJBZ01000001-FJBZ01000034
55	3579917	37	168229	FJCA01000001-FJCA01000037
60	3583463	47	167804	FJCF01000001-FJCF01000047
63	3461713	24	281315	FJCG01000001-FJCG01000024
66	3461261	30	263172	FJCH01000001-FJCH01000030
67	3580420	38	168281	FJCI01000001-FJCI01000038
68	3353004	64	86373	FJCJ01000001-FJCJ01000064
69	3348891	19	525181	FJCK01000001-FJCK01000019
69 (replicate)	3348937	22	357199	FJNJ01000001-FJNJ01000022
70	3598684	68	103511	FJCL01000001-FJCL01000068
71	3485244	38	196616	FJCM01000001-FJCM01000038
72	3346824	19	437858	FJCN01000001-FJCN01000019
73	3349062	17	336718	FJCP01000001-FJCP01000017
74	3459400	27	713253	FJCQ01000001-FJCQ01000027
75	3386255	27	196971	FJCO01000001-FJCO01000027
75 (replicate)	3385427	26	214349	FJNI01000001-FJNI01000026
81	3514412	36	223875	FJCV01000001-FJCV01000036
82	3576637	38	262463	FJCX01000001-FJCX01000038
83	3515373	40	228374	FJCW01000001-FJCW01000040
84	3446240	36	263208	FJCY01000001-FJCY01000036
85	3577453	33	262466	FJCZ01000001-FJCZ01000033
86	3547658	72	101986	FJDC01000001-FJDC01000072
87	3422196	20	246513	FJDB01000001-FJDB01000020
88	3576785	34	262392	FJDA01000001-FJDA01000034
91	3266471	34	223981	FJDD01000001-FJDD01000034
92	3514819	42	216313	FJDE01000001-FJDE01000042
92 (replicate)	3513289	39	225203	FJNL01000001-FJNL01000039
93	3641343	43	262032	FJDF01000001-FJDF01000043

97	3465455	41	250633	FJDJ01000001-FJDJ01000041
98	3465680	42	248414	FJDK01000001-FJDK01000042
99	3259617	19	657238	FJDL01000001-FJDL01000019
100	3351397	34	183216	FJDO01000001-FJDO01000034
101	3429712	48	151004	FJDM01000001-FJDM01000048
102	3369225	39	176020	FJDN01000001-FJDN01000039
103	3669856	70	103529	FJDP01000001-FJDP01000070
104	3607517	47	155184	FJDQ01000001-FJDQ01000047
105	3383263	21	445776	FJDR01000001-FJDR01000021
110	3624710	43	168239	FJDU01000001-FJDU01000043
111	3298695	41	124718	FJDW01000001-FJDW01000041
111 (replicate)	3299082	47	126247	FJNH01000001-FJNH01000047
114	3440457	40	221541	FJDV01000001-FJDV01000040
116	3300129	16	444178	FJDX01000001-FJDX01000016
117	3437615	47	121106	FJDY01000001-FJDY01000047
118	3410805	25	356081	FJDZ01000001-FJDZ01000025
119	3571941	36	220433	FJEA01000001-FJEA01000036
120	3384999	21	264302	FJEB01000001-FJEB01000021
9	3583104	44	168236	FJBC01000001-FJBC01000044
10	3583674	37	262030	FJAZ01000001-FJAZ01000037
11	3488698	31	242931	FJBI01000001-FJBI01000031
12	3487861	31	215445	FJAJ01000001-FJAJ01000031
22	3436716	19	320105	FJBS01000001-FJBS01000019
23	3438368	21	336944	FJAC01000001-FJAC01000021
24	3436497	24	337270	FJAS01000001-FJAS01000024
34	3294395	21	334734	FJAX01000001-FJAX01000021
35	3294934	18	336503	FJBK01000001-FJBK01000018
44	3580987	41	235794	FJBV01000001-FJBV01000041
45	3453726	40	235793	FJBA01000001-FJBA01000040
46	3570783	37	168238	FJAQ01000001-FJAQ01000037
47	3438617	26	275143	FJAK01000001-FJAK01000026
56	3499528	63	103579	FJCC01000001-FJCC01000063
57	3452138	34	183315	FJCB01000001-FJCB01000034
58	3581148	43	169426	FJCE01000001-FJCE01000043
59	3406404	20	244116	FJCD01000001-FJCD01000020
76	3485883	39	162984	FJCR01000001-FJCR01000039
77	3486431	36	164472	FJCS01000001-FJCS01000036
78	3348065	25	324151	FJCT01000001-FJCT01000025
79	3349415	28	336667	FJCU01000001-FJCU01000028
94	3640935	37	262076	FJDH01000001-FJDH01000037
95	3641028	37	262384	FJDG01000001-FJDG01000037
96	3513757	37	223869	FJDI01000001-FJDI01000037
106	3464579	35	250633	FJDS01000001-FJDS01000035
107	3464340	39	249913	FJDT01000001-FJDT01000039
121	3385179	21	300906	FJEC01000001-FJEC01000021
**Additional isolates**				
LC 202/EUL 153	3370172	21	332679	FJED01000001-FJED01000021
LC 206/EUL 158	3369964	17	485867	FJEF01000001-FJEF01000017
LC 569/EUL 154	3416299	12	2134649	FJEG01000001-FJEG01000012
LC 606/EUL 155	3416417	12	1881974	FJEE01000001-FJEE01000012

LC 384/EUL 156	3487522	22	403021	FJEH01000001-FJEH01000022
LC 395/EUL 159	3482177	20	413838	FJEI01000001-FJEI01000020
LC6379-1/EUL 145	3365082	30	184547	FJEK01000001-FJEK01000030
LC6376	3365099	30	391540	FJEJ01000001-FJEJ01000030
LC6382	3364319	27	335444	FJEL01000001-FJEL01000027
LC6391	3363339	27	243038	FJEN01000001-FJEN01000027
LC6394	3364625	32	184559	FJEM01000001-FJEM01000032
LC6397	3363172	29	184544	FJEQ01000001-FJEQ01000029
LC6406	3363398	30	242960	FJEO01000001-FJEO01000030
LC6407	3362234	29	242960	FJEP01000001-FJEP01000029
LC6408	3362492	35	242960	FJER01000001-FJER01000035
LC6411	3363467	26	251479	FJEU01000001-FJEU01000026
LC6412	3362944	34	184542	FJES01000001-FJES01000034
LC6413	3362923	32	184530	FJET01000001-FJET01000032
LC6416	3362654	30	242960	FJEV01000001-FJEV01000030
LC6418	3362330	30	184783	FJEW01000001-FJEW01000030
LC6385	3364576	27	242794	FJEX01000001-FJEX01000027
LC6388	3363943	27	242791	FJEY01000001-FJEY01000027
LC6409	3364426	28	336094	FJEZ01000001-FJEZ01000028
LC6410	3365323	25	251329	FJFA01000001-FJFA01000025
LC0537/EUL 132	3412033	24	324159	FJFB01000001-FJFB01000024
LC0539/EUL 133	3413672	25	638011	FJFC01000001-FJFC01000025
LC0540/EUL 134	3412957	27	327654	FJFD01000001-FJFD01000027
LC0565	3413362	24	324339	FJFE01000001-FJFE01000024
LC0583	3414048	26	324530	FJFF01000001-FJFF01000026
H034680033	3446924	30	294935	FJOB01000001-FJOB01000030
H034680035/EUL 165	3444436	25	336672	FJFG01000001-FJFG01000025
H034690056/EUL 166	3445871	22	739607	FJFH01000001-FJFH01000022
H034800427	3446329	20	409792	FJNZ01000001-FJNZ01000020
H034980467	3446486	17	584207	FJNY01000001-FJNY01000017
H034800423	3557791	48	129964	FJOE01000001-FJOE01000323
OLDA1 (NCTC12008)	3586509	42	241020	FJFJ01000001-FJFJ01000042
EUL 109	3609634	42	155185	FJFI01000001-FJFI01000042
H064240448	3412426	18	788251	FJFK01000001-FJFK01000018
LC0731	3389456	23	356594	FJFM01000001-FJFM01000023
LC0732	3388952	20	435223	FJFL01000001-FJFL01000020
LC0763	3388509	19	337610	FJFN01000001-FJFN01000019
LC0782	3388588	22	474242	FJHM01000001-FJHM01000022
LC0795	3388538	22	434228	FJHO01000001-FJHO01000022
LC0801	3389651	20	337731	FJHR01000001-FJHR01000020
LC5694	3340104	20	308508	FJFO01000001-FJFO01000020
LC5722	3339668	27	304117	FJFP01000001-FJFP01000027
LC5738	3339758	22	248375	FJFQ01000001-FJFQ01000022
LC5755	3415172	26	336687	FJFR01000001-FJFR01000026
LC6163	3350195	23	248366	FJFS01000001-FJFS01000023
LC6267	3413801	24	324534	FJFT01000001-FJFT01000024
LC6268	3413910	22	414101	FJFU01000001-FJFU01000022
LC6228	3446506	21	324341	FJFV01000001-FJFV01000021
H041380048	3411003	22	336628	FJFW01000001-FJFW01000022
H041640791	3411081	20	336634	FJGD01000001-FJGD01000020

H042960010	3411388	19	417334	FJFX01000001-FJFX01000019
H061140013	3414012	22	337363	FJFY01000001-FJFY01000022
H071880001	3351157	26	317885	FJFZ01000001-FJFZ01000026
H073060003	3413611	23	336671	FJGB01000001-FJGB01000023
H080820009	3581505	27	656985	FJGA01000001-FJGA01000027
LC6058	3412804	25	549630	FJGC01000001-FJGC01000025
LC6293	3414379	22	1076411	FJGE01000001-FJGE01000022
LC6788	3443537	20	549813	FJGF01000001-FJGF01000020
H062660463	3351247	23	330279	FJGG01000001-FJGG01000023
H073900557	3494788	27	324154	FJGH01000001-FJGH01000027
LC1127	3389579	23	337256	FJGJ01000001-FJGJ01000023
H084760449	3445602	22	324339	FJGI01000001-FJGI01000022
H085020185	3446838	18	770488	FJGK01000001-FJGK01000018
H090320386	3409856	24	336675	FJGL01000001-FJGL01000024
H044260061	3445323	22	969495	FJGM01000001-FJGM01000022
H093140322	3445924	25	351298	FJGN01000001-FJGN01000025
H093160422	3445791	21	548795	FJGO01000001-FJGO01000021
H092760433	3461547	34	304114	FJGP01000001-FJGP01000034
H100940111	3411026	27	248357	FJGQ01000001-FJGQ01000027
H101760092	3350043	23	324616	FJGR01000001-FJGR01000023
H101820190	3411782	19	416839	FJGS01000001-FJGS01000019
H102020414	3445616	26	336678	FJGT01000001-FJGT01000026
H101980130	3351235	25	311133	FJGU01000001-FJGU01000025
H103820081	3482669	31	219507	FJGV01000001-FJGV01000031
H120240685	3431770	27	330426	FJGW01000001-FJGW01000027
H104320293	3413966	29	355130	FJGY01000001-FJGY01000029
H113180118	3341309	22	301691	FJGX01000001-FJGX01000022
H113340664	3341936	20	388532	FJHA01000001-FJHA01000020
H113280076	3380585	20	590371	FJGZ01000001-FJGZ01000020
H113660550	3423689	20	509386	FJHB01000001-FJHB01000020
H114740454	3413960	24	336679	FJHC01000001-FJHC01000024
H115040456	3413149	26	336680	FJHD01000001-FJHD01000026
H111580389	3446413	20	484400	FJHE01000001-FJHE01000020
H113780240	3414602	25	482710	FJHF01000001-FJHF01000025
H083920177	3441071	22	304113	FJHG01000001-FJHG01000022
H084140691	3410070	21	416599	FJHH01000001-FJHH01000021
H081180019	3348323	25	249077	FJHI01000001-FJHI01000025
H103260667	3453245	18	498080	FJHJ01000001-FJHJ01000018
LC464	3350265	22	324172	FJHK01000001-FJHK01000022
LC0512	3413673	22	638021	FJHL01000001-FJHL01000022
LC0794	3413957	26	324532	FJHN01000001-FJHN01000026
LC0798	3415031	24	364734	FJHP01000001-FJHP01000024
LC0536/EUL 131	3422513	35	468672	FJHQ01000001-FJHQ01000035
LC230/EUL 122	3443140	31	259364	FJHS01000001-FJHS01000031
LC231/EUL 123	3442071	28	264302	FJHU01000001-FJHU01000028
LC0462/EUL 124	3386214	18	323630	FJHT01000001-FJHT01000018
LC0463/EUL 125	3385613	28	304205	FJHV01000001-FJHV01000028
H063920004/EUL 169	3540658	65	104090	FJHW01000001-FJHW01000065
H064160534/EULV0410	3542236	61	103515	FJHY01000001-FJHY01000061
H064160538/EUL 170	3542584	66	103231	FJLL01000001-FJLL01000066

H034700617	3535096	74	82988	FJOC01000001-FJOC01000074
H043580159	3540112	63	102126	FJHX01000001-FJHX01000063
H043580160	3543495	68	102118	FJHZ01000001-FJHZ01000068
H043660021	3539680	58	86360	FJIB01000001-FJIB01000058
H043680663	3542231	60	103521	FJIC01000001-FJIC01000060
H043700021	3572737	63	94275	FJIA01000001-FJIA01000063
H043790008	3540216	58	103518	FJIE01000001-FJIE01000058
H052920051	3538542	57	104627	FJID01000001-FJID01000057
H053540106	3541070	64	94274	FJIF01000001-FJIF01000064
H063660005	3540882	65	94015	FJIG01000001-FJIG01000065
H063660006	3540846	62	94015	FJIH01000001-FJIH01000062
H063760006	3542859	72	81300	FJIN01000001-FJIN01000072
H063660009	3540749	61	103554	FJII01000001-FJII01000061
H063680006	3595917	73	81272	FJIJ01000001-FJIJ01000073
H063680007	3539075	66	81274	FJIK01000001-FJIK01000066
H063740003	3538993	62	94742	FJIL01000001-FJIL01000062
H063740018	3540596	59	103556	FJIM01000001-FJIM01000059
H063780007	3540451	63	102072	FJIO01000001-FJIO01000063
H063780008	3544143	62	104160	FJIP01000001-FJIP01000062
H063860003	3526491	140	103528	FJIR01000001-FJIR01000140
H063960001	3538795	69	103540	FJIQ01000001-FJIQ01000069
LC5759	3537671	59	102058	FJIS01000001-FJIS01000059
H070420013	3541477	60	104720	FJIT01000001-FJIT01000060
LC5822	3540057	62	102059	FJIU01000001-FJIU01000062
H040260015	3541261	61	94015	FJIW01000001-FJIW01000061
H055140095	3539663	58	113585	FJIV01000001-FJIV01000058
H060780053	3541600	61	103528	FJIY01000001-FJIY01000061
H061120064	3542809	63	113625	FJIX01000001-FJIX01000063
H062840608	3540451	63	110289	FJIZ01000001-FJIZ01000063
H062940111	3538952	60	102071	FJJA01000001-FJJA01000060
H064320006	3538778	60	103523	FJJB01000001-FJJB01000060
H064280005	3540049	64	93845	FJJC01000001-FJJC01000064
H064380002	3541557	63	113617	FJJD01000001-FJJD01000063
H064380001	3540188	61	103511	FJJE01000001-FJJE01000061
H064560527	3539696	65	103511	FJJF01000001-FJJF01000065
H064660638	3541541	59	110131	FJJG01000001-FJJG01000059
H070160015	3541486	63	102059	FJJH01000001-FJJH01000063
H071120010	3540618	62	103558	FJJI01000001-FJJI01000062
H071360036	3541845	61	103511	FJJJ01000001-FJJJ01000061
H072740002	3540414	61	103550	FJJK01000001-FJJK01000061
H073000045	3538163	59	103505	FJJL01000001-FJJL01000059
H073380007	3540390	58	103525	FJJM01000001-FJJM01000058
H073600182	3542113	63	103521	FJJO01000001-FJJO01000063
H073640185	3540979	65	103550	FJJN01000001-FJJN01000065
H074960018	3540833	62	99193	FJJP01000001-FJJP01000062
H080780059	3540582	61	103556	FJJR01000001-FJJR01000061
H053840008	3541342	56	103521	FJJQ01000001-FJJQ01000056
H072520002	3541042	65	94271	FJJS01000001-FJJS01000065
H081340222	3541677	62	93140	FJJU01000001-FJJU01000062
H082520613	3541454	62	103525	FJJT01000001-FJJT01000062

H083120262	3540437	64	103519	FJJV01000001-FJJV01000064
H083620580	3540741	62	113625	FJJW01000001-FJJW01000062
H083960064	3539747	60	94015	FJJX01000001-FJJX01000060
H084620118	3537979	60	102059	FJJY01000001-FJJY01000060
H090140214	3541703	60	113601	FJJZ01000001-FJJZ01000060
H090440226	3540261	62	103514	FJKA01000001-FJKA01000062
H040960441	3540558	64	94267	FJKB01000001-FJKB01000064
H041120007	3541166	58	103178	FJKC01000001-FJKC01000058
H093480403	3541510	64	113613	FJKD01000001-FJKD01000064
H094340202	3538499	59	103523	FJKE01000001-FJKE01000059
H095060125	3539739	57	102062	FJKF01000001-FJKF01000057
H100140151	3539368	61	103525	FJKG01000001-FJKG01000061
H100660110	3540568	63	103524	FJKH01000001-FJKH01000063
H100700025	3541027	56	103510	FJKI01000001-FJKI01000056
H103140121	3540509	57	103518	FJKJ01000001-FJKJ01000057
H103620160	3536019	60	94248	FJKK01000001-FJKK01000060
H103660126	3540470	60	110131	FJKL01000001-FJKL01000060
H103660121	3541153	58	113585	FJKM01000001-FJKM01000058
H104420240	3541670	63	103514	FJKN01000001-FJKN01000063
H110480273	3540984	62	102059	FJKO01000001-FJKO01000062
H112320437	3540480	64	102060	FJKP01000001-FJKP01000064
H112080616	3541936	58	106775	FJKQ01000001-FJKQ01000058
H112380374	3539899	61	110282	FJKR01000001-FJKR01000061
H120160499	3542582	58	102061	FJKS01000001-FJKS01000058
H120200371	3541348	61	103519	FJKT01000001-FJKT01000061
H105140391	3540891	56	113609	FJKU01000001-FJKU01000056
H121040204	3540160	62	106993	FJKV01000001-FJKV01000062
H121420445	3540539	60	103511	FJKW01000001-FJKW01000060
H102240357	3540020	60	102182	FJKZ01000001-FJKZ01000060
H122500497	3541272	61	103526	FJKY01000001-FJKY01000061
H122820408	3540631	61	110131	FJKX01000001-FJKX01000061
H123620597	3390280	61	102055	FJLA01000001-FJLA01000061
H123840629	3539581	60	103523	FJLB01000001-FJLB01000060
H123940534	3542623	63	103518	FJLC01000001-FJLC01000063
H124920387	3541567	62	103510	FJLD01000001-FJLD01000062
H131340777	3540459	62	103526	FJLF01000001-FJLF01000062
H131480353	3659340	64	95680	FJLH01000001-FJLH01000064
H131480354	3710927	70	102060	FJLE01000001-FJLE01000070
H131840211	3660511	71	86362	FJLI01000001-FJLI01000071
H131460248	3541332	61	103521	FJLG01000001-FJLG01000061
H132140863	3538262	58	113601	FJLJ01000001-FJLJ01000058
H053640534/EUL 168	3542142	63	103562	FJLM01000001-FJLM01000063
H064180002	3435971	33	129546	FJLK01000001-FJLK01000033
H064180019	3448814	33	182977	FJMF01000001-FJMF01000033
H043540106	3464056	39	182977	FJLN01000001-FJLN01000039
H044120014	3483235	32	182989	FJLP01000001-FJLP01000032
H052780022	3487895	39	172799	FJLQ01000001-FJLQ01000039
H054280040	3486918	37	176908	FJLO01000001-FJLO01000037
H063680003	3629395	35	182973	FJLR01000001-FJLR01000035
H063840008	3486102	33	238970	FJLS01000001-FJLS01000033

H073660582	3624857	31	255070	FJLT01000001-FJLT01000031
LC5804	3537086	31	183150	FJLU01000001-FJLU01000031
H063760005	3438575	33	177023	FJLV01000001-FJLV01000033
H064240003	3450470	39	183482	FJLW01000001-FJLW01000039
H065040012	3520886	39	176911	FJLY01000001-FJLY01000039
H070140635	3485846	34	183151	FJLZ01000001-FJLZ01000034
H073020039	3486953	39	176940	FJLX01000001-FJLX01000039
H073320399	3485633	31	182851	FJMA01000001-FJMA01000031
H073440003	3489140	34	182973	FJMB01000001-FJMB01000034
LC6009	3452667	37	176933	FJMC01000001-FJMC01000037
H083140015	3542056	34	199082	FJMD01000001-FJMD01000034
H093400182	3476832	33	199435	FJME01000001-FJME01000033
H094760070	3543856	34	185121	FJMJ01000001-FJMJ01000034
H094800237	3545764	33	236407	FJMG01000001-FJMG01000033
H110480715	3560000	38	225261	FJMH01000001-FJMH01000038
H112840293	3553030	35	247920	FJMI01000001-FJMI01000035
H114100406	3452106	33	199452	FJMK01000001-FJMK01000033
H120240362	3544187	37	227709	FJML01000001-FJML01000037
H104640262	3437410	31	183517	FJMM01000001-FJMM01000031
H123140428	3558699	36	181172	FJMN01000001-FJMN01000036
H123460520	3537604	35	183495	FJMO01000001-FJMO01000035
H124360642	3543070	36	176909	FJMP01000001-FJMP01000036
Pontiac-1	3473661	31	204700	FJOA01000001-FJOA01000031

Table S5. Reference genomes used in the SNP-based analysis.
Although the Alcoy, Lens, LPE509 and HL 0604 1035 complete genomes were included in the collection of reference genomes when determining the closest reference to each pair of sequence reads with KmerID, none were used in any mapping analysis. The remaining 27 reference genomes listed in the table were used for mapping one or more isolates.

**Reference name**	**Sequence type (ST)**	**Length of chromosome (bp)**	**Complete genome/PacBio assembly**	**Reference**
Paris	1	3503610	Complete	Cazalet *et al.* 2004
Lorraine	47	3467254	Complete	Gomez-Valero *et al.* 2011
Alcoy	678	3516334	Complete	D'Auria *et al.* 2010
Philadelphia	36	3397754	Complete	Chien *et al.* 2004
Lens	15	3345687	Complete	Cazalet *et al.* 2004
Corby	51	3576470	Complete	Glockner *et al.* 2008
LPE509	Unknown[1]	3434224	Complete	Ma *et al.* 2013
ATCC 43290	187	3359001	Complete	Amaro *et al.* 2012
HL 0604 1035	734	3492535	Complete	Gomez-Valero *et al.* 2011
EUL 28	23	3509586	PacBio assembly (2 contigs: 1 chromosome, 1 plasmid)	This study
EUL 120	42	3430562	PacBio assembly (2 contigs: both chromosomal, 0 plasmids)	This study

EUL 165	37	3474638	PacBio assembly (1 contig: 1 chromosome, 0 plasmids)	This study
H044120014	62	3530817	PacBio assembly (1 contig: 1 chromosome, 0 plasmids)	This study
EUL 2	2	3467814	Illumina assembly (21 contigs)	This study
EUL 7	18	3516217	Illumina assembly (36 contigs)	This study
EUL 18	26	3229839	Illumina assembly (38 contigs)	This study
EUL 25	44	3353388	Illumina assembly (19 contigs)	This study
EUL 26	22	3330854	Illumina assembly (36 contigs)	This study
EUL 32	16	3545771	Illumina assembly (38 contigs)	This study
EUL 36	21	3507348	Illumina assembly (36 contigs)	This study
EUL 40	12	3438629	Illumina assembly (22 contigs)	This study
EUL 48	48	3503195	Illumina assembly (65 contigs)	This study
EUL 49	20	3517591	Illumina assembly (57 contigs)	This study
EUL 51	1156	3405363	Illumina assembly (15 contigs)	This study
EUL 63	77	3461713	Illumina assembly (24 contigs)	This study
EUL 91	63	3266471	Illumina assembly (34 contigs)	This study
EUL 99	34	3257690	Illumina assembly (19 contigs)	This study
EUL 100	59	3351397	Illumina assembly (34 contigs)	This study
EUL 111	25	3298695	Illumina assembly (41 contigs)	This study
EUL 145	78	3365082	Illumina assembly (30 contigs)	This study
EUL 156	1362	3487522	Illumina assembly (22 contigs)	This study

[1]The following allele numbers are called in LPE509: 3 (*flaA*), 10 (*pilE*), 1 (*asd*), 1 (*mip*), 9 (*proA*), and 1 (*neuA*). However, due to the presence of multiple copies of the *mompS* gene, this allele number cannot be determined *in silico*.

Table S6. Reference genomes used for the mapping of all isolates in this study and the coverage achieved.

**EUL/isolate number**	**Reference**	**% reference length mapped**	**Mean depth of coverage**	**Standard deviation of depth of coverage**
**Typing panel**				
1	Paris	98.3	136.4	14.9
2	EUL 2	99.9	136.1	26.4
3	Paris	98.4	138.7	15.4
4	EUL 28	97.0	95.9	20.2
6	EUL 120	98.0	135.7	25.8
7	EUL 7	100.0	145.6	39.7
8	EUL 28	97.0	151.4	24.6
13	Paris	97.2	150.1	23.6
14	Paris	97.2	142.9	21.5
16	Paris	97.2	162.3	23.5
17	Paris	97.8	161.8	21.6
18	EUL 18	100.0	164.2	48.2

19	Philadelphia	94.4	149.2	36.2
20	Philadelphia	94.1	152.1	34.6
25	EUL 25	100.0	156.1	43.1
26	EUL 26	100.0	154.3	44.3
27	EUL 120	98.2	144.8	17.9
27 (replicate)	EUL 120	98.2	98.8	13.6
28	EUL 28	99.0	99.2	12.3
29	EUL 49	98.8	150.8	53.8
30	Philadelphia	93.5	145.4	34.3
31	Lorraine	98.0	144.8	18.9
32	EUL 32	100.0	145.5	44.0
33	Philadelphia	93.4	137.1	35.0
33 (replicate)	Philadelphia	93.2	80.8	21.4
36	EUL 36	99.9	86.5	20.9
37	Paris	97.2	94.9	13.2
38	Paris	97.4	142.4	19.4
39	EUL 120	95.9	154.5	27.1
40	EUL 40	100.0	158.7	45.1
41	EUL 28	97.0	130.1	21.5
42	Paris	98.0	129.4	16.8
43	Paris	98.0	139.1	17.0
48	EUL 48	99.9	126.4	82.1
49	EUL 49	99.9	99.0	36.0
50	EUL 120	98.0	144.8	18.0
51	EUL 51	100.0	143.4	43.3
52	ATCC 43290	94.3	140.5	34.3
53	Paris	98.1	79.5	11.1
54	H044120014	94.6	147.4	51.0
55	Paris	98.3	97.8	14.1
60	Paris	98.4	137.1	17.2
63	EUL 63	99.8	95.9	22.8
66	EUL 63	99.8	99.0	24.4
67	Paris	98.3	130.0	15.2
68	Lorraine	94.8	148.4	31.4
69	EUL 165	95.7	143.2	29.1
69 (replicate)	EUL 165	95.7	96.0	20.4
70	Lorraine	98.0	142.4	19.2
71	H044120014	95.9	97.9	21.1
72	EUL 2	94.8	89.3	26.7
73	EUL 165	95.7	145.3	31.4
74	Philadelphia	97.0	98.2	19.5
75	EUL 120	98.0	133.0	19.5
75 (replicate)	EUL 120	98.0	91.8	14.5
81	Corby	94.0	95.3	20.7
82	Paris	94.2	93.6	20.4
83	Corby	94.5	93.8	19.7
84	Paris	94.2	101.6	21.7

85	Paris	94.3	131.3	27.8
86	Lorraine	95.4	78.5	14.6
87	EUL 36	96.4	150.2	54.6
88	Paris	94.2	111.8	23.7
91	EUL 91	100.0	110.1	29.0
92	Corby	94.2	126.1	27.9
92 (replicate)	Corby	93.8	67.8	16.1
93	Paris	94.1	77.1	17.0
97	ATCC 43290	92.8	95.7	24.7
98	ATCC 43290	92.9	123.5	31.7
99	EUL 99	100.0	153.7	38.0
100	EUL 100	100.0	104.0	24.8
101	EUL 100	97.7	104.8	28.3
102	EUL 100	98.2	132.2	42.8
103	Lorraine	96.2	98.4	17.1
104	Paris	98.1	97.4	13.2
105	EUL 120	97.7	103.6	15.1
110	Paris	97.0	139.1	20.8
111	EUL 111	100.0	107.1	35.8
111 (replicate)	EUL 111	100.0	142.6	47.0
114	Paris	97.4	106.2	15.9
116	EUL 120	95.7	93.2	19.8
117	Paris	97.0	98.8	15.9
118	Philadelphia	98.7	105.7	14.4
119	Paris	97.8	99.6	13.8
120	EUL 120	98.2	112.4	94.0
9	Paris	98.4	140.6	16.6
10	Paris	98.4	133.4	16.4
11	EUL 28	97.0	142.4	38.9
12	EUL 28	97.0	144.8	23.5
22	Philadelphia	92.5	148.6	40.5
23	Philadelphia	92.5	156.4	43.8
24	Philadelphia	92.3	93.2	26.8
34	Philadelphia	93.4	132.4	34.4
35	Philadelphia	93.4	140.6	35.3
44	Paris	98.1	140.4	15.8
45	Paris	98.3	137.0	14.8
46	Paris	97.4	139.5	18.8
47	EUL 40	100.0	157.6	44.5
56	EUL 48	99.9	94.2	59.1
57	H044120014	94.6	140.7	36.1
58	Paris	98.3	128.3	16.5
59	EUL 51	100.0	143.9	44.6
76	H044120014	95.9	127.2	25.7
77	H044120014	95.9	126.2	25.3
78	EUL 165	95.7	92.3	20.5
79	EUL 165	95.8	127.8	26.4

94	Paris	94.2	96.9	20.8
95	Paris	94.2	95.9	20.5
96	Corby	94.0	97.2	21.0
106	ATCC 43290	92.7	71.4	19.1
107	ATCC 43290	92.8	90.4	23.5
121	EUL 120	98.2	149.1	101.8
**Additional isolates**				
LC 202/EUL 153	ATCC 43290	95.2	120.9	26.0
LC 206/EUL 158	ATCC 43290	95.3	131.7	27.9
LC 569/EUL 154	ATCC 43290	92.7	165.3	60.9
LC 606/EUL 155	ATCC 43290	92.7	168.8	62.7
LC 384/EUL 156	EUL 156	99.8	180.5	37.5
LC 395/EUL 159	EUL 156	99.8	86.2	19.1
LC6379-1/EUL 145	EUL 145	99.9	157.3	39.3
LC6376	EUL 145	99.9	128.5	33.4
LC6382	EUL 145	99.9	135.7	34.4
LC6391	EUL 145	99.9	114.4	29.8
LC6394	EUL 145	99.9	130.8	33.4
LC6397	EUL 145	99.9	101.9	26.6
LC6406	EUL 145	99.9	98.7	25.7
LC6407	EUL 145	99.9	111.5	28.5
LC6408	EUL 145	99.9	118.5	29.3
LC6411	EUL 145	99.9	93.8	24.1
LC6412	EUL 145	99.9	115.0	29.8
LC6413	EUL 145	99.9	105.2	26.9
LC6416	EUL 145	99.9	100.1	25.9
LC6418	EUL 145	99.9	94.6	24.1
LC6385	EUL 145	99.9	147.7	38.0
LC6388	EUL 145	99.9	160.3	40.9
LC6409	EUL 145	99.9	159.0	40.3
LC6410	EUL 145	99.9	186.8	46.4
LC0537/EUL 132	EUL 165	97.5	89.2	14.2
LC0539/EUL 133	EUL 165	97.5	145.9	45.0
LC0540/EUL 134	EUL 165	97.5	87.5	15.4
LC0565	EUL 165	97.6	150.3	22.0
LC0583	EUL 165	97.6	155.5	24.0
H034680033	EUL 165	98.8	49.4	13.5
H034680035/EUL 165	EUL 165	99.0	136.0	13.9
H034690056/EUL 166	EUL 165	99.0	145.0	14.7
H034800427	EUL 165	98.9	99.8	35.2
H034980467	EUL 165	99.0	116.7	33.1
H034800423	Paris	97.5	82.7	28.4
OLDA1 (NCTC12008)	Paris	98.4	99.4	14.9
EUL 109	Paris	98.1	132.2	14.8
H064240448	EUL 165	97.9	148.1	24.2
LC0731	EUL 165	94.4	154.9	34.0
LC0732	EUL 165	94.4	153.3	34.7

LC0763	EUL 165	94.4	151.8	32.3
LC0782	EUL 165	94.4	156.9	33.4
LC0795	EUL 165	94.4	139.3	30.5
LC0801	EUL 165	94.3	97.6	21.9
LC5694	EUL 165	95.5	171.6	35.7
LC5722	EUL 165	95.4	159.7	34.6
LC5738	EUL 165	95.5	163.6	36.1
LC5755	EUL 165	97.6	161.5	23.3
LC6163	EUL 165	95.7	155.6	31.9
LC6267	EUL 165	97.6	180.4	25.4
LC6268	EUL 165	97.6	167.7	24.0
LC6228	EUL 165	98.9	141.5	15.4
H041380048	EUL 165	97.9	97.4	17.0
H041640791	EUL 165	97.9	136.7	20.3
H042960010	EUL 165	97.9	165.2	23.6
H061140013	EUL 165	97.6	142.5	23.3
H071880001	EUL 165	95.7	154.1	31.3
H073060003	EUL 165	97.6	137.9	20.8
H080820009	EUL 165	95.8	137.0	27.7
LC6058	EUL 165	97.6	152.0	23.7
LC6293	EUL 165	97.6	163.3	24.8
LC6788	EUL 165	97.9	159.5	25.4
H062660463	EUL 165	95.7	145.7	29.7
H073900557	EUL 165	97.6	130.3	21.1
LC1127	EUL 165	94.4	136.1	30.9
H084760449	EUL 165	98.9	141.1	23.9
H085020185	EUL 165	98.9	140.6	16.0
H090320386	EUL 165	97.9	138.0	22.9
H044260061	EUL 165	98.9	154.8	26.7
H093140322	EUL 165	98.9	138.2	27.2
H093160422	EUL 165	98.9	138.5	16.0
H092760433	EUL 165	97.6	123.0	19.2
H100940111	EUL 165	97.9	107.4	18.7
H101760092	EUL 165	95.7	142.4	29.4
H101820190	EUL 165	97.6	139.2	21.5
H102020414	EUL 165	98.9	149.1	21.5
H101980130	EUL 165	95.7	140.9	29.8
H103820081	EUL 165	97.6	141.5	22.2
H120240685	EUL 165	95.7	113.1	23.5
H104320293	EUL 165	97.6	140.8	24.8
H113180118	EUL 165	95.5	148.6	31.9
H113340664	EUL 165	95.5	141.2	32.6
H113280076	EUL 165	95.8	148.5	28.1
H113660550	EUL 165	95.7	145.5	29.9
H114740454	EUL 165	97.6	135.1	20.3
H115040456	EUL 165	97.6	140.2	20.7
H111580389	EUL 165	98.9	152.0	15.8

H113780240	EUL 165	97.6	135.8	22.6
H083920177	EUL 165	98.8	138.6	15.8
H084140691	EUL 165	97.9	145.8	22.1
H081180019	EUL 165	95.7	150.2	31.9
H103260667	EUL 165	95.7	101.8	21.8
LC464	EUL 165	95.7	137.4	28.2
LC0512	EUL 165	97.6	155.6	35.3
LC0794	EUL 165	97.6	142.8	21.8
LC0798	EUL 165	97.6	156.5	23.2
LC0536/EUL 131	EUL 165	97.6	96.2	15.2
LC230/EUL 122	EUL 120	98.2	112.9	15.6
LC231/EUL 123	EUL 120	98.2	78.7	12.2
LC0462/EUL 124	EUL 120	98.1	90.1	12.6
LC0463/EUL 125	EUL 120	98.0	114.0	14.3
H063920004/EUL 169	Lorraine	98.0	138.5	17.7
H064160534/EULV0410	Lorraine	98.1	102.1	15.2
H064160538/EUL 170	Lorraine	98.0	118.1	15.3
H034700617	Lorraine	97.9	112.4	36.6
H043580159	Lorraine	98.0	96.1	13.8
H043580160	Lorraine	98.0	114.4	15.6
H043660021	Lorraine	98.1	95.5	13.6
H043680663	Lorraine	98.0	96.2	14.4
H043700021	Lorraine	98.1	100.5	14.7
H043790008	Lorraine	98.0	108.2	15.3
H052920051	Lorraine	98.1	104.6	14.3
H053540106	Lorraine	98.0	106.0	17.5
H063660005	Lorraine	97.9	169.9	22.3
H063660006	Lorraine	97.9	125.7	19.7
H063760006	Lorraine	97.9	196.0	24.4
H063660009	Lorraine	97.9	137.2	18.2
H063680006	Lorraine	97.9	149.7	20.1
H063680007	Lorraine	97.9	128.1	17.6
H063740003	Lorraine	97.9	125.6	23.9
H063740018	Lorraine	97.9	153.9	20.7
H063780007	Lorraine	97.9	133.7	20.8
H063780008	Lorraine	97.9	191.3	26.4
H063860003	Lorraine	97.9	123.8	20.5
H063960001	Lorraine	97.9	134.5	20.1
LC5759	Lorraine	98.0	99.1	13.9
H070420013	Lorraine	98.1	104.7	14.8
LC5822	Lorraine	98.1	106.2	15.3
H040260015	Lorraine	97.9	153.5	19.8
H055140095	Lorraine	98.0	86.3	12.7
H060780053	Lorraine	97.9	164.0	21.3
H061120064	Lorraine	97.9	139.1	19.8
H062840608	Lorraine	97.9	160.0	19.7
H062940111	Lorraine	97.9	143.6	50.8

H064320006	Lorraine	97.9	158.9	33.5
H064280005	Lorraine	97.9	136.6	26.4
H064380002	Lorraine	97.9	139.5	18.4
H064380001	Lorraine	97.9	138.9	24.0
H064560527	Lorraine	97.9	141.5	25.7
H064660638	Lorraine	98.1	94.7	15.5
H070160015	Lorraine	98.1	94.9	15.3
H071120010	Lorraine	97.9	134.1	19.6
H071360036	Lorraine	97.9	211.3	29.9
H072740002	Lorraine	97.9	123.9	28.7
H073000045	Lorraine	97.9	124.7	17.8
H073380007	Lorraine	98.1	102.4	14.4
H073600182	Lorraine	98.1	112.4	18.0
H073640185	Lorraine	97.9	129.7	19.1
H074960018	Lorraine	97.9	141.7	21.5
H080780059	Lorraine	97.9	152.6	19.9
H053840008	Lorraine	98.1	90.4	13.1
H072520002	Lorraine	97.9	135.0	18.6
H081340222	Lorraine	97.9	177.0	22.9
H082520613	Lorraine	97.9	141.4	23.3
H083120262	Lorraine	98.1	96.9	13.8
H083620580	Lorraine	97.9	122.3	20.8
H083960064	Lorraine	97.9	106.1	16.7
H084620118	Lorraine	98.1	92.3	13.1
H090140214	Lorraine	98.0	90.1	13.2
H090440226	Lorraine	98.0	99.7	15.6
H040960441	Lorraine	98.0	98.9	14.0
H041120007	Lorraine	98.1	96.5	14.6
H093480403	Lorraine	98.1	97.0	14.1
H094340202	Lorraine	98.1	91.1	12.5
H095060125	Lorraine	98.1	108.6	14.9
H100140151	Lorraine	98.0	94.2	13.6
H100660110	Lorraine	98.0	91.7	13.4
H100700025	Lorraine	98.0	95.3	16.8
H103140121	Lorraine	98.1	102.4	15.6
H103620160	Lorraine	98.1	91.6	13.2
H103660126	Lorraine	98.1	99.1	14.3
H103660121	Lorraine	98.0	105.4	14.6
H104420240	Lorraine	98.0	101.6	14.5
H110480273	Lorraine	98.0	102.2	14.6
H112320437	Lorraine	98.0	98.5	15.1
H112080616	Lorraine	98.1	104.6	14.6
H112380374	Lorraine	98.0	109.6	14.8
H120160499	Lorraine	98.1	108.4	15.3
H120200371	Lorraine	98.1	104.0	17.1
H105140391	Lorraine	98.1	116.2	17.0
H121040204	Lorraine	98.0	95.1	13.7

H121420445	Lorraine	98.0	99.7	14.3
H102240357	Lorraine	98.1	88.6	13.4
H122500497	Lorraine	98.0	103.4	14.8
H122820408	Lorraine	98.1	103.5	14.8
H123620597	Lorraine	98.1	95.9	13.7
H123840629	Lorraine	98.1	92.6	13.2
H123940534	Lorraine	98.1	105.6	15.9
H124920387	Lorraine	98.1	105.0	14.4
H131340777	Lorraine	98.0	99.7	15.0
H131480353	Lorraine	98.0	89.3	13.9
H131480354	Lorraine	98.1	94.6	14.6
H131840211	Lorraine	98.0	88.6	14.6
H131460248	Lorraine	98.1	97.3	14.1
H132140863	Lorraine	98.0	93.4	18.3
H053640534/EUL 168	Lorraine	98.0	150.8	18.6
H064180002	H044120014	92.4	98.1	27.9
H064180019	H044120014	92.4	109.0	29.7
H043540106	H044120014	95.8	108.2	21.5
H044120014	H044120014	98.8	110.1	15.4
H052780022	H044120014	95.9	92.3	19.9
H054280040	H044120014	95.3	88.2	20.2
H063680003	H044120014	98.2	91.5	13.1
H063840008	H044120014	95.3	107.5	23.5
H073660582	H044120014	98.2	106.7	16.7
LC5804	H044120014	95.5	82.8	17.4
H063760005	H044120014	94.8	103.8	27.1
H064240003	H044120014	94.6	100.6	28.6
H065040012	H044120014	94.6	84.7	22.2
H070140635	H044120014	95.3	90.0	19.8
H073020039	H044120014	95.3	92.9	22.2
H073320399	H044120014	95.3	97.7	27.5
H073440003	H044120014	95.3	114.0	26.9
LC6009	H044120014	94.5	80.1	21.8
H083140015	H044120014	94.6	97.4	24.4
H093400182	H044120014	94.9	100.3	34.8
H094760070	H044120014	95.4	96.4	21.9
H094800237	H044120014	95.4	95.6	22.3
H110480715	H044120014	95.8	86.5	29.9
H112840293	H044120014	96.1	92.5	20.5
H114100406	H044120014	94.6	102.7	27.9
H120240362	H044120014	95.4	86.1	18.0
H104640262	H044120014	95.5	89.4	18.7
H123140428	H044120014	95.3	95.9	22.2
H123460520	H044120014	95.5	96.3	22.4
H124360642	H044120014	95.4	88.5	18.7
Pontiac-1	H044120014	95.4	101.4	33.7

Table S7. 370 *L. pneumophila* isolates used to define the total core gene content of the species.

Isolate name	Reference/ accession no.		Isolate name	Reference/ accession no.		Isolate name	Reference/ accession no.
EUL 1	ERR376626		EUL 145	ERR376769		H122820408	ERR363980
EUL 2	ERR376627		EUL 148	ERR376772		H122500497	ERR363981
EUL 3	ERR376628		EUL 149	ERR376773		H121040204	ERR363982
EUL 4	ERR376721		EUL 150	ERR376774		H121420445	ERR363983
EUL 5	ERR376630		EUL 153	ERR376775		H120200371	ERR363984
EUL6	ERR376631		EUL 154	ERR376776		H120160499	ERR363985
EUL 7	ERR376632		EUL 155	ERR376777		H131840211	ERR363986
EUL 8	ERR376633		EUL 156	ERR376778		H131460248	ERR363987
EUL 9	ERR376634		EUL 157	ERR376779		H131480354	ERR363988
EUL 10	ERR376635		EUL 158	ERR376780		H131480353	ERR363989
EUL 11	ERR376636		EUL 159	ERR352158		H124920387	ERR363991
EUL 12	ERR376637		EUL 161	ERR376781		H120240685	ERR363992
EUL 13	ERR376646		EUL 162	ERR376782		H105140391	ERR363993
EUL 14	ERR376639		EUL 163	ERR376783		H064160534	ERR363994
EUL 16	ERR376641		EUL 164	ERR376784		H043540106	ERR363997
EUL 17	ERR376642		EUL 165	ERR376785		H052780022	ERR363998
EUL 18	ERR376643		EUL 166	ERR376786		H044120014	ERR363999
EUL 19	ERR376644		EUL 167	ERR352160		H063760005	ERR364000
EUL 20	ERR376645		EUL 168	ERR352161		H063840008	ERR364001
EUL 21	ERR376638		EUL 169	ERR376787		H063680003	ERR364002
EUL 22	ERR376647		EUL 170	ERR376788		H094760070	ERR364003
EUL 23	ERR376648		H123640643	ERR332166		H064180019	ERR364004
EUL 24	ERR332110		H041380048	ERR363843		H064240003	ERR364005
EUL 25	ERR376650		H041640791	ERR363844		H093400182	ERR364006
EUL 26	ERR376651		H042960010	ERR363845		H083140015	ERR364007
EUL 27	ERR376652		H044260061	ERR363846		H073660582	ERR364008
EUL 28	ERR376722		H061140013	ERR363847		H073320399	ERR364010
EUL 30	ERR376655		H062660463	ERR363848		H070140635	ERR364011
EUL 31	ERR376656		H064240448	ERR363849		H124360642	ERR364013
EUL 32	ERR376657		H071880001	ERR363850		H123460520	ERR364014
EUL 33	ERR376658		H073060003	ERR363851		H123140428	ERR364015
EUL 34	ERR376659		H073900557	ERR363852		H114100406	ERR364016
EUL 35	ERR376660		H080820009	ERR363853		H112840293	ERR364017
EUL 36	ERR332122		H081180019	ERR363854		H110480715	ERR364018
EUL 37	ERR376723		H083920177	ERR363855		H104640262	ERR364019
EUL 38	ERR376663		H084140691	ERR363856		H094800237	ERR364020
EUL 40	ERR376665		H084760449	ERR363857		H064180002	ERR364021
EUL 41	ERR376666		H085020185	ERR363858		H073020039	ERR364022
EUL 42	ERR376667		H090320386	ERR363859		H073340594	ERR364023
EUL 43	ERR376668		H092760433	ERR363860		H073240536	ERR364024
EUL 44	ERR376669		H093140322	ERR363861		H120240362	ERR364025
EUL 45	ERR376670		H093160422	ERR363862		H073280012	ERR364026
EUL 46	ERR376671		H100940111	ERR363863		H073340034	ERR364027

EUL 47	ERR376672		H101760092	ERR363864		H054280040	ERR364028
EUL 48	ERR332134		H101820190	ERR363865		H132140863	ERR364031
EUL 50	ERR376675		H101980130	ERR363866		H092380261	ERR434063
EUL 51	ERR376676		H102020414	ERR363867		H092400768	ERR434064
EUL 52	ERR376677		H103820081	ERR363868		LC6385	ERR352162
H041120007	ERR363942		H104320293	ERR363869		LC6388	ERR352163
EUL 53	ERR376725		H111580389	ERR363870		LC6409	ERR352164
EUL 54	ERR376679		H113180118	ERR363871		LC6410	ERR352165
EUL 55	ERR332141		H113280076	ERR363872		LC464	ERR363878
EUL 56	ERR376726		H113340664	ERR363873		LC0512	ERR363879
EUL 57	ERR376682		H113660550	ERR363874		LC0565	ERR363880
EUL 58	ERR376683		H113780240	ERR363875		LC0583	ERR363881
EUL 60	ERR376685		H114740454	ERR363876		LC0731	ERR363882
EUL 61	ERR376686		H115040456	ERR363877		LC0732	ERR363883
EUL 62	ERR376687		H040260015	ERR363903		LC0763	ERR363884
EUL 63	ERR332149		H063660005	ERR363904		LC0782	ERR363885
EUL 64	ERR376727		H063740018	ERR363906		LC0794	ERR363886
EUL 66	ERR376728		H060780053	ERR363907		LC0795	ERR363887
EUL 67	ERR376692		H071360036	ERR363908		LC0801	ERR363889
EUL 68	ERR376693		H081340222	ERR363909		LC1127	ERR363890
EUL 69	ERR376694		H080780059	ERR363910		LC5694	ERR363891
EUL 70	ERR376695		H082520613	ERR363912		LC5722	ERR363892
EUL 71	ERR332157		H063680007	ERR363913		LC5738	ERR363893
EUL 72	ERR332158		H061120064	ERR363914		LC5755	ERR363894
EUL 73	ERR376698		H063760006	ERR363915		LC6058	ERR363896
EUL 74	ERR376729		H063780008	ERR363916		LC6163	ERR363897
EUL 75	ERR376700		H062840608	ERR363917		LC6228	ERR363898
EUL 76	ERR376701		H063680006	ERR363918		LC6267	ERR363899
EUL 77	ERR376702		H062940111	ERR363919		LC6268	ERR363900
EUL 78	ERR376730		H074960018	ERR363920		LC6293	ERR363901
EUL 81	ERR376732		H064380001	ERR363921		LC6788	ERR363902
EUL 82	ERR376733		H063660006	ERR363922		LC5759	ERR363995
EUL 83	ERR376734		H064320006	ERR363923		LC5822	ERR363996
EUL 84	ERR376735		H064280005	ERR363924		LC5804	ERR364029
EUL 85	ERR376710		H064560527	ERR363925		LC6009	ERR364030
EUL 86	ERR332172		H064380002	ERR363926		LC6376	ERR376790
EUL 87	ERR376712		H072520002	ERR363927		LC6382	ERR376792
EUL 88	ERR332174		H063960001	ERR363928		LC6391	ERR376793
EUL 90	ERR376736		H063740003	ERR363929		LC6394	ERR376794
EUL 91	ERR376737		H063860003	ERR363930		LC6397	ERR376795
EUL 92	ERR376717		H071120010	ERR363931		LC6406	ERR376796
EUL 93	ERR332179		H073000045	ERR363932		LC6407	ERR376797
EUL 94	ERR376738		H073640185	ERR363933		LC6408	ERR376798
EUL 95	ERR376739		H063780007	ERR363934		LC6411	ERR376799
EUL 96	ERR376740		H083620580	ERR363936		LC6412	ERR376800
EUL 97	ERR376741		H083960064	ERR363937		LC6413	ERR376801
EUL 98	ERR376629		H103260667	ERR363938		LC6416	ERR376802

EUL 100	ERR376742		H084620118	ERR363939		LC6418	ERR376804
EUL 101	ERR376743		H073380007	ERR363940		OLDA1	ERR434061
EUL 102	ERR376714		H083120262	ERR363941		Alcoy	D'Auria *et al.* 2010
EUL 103	ERR376744		H043700021	ERR363944		ATCC 43290	Amaro *et al.* 2012
EUL 104	ERR376745		H043790008	ERR363945		Corby	Glockner *et al.* 2008
EUL 105	ERR376746		H043660021	ERR363946		HL 0604 1035	Gomez-Valero *et al.* 2011
EUL 107	ERR376747		H055140095	ERR363947		Lorraine	Gomez-Valero *et al.* 2011
EUL 108	ERR376748		H053540106	ERR363948		Thunderbay	Khan *et al.* 2013
EUL 109	ERR376662		H043680663	ERR363949		Lens	Cazalet *et al.* 2004
EUL 110	ERR376674		H103620160	ERR363950		Paris	Cazalet *et al.* 2004
EUL 111	ERR376749		H112320437	ERR363951		Philadelphia	Chien *et al.* 2004
EUL 113	ERR363968		H112080616	ERR363952		H043940028	Underwood *et al.* 2013
EUL 114	ERR363969		H040960441	ERR363953		H044500045	Underwood *et al.* 2013
EUL 115	ERR376753		H053840008	ERR363954		H044540088	Underwood *et al.* 2013
EUL 116	ERR376754		H102240357	ERR363955		H063280001	Underwood *et al.* 2013
EUL 117	ERR376755		H104420240	ERR363957		H065000139	Underwood *et al.* 2013
EUL 118	ERR340981		H100700025	ERR363958		H070840415	Underwood *et al.* 2013
EUL 119	ERR376757		H043580160	ERR363959		H071260094	Underwood *et al.* 2013
EUL 120	ERR376758		H112380374	ERR363960		H074360702	Underwood *et al.* 2013
EUL 121	ERR376678		H052920051	ERR363961		H074360710	Underwood *et al.* 2013
EUL 122	ERR376759		H100660110	ERR363962		H075160080	Underwood *et al.* 2013
EUL 123	ERR332142		H090140214	ERR363963		H090500162	Underwood *et al.* 2013
EUL 124	ERR332150		H064660638	ERR363964		H091960009	Underwood *et al.* 2013
EUL 125	ERR376760		H100140151	ERR363965		H091960011	Underwood *et al.* 2013
EUL 129	ERR376762		H090440226	ERR363966		H093380153	Underwood *et al.* 2013
EUL 130	ERR376703		H103140121	ERR363967		H093620212	Underwood *et al.* 2013
EUL 131	ERR332167		H070160015	ERR363970		H100260089	Underwood *et al.* 2013
EUL 132	ERR332168		H094340202	ERR363971		LC3330	Underwood *et al.* 2013
EUL 133	ERR332169		H093480403	ERR363973		LC6451	Underwood *et al.* 2013
EUL 134	ERR332170		H103660126	ERR363974		LC6774	Underwood *et al.* 2013
EUL 140	ERR376653		H123940534	ERR363975		RR08000517	Underwood *et al.* 2013
EUL 141	ERR376765		H073600182	ERR363976		RR08000760	Underwood *et al.* 2013
EUL 142	ERR376766		H123840629	ERR363978			
EUL 143	ERR376767		H123620597	ERR363979			

Table S8. Genes used in the cgMLST schemes with 50, 100, 500 or 1455 core genes. The previously published Philadelphia genome was used as the reference.

**Gene**	**Product**	**Length (bp)**	**Scheme (number of genes)**
lpg0085	hypothetical protein	528	50, 100, 500, 1455
lpg0104	peptide methionine sulfoxide reductase	576	50, 100, 500, 1455
lpg0131	dihydropicolinate reductase	732	50, 100, 500, 1455
lpg0136	pyruvate kinase II	1425	50, 100, 500, 1455
lpg0189	hypothetical protein	867	50, 100, 500, 1455
lpg0245	NAD-glutamate dehydrogenase	3381	50, 100, 500, 1455
lpg0329	50S ribosomal protein L3	651	50, 100, 500, 1455
lpg0331	50S ribosomal protein L23	279	50, 100, 500, 1455
lpg0409	hypothetical, SURF1 family	729	50, 100, 500, 1455
lpg0419	glucokinase	1008	50, 100, 500, 1455
lpg0525	hypothetical virulence protein	627	50, 100, 500, 1455
lpg0596	hypothetical protein	696	50, 100, 500, 1455
lpg0601	ABC transporter, permease	1449	50, 100, 500, 1455
lpg0607	lysyl tRNA synthetase	954	50, 100, 500, 1455
lpg0622	transmembrane protein	1944	50, 100, 500, 1455
lpg0664	D-ribulose-5-phosphate-3-epimerase	654	50, 100, 500, 1455
lpg0689	DNA binding stress protein	441	50, 100, 500, 1455
lpg0700	protein-L-isoaspartate-O-methyltransferase	675	50, 100, 500, 1455
lpg0812	rod shape determining protein MreC	909	50, 100, 500, 1455
lpg0866	3-methyladenine DNA glycosylase	552	50, 100, 500, 1455
lpg0871	hypothetical protein	867	50, 100, 500, 1455
lpg0890	cystathionine beta-lyase	1152	50, 100, 500, 1455
lpg0957	hypothetical protein	906	50, 100, 500, 1455
lpg1323	drug resistance transporter, Bcr/CflA	1161	50, 100, 500, 1455
lpg1503	pyruvate dehydrogenase E2 component	1653	50, 100, 500, 1455
lpg1534	glutamate-1-semialdehyde-2,1-aminomutase	1302	50, 100, 500, 1455
lpg1543	transmembrane protein	675	50, 100, 500, 1455
lpg1586	hypothetical protein	378	50, 100, 500, 1455
lpg1737	glutamyl/tRNA (Gln) amidotransferase, B subunit	1434	50, 100, 500, 1455
lpg1744	HesB family protein	369	50, 100, 500, 1455
lpg1759	flagellar motor switch protein FliG	990	50, 100, 500, 1455
lpg1811	aspartokinase	2580	50, 100, 500, 1455
lpg1869	ribonuclease III	675	50, 100, 500, 1455
lpg1909	hypothetical protein	1005	50, 100, 500, 1455
lpg2229	saframycin Mx1 synthetase B	1746	50, 100, 500, 1455
lpg2264	hypothetical protein	315	50, 100, 500, 1455
lpg2331	biotin synthase BioC	1005	50, 100, 500, 1455
lpg2349	alkylhydroperoxidase AhpD family core domain protein	564	50, 100, 500, 1455
lpg2387	plasminogen activator	927	50, 100, 500, 1455
lpg2494	hypothetical protein	693	50, 100, 500, 1455
lpg2528	alpha-amylase, putative	1551	50, 100, 500, 1455

lpg2597	DNA processing enzyme DprA (SMF family)	1086	50, 100, 500, 1455
lpg2633	hypothetical protein	318	50, 100, 500, 1455
lpg2654	GTP binding protein	1092	50, 100, 500, 1455
lpg2691	cation transporting ATPase PacS	2544	50, 100, 500, 1455
lpg2699	ATPase or kinase	483	50, 100, 500, 1455
lpg2864	hypothetical protein	1119	50, 100, 500, 1455
lpg2878	cobalt/magnesium uptake transporter	1065	50, 100, 500, 1455
lpg2882	methionyl tRNA synthetase	2082	50, 100, 500, 1455
lpg2902	hypothetical protein	426	50, 100, 500, 1455
lpg0011	thiol-disulfide oxidoreductase ResA	471	100, 500, 1455
lpg0014	transmembrane protein	1212	100, 500, 1455
lpg0033	hypothetical protein	1026	100, 500, 1455
lpg0079	2-polyprenyl-6-methoxyphenol hydroxylase	1164	100, 500, 1455
lpg0127	acetyl-coenzyme A synthetase	1884	100, 500, 1455
lpg0287	translation elongation factor P (EF-P)	618	100, 500, 1455
lpg0415	hypothetical protein	246	100, 500, 1455
lpg0531	succinate dehydrogenase iron-sulfur protein subunit B	723	100, 500, 1455
lpg0540	major facilitator family transporter	1284	100, 500, 1455
lpg0551	1-acyl-sn-glycerol-3-phosphate acetyltransferase	774	100, 500, 1455
lpg0581	hypothetical protein	186	100, 500, 1455
lpg0606	metal-sulfur cluster biosynthetic enzyme	372	100, 500, 1455
lpg0650	50S ribosomal protein L31	228	100, 500, 1455
lpg0785	acetyl CoA carboxylase, carboxyltransferase, alpha subunit	954	100, 500, 1455
lpg0880	hypothetical protein	642	100, 500, 1455
lpg0963	hypothetical protein	1242	100, 500, 1455
lpg1202	cytochrome D ubiquinol oxidase, subunit I	1533	100, 500, 1455
lpg1225	flagellar hook associated protein 1 FlgK	1950	100, 500, 1455
lpg1298	hypothetical protein	201	100, 500, 1455
lpg1302	tRNA pseudouridine synthase A	789	100, 500, 1455
lpg1366	hypothetical protein	774	100, 500, 1455
lpg1386	enhanced entry protein EnhA	474	100, 500, 1455
lpg1396	acyl carrier protein	249	100, 500, 1455
lpg1457	GTP pyrophosphokinase ((p)ppGpp synthetase I) stringent stress response RelA	2205	100, 500, 1455
lpg1565	thiamine biosynthesis protein NMT-1	951	100, 500, 1455
lpg1576	Holliday junction DNA helicase RuvB	1032	100, 500, 1455
lpg1690	aconitate hydratase	2676	100, 500, 1455
lpg1772	hypothetical protein	777	100, 500, 1455
lpg1844	D-tyrosyl-tRNA	438	100, 500, 1455
lpg1916	possible regulator of murein genes BolA	318	100, 500, 1455
lpg2008	endoribonuclease L-PSP	387	100, 500, 1455
lpg2053	hypothetical protein	879	100, 500, 1455
lpg2191	global stress protein GspA	528	100, 500, 1455
lpg2209	hypothetical protein	531	100, 500, 1455
lpg2299	ATP-dependent RNA helicase	2877	100, 500, 1455
lpg2317	transmembrane protein	1161	100, 500, 1455

lpg2333	membrane associated zinc metalloprotease	1092	100, 500, 1455
lpg2337	protein methyltransferase HemK	864	100, 500, 1455
lpg2345	ATP-dependent RNA helicase	1770	100, 500, 1455
lpg2481	integral membrane protein	903	100, 500, 1455
lpg2594	methionyl tRNA formyltransferase	945	100, 500, 1455
lpg2620	chromosome segregation SMC protein	3495	100, 500, 1455
lpg2623	transmembrane protein	813	100, 500, 1455
lpg2627	hypothetical protein	1182	100, 500, 1455
lpg2657	ferrous iron transporter B	2256	100, 500, 1455
lpg2674	DotD	492	100, 500, 1455
lpg2764	inorganic pyrophosphatase	537	100, 500, 1455
lpg2843	inosine 5'-monophosphate dehydrogenase	1014	100, 500, 1455
lpg2930	sec-independent (periplasmic) protein translocase protein TatC	726	100, 500, 1455
lpg3005	50S ribosomal protein L34	135	100, 500, 1455
lpg0001	chromosomal replication initiator protein DnaA	1359	500, 1455
lpg0009	host factor-I protein for bacteriophage Q beta replication	258	500, 1455
lpg0010	GTP binding protein HflX	1260	500, 1455
lpg0018	outer membrane efflux protein	1386	500, 1455
lpg0024	hemin binding protein Hbp	426	500, 1455
lpg0027	low affinity inorganic phosphate transporter	996	500, 1455
lpg0059	hypothetical protein	1107	500, 1455
lpg0078	2-octaprenyl-6-methoxyphenol hydroxylase	1203	500, 1455
lpg0083	glutathione synthase/ribosomal protein S6 modification enzyme	948	500, 1455
lpg0084	hypothetical protein	1524	500, 1455
lpg0098	two component sensor and regulator, histidine kinase response regulator	453	500, 1455
lpg0099	DNA polymerase I	2691	500, 1455
lpg0101	hypothetical protein	783	500, 1455
lpg0103	N-terminal acetyltransferase, GNAT family	861	500, 1455
lpg0116	glycine cleavage system protein P	1371	500, 1455
lpg0118	glycine cleavage system T protein	1104	500, 1455
lpg0120	IcmL-like	531	500, 1455
lpg0128	3-hydroxyisobutyrate dehydrogenase	918	500, 1455
lpg0137	phosphoglycerate kinase	1191	500, 1455
lpg0165	hypothetical protein	456	500, 1455
lpg0175	pyoverdine biosynthesis protein PvcB	837	500, 1455
lpg0212	deoxyribodipyrimidine photolyase	1416	500, 1455
lpg0218	phosphoribosylaminoimidazole carboxylase, catalytic subunit PurE	501	500, 1455
lpg0232	transcriptional regulator np20, Fur family	534	500, 1455
lpg0241	glutaminase	933	500, 1455
lpg0248	arsenate reductase	342	500, 1455
lpg0252	membrane protein	696	500, 1455
lpg0257	multidrug resistance secretion protein	993	500, 1455
lpg0260	hypothetical protein	399	500, 1455
lpg0268	hypothetical protein	597	500, 1455

lpg0288	L-lysine 2,3-aminomutase, radical SAM domain protein	981	500, 1455
lpg0289	polyphosphate kinase	2085	500, 1455
lpg0290	lipoprotein	1206	500, 1455
lpg0291	chromate transport protein	534	500, 1455
lpg0293	long chain acyl-CoA dehydrogenase	2439	500, 1455
lpg0294	hypothetical protein	693	500, 1455
lpg0296	hypothetical phosphotransferase	1047	500, 1455
lpg0317	transcription antitermination protein NusG	555	500, 1455
lpg0318	50S ribosomal protein L11	435	500, 1455
lpg0342	30S ribosomal protein S14	288	500, 1455
lpg0346	30S ribosomal protein S5	507	500, 1455
lpg0352	30S ribosomal protein S11	399	500, 1455
lpg0354	DNA-directed RNA polymerase alpha subunit RpoA	993	500, 1455
lpg0362	3-oxoacyl-(acyl carrier protein) synthase II, N-terminal	1278	500, 1455
lpg0376	SdhA, GRIP coiled-coil protein GCC185	4290	500, 1455
lpg0383	hypothetical protein	483	500, 1455
lpg0385	LemA protein	582	500, 1455
lpg0386	heat shock protein HtpX	1020	500, 1455
lpg0388	ABC transporter, ATP binding component	915	500, 1455
lpg0408	inner (transmembrane) protein	549	500, 1455
lpg0410	hypothetical protein	546	500, 1455
lpg0411	cytochrome c oxidase assembly protein	1032	500, 1455
lpg0414	glutathione synthase, ribosomal protein S6 modification protein	909	500, 1455
lpg0439	hypothetical protein	1050	500, 1455
lpg0453	IcmC (DotE)	585	500, 1455
lpg0456	IcmB (DotO)	3030	500, 1455
lpg0461	ribosomal protein L11 methyltransferase	870	500, 1455
lpg0463	acetyl CoA carboxylase, biotin carboxyl carrier protein	483	500, 1455
lpg0464	3-dehydroquinate dehydratase type II	438	500, 1455
lpg0474	CDP-diacylglycerol-serine-O-phosphatidyltransferase	744	500, 1455
lpg0477	RNA polymerase signma-54 factor RpoN	1395	500, 1455
lpg0479	50S ribosomal protein L28	237	500, 1455
lpg0481	S-adenosylmethionine-dependent methyltransferase	681	500, 1455
lpg0483	ankyrin repeat-containing protein	1488	500, 1455
lpg0485	HflC protein	921	500, 1455
lpg0493	amino acid (glutamine) ABC transporter, ATP binding component	669	500, 1455
lpg0497	adenosine deaminase	1476	500, 1455
lpg0529	succinate dehydrogenase hydrophobic membrane anchor protein subunit D	348	500, 1455
lpg0532	2-oxoglutarate dehydrogenase E1 component)	2835	500, 1455
lpg0533	dihydrolipoamide succinyltransferase	1230	500, 1455
lpg0536	pyridoxamine 5'-phosphate oxidase	648	500, 1455
lpg0541	probable membrane protein YdgA-like	1485	500, 1455
lpg0557	formamidopyrimidine DNA glycosylase	825	500, 1455

lpg0559	hypothetical protein	399	500, 1455
lpg0564	hypothetical protein	1071	500, 1455
lpg0565	spore maturation protein A	618	500, 1455
lpg0580	adenosine deaminase	981	500, 1455
lpg0586	transcriptional regulator	564	500, 1455
lpg0593	5-formyltetrahydrofolate cyclo-ligase	582	500, 1455
lpg0595	4-amino-4-deoxychorismate lyase	816	500, 1455
lpg0598	hypothetical protein	420	500, 1455
lpg0599	poly-beta-hydroxybutyrate polymerase	1761	500, 1455
lpg0600	rrf2 family protein	462	500, 1455
lpg0603	ABC transporter, permease component	1287	500, 1455
lpg0611	metal ion transporter	1314	500, 1455
lpg0618	3-methyladenine DNA glycosylase	573	500, 1455
lpg0623	hypothetical protein	393	500, 1455
lpg0629	Tfp pilus assembly protein PilX	513	500, 1455
lpg0631	type IV fimbrial biogenesis protein PilV	540	500, 1455
lpg0633	polysaccharide deacetylase	906	500, 1455
lpg0634	hypothetical protein	1350	500, 1455
lpg0662	multidrug efflux MFS outer membrane protein (RND family)	1440	500, 1455
lpg0667	hypothetical protein	870	500, 1455
lpg0670	hypothetical protein	1083	500, 1455
lpg0686	thiol:disulfide interchange protein DsbD	1791	500, 1455
lpg0697	sulfate transporter	2307	500, 1455
lpg0701	2-amino-3-ketobutyrate coenzyme A ligase	1248	500, 1455
lpg0726	ATP cone and Zn ribbon domains protein	468	500, 1455
lpg0729	phosphatidylglycerophosphatase A (PgpA)	483	500, 1455
lpg0745	lipoic acid synthetase	990	500, 1455
lpg0748	LPS biosynthesis protein, PseA-like	1383	500, 1455
lpg0760	glucose-1-phosphate thymidylyltransferase RmlA	918	500, 1455
lpg0800	L-aspartate oxidase	1650	500, 1455
lpg0801	adenylsuccinate lyase	1371	500, 1455
lpg0803	acyl CoA dehydrogenase, short chain specific	1704	500, 1455
lpg0805	phosphoenolpyruvate synthase	2388	500, 1455
lpg0808	UDP-N-acetylglucosamine-N-acetylmuramyl-(pentapeptide) pyrophosphoryl-undecaprenol N-acetylglucosamine transferase	1092	500, 1455
lpg0817	hypothetical protein	336	500, 1455
lpg0821	lipopolysaccharide biosynthesis glycosyltransferase	780	500, 1455
lpg0822	O-antigen biosynthesis protein	1251	500, 1455
lpg0823	neurogenic locus notch protein homolog precursor	375	500, 1455
lpg0824	rhomboid family protein	600	500, 1455
lpg0825	peptidase, M23/M37 family	915	500, 1455
lpg0834	anthranilate phosphoribosyltransferase	1035	500, 1455
lpg0839	hydrolase, HAD superfamily, subfamily III A	552	500, 1455
lpg0840	polysialic acid capsule expression protein	963	500, 1455
lpg0841	toluene tolerance ABC transporter, ATP binding protein Ttg2A	798	500, 1455
lpg0849	ABC transporter, ATP binding protein	678	500, 1455

lpg0853	transcriptional regulator FleQ	1416	500, 1455
lpg0854	hypothetical protein	282	500, 1455
lpg0865	cytochrome c type biogenesis protein CycH	405	500, 1455
lpg0870	3-hydroxyisobutyryl Coenzyme A hydrolase	1059	500, 1455
lpg0873	hypothetical protein	426	500, 1455
lpg0879	two component response regulator with GGDEF domain	1143	500, 1455
lpg0885	glycosyl hydrolase	1077	500, 1455
lpg0886	sodium:dicarboxylate symporter	1281	500, 1455
lpg0891	sensory box protein/GGDEF/EAL domains	2316	500, 1455
lpg0895	hypothetical protein	510	500, 1455
lpg0901	hypothetical protein NMA0899	657	500, 1455
lpg0911	Bvg accessory factor	771	500, 1455
lpg0919	transmembrane protein	660	500, 1455
lpg0921	hypothetical protein	1245	500, 1455
lpg0922	electron transfer flavoprotein, beta subunit	750	500, 1455
lpg0923	electron transfer flavoprotein, alpha subunit	939	500, 1455
lpg0924	alanine dehydrogenase	1122	500, 1455
lpg0930	type IV pilus biogenesis protein PilP	588	500, 1455
lpg0936	riboflavin biosynthesis RibF	987	500, 1455
lpg0950	nitrilase	807	500, 1455
lpg0954	transcription repair coupling factor	3462	500, 1455
lpg0958	DNA ligase	2052	500, 1455
lpg0971	ecto-ATP diphosphohydrolase II	1146	500, 1455
lpg1121	hypothetical protein	771	500, 1455
lpg1139	spermidine/putrescine ABC transporter permease protein PotC	768	500, 1455
lpg1140	spermidine/putrescine ABC transporter permease protein PotB	825	500, 1455
lpg1141	spermidine/putrescine ABC transporter, ATP-binding protein PotA	1149	500, 1455
lpg1143	short chain type dehydrogenase/reductase	885	500, 1455
lpg1154	hypothetical protein	1083	500, 1455
lpg1157	lipase B	765	500, 1455
lpg1159	permeases of drug/transporter	1038	500, 1455
lpg1162	OmpA-like transmembrane domain protein	732	500, 1455
lpg1179	riboflavin biosynthesis protein RibA	1209	500, 1455
lpg1180	riboflavin synthase, beta subunit	468	500, 1455
lpg1198	histidinol-phosphate aminotransferase	1095	500, 1455
lpg1218	flagellar basal body rod modification protein FlgD	678	500, 1455
lpg1219	flagellar hook protein FlgE	1314	500, 1455
lpg1226	flagellar hook associated protein type 3 FlgL	1236	500, 1455
lpg1276	electron transferring flavoprotein dehydrogenase	1632	500, 1455
lpg1280	malate oxidoreductase	1725	500, 1455
lpg1283	lipoprotein NlpD	744	500, 1455
lpg1285	homogentisate 1,2-dioxygenase	1251	500, 1455
lpg1287	crossover junction endodeoxyribonuclease RuvC	525	500, 1455
lpg1296	protein involved in catabolism of external DNA	864	500, 1455
lpg1304	tryptophan synthetase, beta subunit	1200	500, 1455

lpg1319	type II secretory pathway protein E	1485	500, 1455
lpg1338	flagellar hook associated protein 2 FliD	1626	500, 1455
lpg1340	flagellin	1428	500, 1455
lpg1347	rare lipoprotein B	492	500, 1455
lpg1348	leucyl tRNA synthetase	2472	500, 1455
lpg1351	piperidine-6-carboxylate dehydrogenase	1521	500, 1455
lpg1358	general secretion pathway protein LspK	969	500, 1455
lpg1360	general secretion pathway protein LspI	378	500, 1455
lpg1364	glutamine synthetase, type I	1410	500, 1455
lpg1365	hypothetical protein	561	500, 1455
lpg1367	1-aminocyclopropane-1-carboxylate deaminase	903	500, 1455
lpg1374	rod shape determining protein RodA	1119	500, 1455
lpg1394	S-malonyl transferase	948	500, 1455
lpg1395	3-oxoacyl-(acyl carrier protein) reductase	747	500, 1455
lpg1399	thymidylate kinase	639	500, 1455
lpg1400	DNA polymerase III, delta prime subunit	906	500, 1455
lpg1402	deoxyribonuclease TatD	813	500, 1455
lpg1414	glycerol kinase	1476	500, 1455
lpg1420	cytidylate kinase	696	500, 1455
lpg1422	hypothetical membrane protein	291	500, 1455
lpg1424	aminotransferase	1116	500, 1455
lpg1425	orotidine 5'-phosphate decarboxylase PyrF	690	500, 1455
lpg1431	hypothetical protein	399	500, 1455
lpg1434	xanthosine phosphorylase	840	500, 1455
lpg1452	lipase A	888	500, 1455
lpg1464	hypothetical protein	159	500, 1455
lpg1469	Rtn protein	1599	500, 1455
lpg1485	hypothetical protein	402	500, 1455
lpg1486	AsnC family transcription regulator protein	522	500, 1455
lpg1507	sodium/hydrogen antiporter	1173	500, 1455
lpg1508	rare lipoprotein A	822	500, 1455
lpg1509	D-alanyl-D-alanine carboxypeptidase	1293	500, 1455
lpg1512	DedA/PAP2 domain protein	2037	500, 1455
lpg1514	lipoprotein	732	500, 1455
lpg1526	hypothetical protein	594	500, 1455
lpg1531	phenazine biosynthesis PhzF	792	500, 1455
lpg1537	transport protein	732	500, 1455
lpg1540	universal stress protein A	423	500, 1455
lpg1548	nucleoside diphosphate kinase	477	500, 1455
lpg1558	pyruvate dehydrogenase E1 alpha subunit	1095	500, 1455
lpg1562	mercuric reductase	2145	500, 1455
lpg1597	thiolase	1320	500, 1455
lpg1604	hypothetical protein	678	500, 1455
lpg1620	hypothetical protein	441	500, 1455
lpg1639	hypothetical protein	1317	500, 1455
lpg1641	acylaminoacyl peptidase	1980	500, 1455
lpg1644	hypothetical protein	765	500, 1455

lpg1646	cytochrome b561 transmembrane protein	531	500, 1455
lpg1659	membrane protein	1044	500, 1455
lpg1666	hypothetical protein	1404	500, 1455
lpg1669	alpha-amylase, putative	2226	500, 1455
lpg1672	phosphoribosylglycinamide formyltransferase	579	500, 1455
lpg1674	amidophosphoribosyltransferase	1500	500, 1455
lpg1680	thiol:disulfide interchange protein DsbD	1380	500, 1455
lpg1700	uracil DNA glycosylase	720	500, 1455
lpg1721	deaminase	426	500, 1455
lpg1722	GMP synthetase	1578	500, 1455
lpg1730	sn-glycerol-3-phosphate transmembrane ABC transporter	771	500, 1455
lpg1735	glutamyl/tRNA (Gln) amidotransferase, C subunit	303	500, 1455
lpg1736	glutamyl/tRNA (Gln) amidotransferase, A subunit	1452	500, 1455
lpg1746	cysteine desulfurase NifS	1164	500, 1455
lpg1748	inositol-1-monophosphatase	786	500, 1455
lpg1756	flagellar protein FliJ	456	500, 1455
lpg1761	flagellar hook-basal body protein FliE	315	500, 1455
lpg1763	sensor kinase HydH	1032	500, 1455
lpg1771	peptide maturation protein PmbA	1416	500, 1455
lpg1779	hypothetical protein	402	500, 1455
lpg1782	flagellar biosynthesis sigma factor FliA	789	500, 1455
lpg1785	flagellar biosynthetic protein FlhA	2079	500, 1455
lpg1789	flagellar biosynthetic protein FliP	750	500, 1455
lpg1791	flagellar motor switch protein FliN	330	500, 1455
lpg1798	hypothetical protein	1197	500, 1455
lpg1800	regulatory protein RecX	396	500, 1455
lpg1805	DNA mismatch repair protein MutS	2598	500, 1455
lpg1808	porphobilinogen synthase	996	500, 1455
lpg1809	hypothetical protein	393	500, 1455
lpg1810	long chain fatty acid transporter	1479	500, 1455
lpg1812	ATP-dependent DNA helicase (UvrD/Rep helicase)	3231	500, 1455
lpg1813	ATPase (Mrp)	1074	500, 1455
lpg1816	major facilitator family transporter	1290	500, 1455
lpg1824	acyl CoA dehydrogenase	1170	500, 1455
lpg1836	coiled coil domain protein	1419	500, 1455
lpg1839	glycyl tRNA synthetase, beta subunit	2067	500, 1455
lpg1845	lipoprotein VacJ-like	783	500, 1455
lpg1847	glutamate-cysteine ligase	1296	500, 1455
lpg1850	rhodanese domain protein	351	500, 1455
lpg1855	peptidyl prolyl cis-trans isomerase D	1875	500, 1455
lpg1874	general secretion pathway protein L	1140	500, 1455
lpg1882	lactoylglutathione lyase	441	500, 1455
lpg1893	major facilitator family transporter	1281	500, 1455
lpg1906	transporting ATPase	534	500, 1455
lpg1910	D-alanyl-D-alanine carboxypeptidase	1263	500, 1455
lpg1911	glutamate tRNA synthetase catalytic subunit	1485	500, 1455

lpg1917	amino acid antiporter	1422	500, 1455
lpg1918	hypothetical protein	1431	500, 1455
lpg1921	glycoprotease (O-sialoglycoprotein endopeptidase)	672	500, 1455
lpg1943	hypothetical protein	258	500, 1455
lpg1944	hypothetical protein	1047	500, 1455
lpg1945	3',5'-cyclic nucleotide phosphodiesterase	984	500, 1455
lpg1999	pterin 4 alpha carbinolamine dehydratase	342	500, 1455
lpg2002	transmembrane protein YajC, preprotein translocase subunit	336	500, 1455
lpg2014	pyridoxal-5'-phosphate dependent enzyme family	696	500, 1455
lpg2015	pyrroline-5-carboxylate reductase	789	500, 1455
lpg2025	chaperone protein DnaK, heat shock protein Hsp70	1950	500, 1455
lpg2036	Maf-like protein (septum formation)	603	500, 1455
lpg2037	enolase	1269	500, 1455
lpg2040	mevalonate diphosphate decarboxylase	969	500, 1455
lpg2046	ABC transporter, ATP binding protein	738	500, 1455
lpg2049	hypothetical protein	300	500, 1455
lpg2189	drug efflux protein	954	500, 1455
lpg2193	sulfate transporter	1554	500, 1455
lpg2200	hypothetical protein	537	500, 1455
lpg2201	replication factor C subunit (activator I)	1434	500, 1455
lpg2202	hypothetical protein	333	500, 1455
lpg2207	hypothetical protein	1224	500, 1455
lpg2208	zinc binding dehydrogenase	1014	500, 1455
lpg2214	nucleoside-diphosphate sugar epimerase	924	500, 1455
lpg2231	3-oxoacyl reductase	753	500, 1455
lpg2232	3-oxoacyl-(acyl carrier protein) synthase III FabH	1011	500, 1455
lpg2242	hypothetical protein	1326	500, 1455
lpg2243	uracil phosphoribosyltransferase	645	500, 1455
lpg2245	C4-dicarboxylate transport protein	1293	500, 1455
lpg2247	DedA family protein	756	500, 1455
lpg2250	alcohol dehydrogenase, iron containing	1161	500, 1455
lpg2259	periplasmic, osmotically inducible protein Y-like	312	500, 1455
lpg2273	glycerol-3-phosphate binding periplasmic protein	1314	500, 1455
lpg2300	ankyrin repeat domain protein	1404	500, 1455
lpg2303	chorismate synthase AroC	1059	500, 1455
lpg2304	adenine specific methylase	933	500, 1455
lpg2313	hypothetical protein	1293	500, 1455
lpg2316	3-hydroxybutyrate dehydrogenase	783	500, 1455
lpg2320	hypothetical protein	474	500, 1455
lpg2323	type II secretion system protein (twitching motility protein)	1122	500, 1455
lpg2325	hypothetical protein	837	500, 1455
lpg2336	peptide chain release factor 1 (RF-1)	1089	500, 1455
lpg2339	hypothetical protein	834	500, 1455
lpg2346	transcriptional regulator	939	500, 1455
lpg2350	alkylhydroperoxide reductase, AhpC/TSA family	639	500, 1455
lpg2355	amidase (enantiomer selective)	1410	500, 1455

lpg2356	transmembrane protein	834	500, 1455
lpg2358	30S ribosomal protein S21	240	500, 1455
lpg2359	hypothetical protein	444	500, 1455
lpg2393	bacterioferritin (cytochrome b1)	480	500, 1455
lpg2401	putative secreted esterase	1527	500, 1455
lpg2433	hypothetical protein	1761	500, 1455
lpg2439	NADPH-dependent FMN reductase domain protein	552	500, 1455
lpg2454	acetyltransferase, GNAT family, ElaA-like protein	447	500, 1455
lpg2457	two component response regulator	411	500, 1455
lpg2467	cytochrome c3 hydrogenase alpha chain	1293	500, 1455
lpg2468	sulfhydrogenase delta subunit	786	500, 1455
lpg2469	hydrogenase/sulfur reductase gamma subunit	846	500, 1455
lpg2493	small heat shock protein HspC2	495	500, 1455
lpg2506	sensor histidine kinase/response regulator LuxN	1263	500, 1455
lpg2507	hypothetical protein	696	500, 1455
lpg2515	structural toxin protein (hemagglutinin/hemolysin) RtxA	366	500, 1455
lpg2518	hypothetical protein	342	500, 1455
lpg2526	hypothetical protein	1368	500, 1455
lpg2530	3-deoxy-D-arabino-heptulosonate 7-phosphate (DAHP) synthase	1044	500, 1455
lpg2536	ferredoxin reductase	957	500, 1455
lpg2547	chaperonin CsaA	336	500, 1455
lpg2552	hypothetical protein	1668	500, 1455
lpg2577	hypothetical protein	759	500, 1455
lpg2581	2-oxoisovalerate dehydrogenase, E1 component, alpha and beta fusion	2271	500, 1455
lpg2586	cysteine protease, papain C1 family	1095	500, 1455
lpg2590	chromosome partitioning protein ParB (SpoOJ)	792	500, 1455
lpg2595	peptide deformylase	513	500, 1455
lpg2605	hypothetical protein	417	500, 1455
lpg2608	UDP-3-O-acyl-N-acetylglucosamine deacetylase	915	500, 1455
lpg2616	UDP-N-muramoylalanine-D-glutamate ligase	1344	500, 1455
lpg2625	carbamoyl phosphate synthase, large subunit	3204	500, 1455
lpg2630	permease	1002	500, 1455
lpg2631	aminopeptidase A/I	1485	500, 1455
lpg2634	leucine aminopeptidase	1365	500, 1455
lpg2635	integral membrane protein (putative virulence factor) MviN, possible role in motility	1572	500, 1455
lpg2643	hypothetical protein	660	500, 1455
lpg2650	50S ribosomal protein L27	279	500, 1455
lpg2655	sensory box protein, EAL domain, GGDEF domain, signal transduction protein	1116	500, 1455
lpg2660	transmembrane protein	663	500, 1455
lpg2662	pantoate-beta-alanine ligase	759	500, 1455
lpg2671	zinc protease (peptidase, M16 family)	1326	500, 1455
lpg2679	D-isomer specific 2-hydroxyacid dehydrogenase	945	500, 1455
lpg2680	UDP-N-acetylmuramyl tripeptide synthase	1029	500, 1455
lpg2700	sugar kinase	1482	500, 1455

lpg2702	stringent starvation protein A	621	500, 1455
lpg2703	ubiquinol-cytochrome c reductase, cytochrome c1	741	500, 1455
lpg2708	ferredoxin 2Fe-2S protein	216	500, 1455
lpg2710	phenylalanyl tRNA synthetase, beta subunit	2382	500, 1455
lpg2711	phenylalanyl tRNA synthetase, alpha subunit	1029	500, 1455
lpg2713	translational initiation factor IF-3	390	500, 1455
lpg2725	inner membrane protein	630	500, 1455
lpg2736	uroporphyrinogen III methylase	753	500, 1455
lpg2757	hypothetical protein	861	500, 1455
lpg2760	DNA-binding response regulator	729	500, 1455
lpg2762	hypothetical protein	717	500, 1455
lpg2765	HIT family hydrolase	342	500, 1455
lpg2772	initiation factor IF2-beta (IF-2 gamma, IF-2 alpha)	2607	500, 1455
lpg2778	NADH dehydrogenase I, L subunit	1974	500, 1455
lpg2782	NADH dehydrogenase I, H subunit	1023	500, 1455
lpg2785	NADH dehydrogenase I, E subunit	504	500, 1455
lpg2788	NADH dehydrogenase I, B subunit	477	500, 1455
lpg2794	phosphoglucomutase/phosphomannomutase MrsA	1368	500, 1455
lpg2805	peptide transport protein, POT family	1503	500, 1455
lpg2806	hypothetical protein	1383	500, 1455
lpg2808	shikimate-5-dehydrogenase	798	500, 1455
lpg2814	aminopeptidase	1269	500, 1455
lpg2819	tyrosine phosphatase II superfamily protein	960	500, 1455
lpg2824	DNA repair protein RecN	1668	500, 1455
lpg2848	ribonuclease, T2 family	1014	500, 1455
lpg2859	MoxR protein (ATPase) methanol dehydrogenase regulatory protein	996	500, 1455
lpg2860	hypothetical protein	477	500, 1455
lpg2861	nitrogen regulation protein	990	500, 1455
lpg2865	6-pyruvoyl tetrahydropterin synthase, putative	495	500, 1455
lpg2867	thioesterase	393	500, 1455
lpg2869	prolipoprotein diacylglyceryl transferase	771	500, 1455
lpg2874	hypothetical protein	885	500, 1455
lpg2880	endonuclease III	636	500, 1455
lpg2890	glucose inhibited division protein B	627	500, 1455
lpg2898	cytochrome c	1614	500, 1455
lpg2903	ubiquinone/menaquinone biosynthesis methyltransferase UbiE	753	500, 1455
lpg2904	hypothetical protein	624	500, 1455
lpg2908	peptide methionine sulfoxide reductase	870	500, 1455
lpg2925	outer membrane efflux protein	1629	500, 1455
lpg2927	hypothetical protein	1350	500, 1455
lpg2933	oxidoreductase, 3-octaprenyl-4-hydroxybenzoate carboxy-lyase	1467	500, 1455
lpg2934	transcription termination factor Rho	1272	500, 1455
lpg2935	RSc1188; probable thioredoxin 1	327	500, 1455
lpg2955	integration host factor beta subunit	312	500, 1455
lpg2957	stomatin like transmembrane protein	780	500, 1455

lpg2965	peroxynitrite reductase, AhpC/Tsa family	606	500, 1455
lpg2967	superoxide dismutase	591	500, 1455
lpg2969	hypothetical protein	786	500, 1455
lpg2975	hypothetical protein	2616	500, 1455
lpg2983	ATP synthase gamma chain, ATP synthase F1 gamma chain	867	500, 1455
lpg2987	ATP synthase F0, C subunit	276	500, 1455
lpg2994	hypothetical protein	357	500, 1455
lpg2997	alkane-1-monooxygenase	1167	500, 1455
lpg2999	astacin protease	801	500, 1455
lpg0002	DNA polymerase III beta chain	1104	1455
lpg0004	DNA gyrase subunit B	2421	1455
lpg0005	peptidylarginine deiminase	1047	1455
lpg0021	alpha helix protein	480	1455
lpg0022	hypothetical protein	2124	1455
lpg0023	transmembrane protein	543	1455
lpg0025	Rcp	573	1455
lpg0028	ubiquinone biosynthesis protein COQ7	714	1455
lpg0032	leucine aminopeptidase	1194	1455
lpg0035	hypothetical protein	357	1455
lpg0037	arginine 3rd transport system periplasmic binding protein	744	1455
lpg0040	integral membrane protein	996	1455
lpg0043	hypothetical protein	861	1455
lpg0047	chloramphenicol acetyltransferase	696	1455
lpg0048	acetyltransferase	879	1455
lpg0052	carboxyphosphoenolpyruvate phosphonomutase	894	1455
lpg0075	hypothetical protein	315	1455
lpg0076	hypothetical protein	582	1455
lpg0089	hypothetical protein	447	1455
lpg0091	conserved domain protein	465	1455
lpg0094	ribose-5-phosphate isomerase A	651	1455
lpg0095	cytosolic IMP-GMP specific 5'-nucleotidase	1380	1455
lpg0100	UDP-3-O-[3-hydroxymyristoyl] glucosamine N-acyltransferase	1071	1455
lpg0102	3-oxoacyl-(acyl carrier protein) synthase	1230	1455
lpg0105	cytochrome oxidase-like	597	1455
lpg0106	xanthine/uracil permease	1281	1455
lpg0110	hypothetical protein	1803	1455
lpg0111	squalene and phytoene synthases	1041	1455
lpg0115	hypothetical protein	309	1455
lpg0117	glycine cleavage system H protein	378	1455
lpg0119	hypothetical protein	480	1455
lpg0122	ABC transporter, ATP binding protein	1299	1455
lpg0125	GTP binding protein EngB	603	1455
lpg0129	methylmalonate-semialdehyde dehydrogenase	1527	1455
lpg0130	hypothetical protein	2469	1455
lpg0138	glyceraldehyde 3-phosphate dehydrogenase	1029	1455

lpg0140	hypothetical protein	1335	1455
lpg0153	hypothetical protein	315	1455
lpg0183	amine oxidase, flavin containing	1497	1455
lpg0188	acyl CoA transferase/carnitine dehydratase	1077	1455
lpg0194	catalase/(hydro)peroxidase KatG	2250	1455
lpg0197	hypothetical protein	318	1455
lpg0206	membrane protein	414	1455
lpg0209	hypothetical protein	1968	1455
lpg0213	inner membrane protein, LrgB family protein	708	1455
lpg0217	phosphoribosylaminoimidazole carboxylase, ATPase subunit	1080	1455
lpg0227	hypothetical protein	1107	1455
lpg0229	heme oxygenase	834	1455
lpg0238	glycine betaine aldehyde dehydrogenase	1467	1455
lpg0239	4-aminobutyrate aminotransferase	1353	1455
lpg0243	short chain dehydrogenase	600	1455
lpg0244	pyridine nucleotide-disulfide oxidoreductase	1395	1455
lpg0256	conserved domain protein	1047	1455
lpg0264	hypothetical protein	699	1455
lpg0267	magnesium and cobalt transport protein CorA	1053	1455
lpg0269	hypothetical protein	1563	1455
lpg0271	bifunctional pyrazinamidase/nicotinamidase	642	1455
lpg0276	Ras GEF	1500	1455
lpg0282	hypothetical protein	777	1455
lpg0295	mannose-1-phosphate guanyltransferase	663	1455
lpg0298	peptidyl-prolyl cis-trans isomerase D (SurA)	1341	1455
lpg0299	pyridoxal phosphate biosynthetic protein PdxA	975	1455
lpg0301	hypothetical protein	546	1455
lpg0319	50S ribosomal protein L1	696	1455
lpg0320	50S ribosomal protein L10	534	1455
lpg0321	50S ribosomal protein L7/L12	381	1455
lpg0322	DNA-directed RNA polymerase beta subunit	4107	1455
lpg0323	DNA-directed RNA polymerase beta' subunit	4248	1455
lpg0324	30S ribosomal protein S12	381	1455
lpg0325	30S ribosomal protein S7	528	1455
lpg0330	50S ribosomal protein L4	609	1455
lpg0332	50S ribosomal protein L2	828	1455
lpg0335	30S ribosomal protein S3	657	1455
lpg0336	50S ribosomal protein L16/(L10E)	414	1455
lpg0337	50S ribosomal subunit protein L29	195	1455
lpg0338	30S ribosomal protein S17	255	1455
lpg0339	50S ribosomal protein L14	366	1455
lpg0340	50S ribosomal protein L24	330	1455
lpg0341	50S ribosomal protein L5	561	1455
lpg0343	30S ribosomal protein S8	390	1455
lpg0347	50S ribosomal protein L30/(L7E)	186	1455
lpg0348	50S ribosomal protein L15	435	1455

lpg0349	preprotein translocase SecY	1335	1455
lpg0353	30S ribosomal protein S4	621	1455
lpg0355	50S ribosomal protein L17	384	1455
lpg0356	single strand binding protein	489	1455
lpg0357	major facilitator family transporter	1368	1455
lpg0359	acyl carrier protein	414	1455
lpg0361	3-oxoacyl-(acyl carrier protein) synthase II, C-terminal	1293	1455
lpg0363	lipid A biosynthesis acyltransferase	846	1455
lpg0365	hypothetical protein	2691	1455
lpg0366	diaminopimelate epimerase	834	1455
lpg0369	carboxylesterase/phospholipase	678	1455
lpg0370	oligoketide cyclase/lipid transporter protein	435	1455
lpg0371	hypothetical protein	273	1455
lpg0372	small protein A, tmRNA-binding	345	1455
lpg0374	hypothetical protein	387	1455
lpg0377	hypothetical protein	744	1455
lpg0380	hypothetical protein	720	1455
lpg0382	osmotically inducible protein Y	567	1455
lpg0384	excinuclease ABC A subunit	2856	1455
lpg0387	ABC transporter, permease protein	774	1455
lpg0391	SM20-related protein	540	1455
lpg0392	zinc metalloprotease	708	1455
lpg0393	hypothetical protein	864	1455
lpg0394	methylated DNA protein cysteine S-methyltransferase	456	1455
lpg0395	50S ribosomal protein L19	366	1455
lpg0396	tRNA (guanine N1) methyltransferase	771	1455
lpg0399	30S ribosomal protein S16	261	1455
lpg0400	signal recognition particle protein Ffh	1377	1455
lpg0404	amino acid antiporter	1404	1455
lpg0405	hypothetical protein	591	1455
lpg0406	hypothetical protein	342	1455
lpg0407	hypothetical protein	444	1455
lpg0413	hypothetical, SCO1/SenC family protein	642	1455
lpg0418	6-phosphogluconate dehydratase	1839	1455
lpg0421	D-xylose (galactose, arabinose)-proton symporter	1422	1455
lpg0422	glucoamylase	1350	1455
lpg0423	transcriptional regulator, cro family	237	1455
lpg0424	hypothetical protein	540	1455
lpg0425	ferrochelatase	999	1455
lpg0426	cold shock protein CspD	234	1455
lpg0428	glyoxylase domain hypothetical protein	429	1455
lpg0432	hypothetical protein	900	1455
lpg0433	hypothetical protein	381	1455
lpg0440	hypothetical protein	213	1455
lpg0442	IcmS	345	1455
lpg0443	IcmR	363	1455

lpg0444	IcmQ	600	1455
lpg0445	IcmP (DotM)	1143	1455
lpg0446	IcmO (DotL)	2352	1455
lpg0447	LphA (DotK)	570	1455
lpg0448	IcmM (DotJ)	285	1455
lpg0449	IcmL (DotI)	639	1455
lpg0450	IcmK (DotH)	1086	1455
lpg0452	IcmG (DotF)	810	1455
lpg0454	IcmD (DotP)	282	1455
lpg0455	IcmJ (DotN)	645	1455
lpg0457	TphA (ProP)	1257	1455
lpg0458	IcmF	2922	1455
lpg0459	IcmH (DotU)	786	1455
lpg0460	phosphoribosylamineimidazolecarboxamide formyltransferase	1590	1455
lpg0462	acetyl CoA carboxylase, biotin carboxylase subunit	1350	1455
lpg0468	lipase A	852	1455
lpg0469	endonuclease/exonuclease/phosphatase family protein	774	1455
lpg0471	phenol hydroxylase	747	1455
lpg0473	hypothetical protein	297	1455
lpg0475	sugar transport PTS system phosphocarrier HPr protein	270	1455
lpg0476	sigma-54 modulation protein	300	1455
lpg0478	50S ribosomal protein L33	165	1455
lpg0482	endo-1,4 beta-glucanase	1164	1455
lpg0491	amino acid (glutamine) ABC transporter, periplasmic amino acid binding protein	735	1455
lpg0498	leucine-, isoleucine-, valine-, threonine-, and alanine-binding protein	1179	1455
lpg0499	carboxy-terminal protease	1338	1455
lpg0500	peptidase, M23/M37 family	1170	1455
lpg0506	outer membrane protein	2361	1455
lpg0507	outer membrane protein OmpH	501	1455
lpg0510	(3R)-hydroxymyristoyl-(acyl carrier protein) dehydratase	453	1455
lpg0511	acyl-(acyl carrier protein)-UDP-N-acetylglucosamine acyltransferase	771	1455
lpg0512	CrcB protein, camphor resistance	405	1455
lpg0513	seryl tRNA synthetase	1281	1455
lpg0528	succinate dehydrogenase cytochrome b556 subunit C	393	1455
lpg0530	succinate dehydrogenase flavoprotein subunit A	1770	1455
lpg0534	succinyl CoA synthetase beta chain	1221	1455
lpg0535	succinyl CoA synthetase alpha chain	876	1455
lpg0539	hypothetical protein	390	1455
lpg0542	DNA binding protein Fis	282	1455
lpg0547	outer membrane lipoprotein LolB	600	1455
lpg0548	phosphopantetheine adenylyltransferase	600	1455
lpg0552	suppressor of GroEL (SugE)	321	1455
lpg0556	hypothetical protein	621	1455

lpg0558	stearoyl-CoA-9-desaturase	1188	1455
lpg0560	acetyoacetyl CoA reductase	747	1455
lpg0561	acetyoacetyl CoA reductase	747	1455
lpg0562	hypothetical protein	399	1455
lpg0563	hypothetical protein	357	1455
lpg0566	spore maturation protein B	534	1455
lpg0568	tyrosyl tRNA synthetase	1272	1455
lpg0577	transferase	537	1455
lpg0583	phosphate transporter	1254	1455
lpg0584	hypothetical phosphate transport regulator	672	1455
lpg0585	hypothetical protein	765	1455
lpg0587	YqgF	429	1455
lpg0588	aspartate carbamoyltransferase	894	1455
lpg0591	hypothetical protein	261	1455
lpg0592	nitrogen regulatory P-II transcription regulator	375	1455
lpg0594	hypothetical protein	186	1455
lpg0602	ATP transporter, ABC binding component, ATP-binding protein	753	1455
lpg0604	aminotransferase	1245	1455
lpg0605	nitrogen fixation protein (Fe-S cluster formation) NifU	450	1455
lpg0608	hypothetical SAM-dependent methyltransferase	915	1455
lpg0612	alcohol dehydrogenase (NADP-dependent, zinc-type)	1047	1455
lpg0614	hypothetical protein	405	1455
lpg0616	GTP cyclohydrolase I PLUS perhaps regulatory protein	1251	1455
lpg0624	hypothetical protein	378	1455
lpg0626	DNA uptake/competence protein ComA	2208	1455
lpg0627	type IV pilin	450	1455
lpg0630	type IV fimbrial biogenesis PilW related protein, transmembrane)	1068	1455
lpg0640	heat shock protein, HslVU, proteasome-related peptidase subunit	549	1455
lpg0641	ATP dependent Hsl protease, ATP binding subunit	1344	1455
lpg0643	ribonuclease BN	1239	1455
lpg0651	malate oxidoreductase	1236	1455
lpg0652	major facilitator family transporter	1293	1455
lpg0654	DNA adenine methylase	819	1455
lpg0656	tryptophan/tyrosine permease	1191	1455
lpg0657	outer membrane protein, OmpA family protein	750	1455
lpg0658	HlyD family secretion protein	858	1455
lpg0659	ABC transporter ElsE	1743	1455
lpg0660	ABC transporter permease protein	1122	1455
lpg0663	soluble lytic murein transglycosylase	1821	1455
lpg0665	putative transmembrane protein	465	1455
lpg0672	acetoacetate decarboxylase ADC	765	1455
lpg0673	signal peptide protein	273	1455
lpg0674	adenylate cyclase	1314	1455
lpg0677	hypothetical protein	267	1455

lpg0678	arginine ABC transporter, periplasmic binding protein	765	1455
lpg0679	adenylyl transferase	2742	1455
lpg0680	dipeptidyl aminopeptidase/acylaminoacyl peptidase	1269	1455
lpg0685	Fe-S oxidoreductase	1308	1455
lpg0687	Hsp10, 10 kDa chaperonin GroES	291	1455
lpg0688	Hsp60, 60K heat shock protein HtpB	1653	1455
lpg0692	ABC type dipeptide/oligopeptide/nickel transport, ATPase component	1821	1455
lpg0698	hypothetical protein	852	1455
lpg0699	outer membrane protein TolC	1368	1455
lpg0704	enhanced entry protein EnhA	612	1455
lpg0712	endo-1,4-beta-xylanase-like	696	1455
lpg0716	hypothetical protein	1014	1455
lpg0719	valyl tRNA synthase	2766	1455
lpg0720	multidrug resistance protein	3048	1455
lpg0721	RND efflux membrane fusion protein, acriflavin resistance protein E	1263	1455
lpg0722	hypothetical protein	405	1455
lpg0723	hypothetical, His rich	438	1455
lpg0724	hypothetical periplasmic or secreted lipoprotein	312	1455
lpg0725	serine hydroxymethyltransferase	1254	1455
lpg0730	transmembrane permease	1053	1455
lpg0732	hypothetical protein	639	1455
lpg0734	glutamine dependent NAD+ synthetase	1611	1455
lpg0737	hypothetical signal peptide protein	435	1455
lpg0738	replicative DNA helicase	1383	1455
lpg0739	alanine racemase	1074	1455
lpg0740	17kDa common antigen	450	1455
lpg0741	hypothetical protein	525	1455
lpg0742	hypothetical protein	1254	1455
lpg0747	hypothetical protein	819	1455
lpg0749	imidazole glycerol phosphate synthase, cyclase subunit HisF	765	1455
lpg0752	N-acetylneuraminic acid synthetase	1071	1455
lpg0753	polysialic acid biosynthesis	1134	1455
lpg0754	acetyltransferase	609	1455
lpg0755	pyridoxal phosphate-dependent enzyme apparently involved in regulation of cell wall biosynthesis	1503	1455
lpg0759	glucose-6-phosphate isomerase	1503	1455
lpg0781	global regulator (carbon storage regulator)	249	1455
lpg0786	cell cycle protein MesJ	1302	1455
lpg0791	macrophage infectivity potentiator (Mip)	708	1455
lpg0802	sulfate transporter	1704	1455
lpg0804	choloylglycine hydrolase	999	1455
lpg0810	hypothetical protein	318	1455
lpg0811	rod shape determining protein MreB	1044	1455
lpg0815	hypothetical protein	699	1455
lpg0816	isocitrate dehydrogenase, NADP-dependent	1269	1455

lpg0818	ATP binding protease component ClpA	2274	1455
lpg0826	exonuclease VII, large subunit	1332	1455
lpg0829	two component histidine kinase, GGDEF domain protein/EAL domain protein	1920	1455
lpg0833	indole-3-glycerol phosphate synthase	777	1455
lpg0835	anthranilate synthase component II	579	1455
lpg0836	ABC transporter, ATP binding protein	726	1455
lpg0837	hypothetical protein	510	1455
lpg0838	hypothetical protein	570	1455
lpg0842	toluene tolerance protein Ttg2B	783	1455
lpg0843	toluene tolerance protein Ttg2C	477	1455
lpg0845	hypothetical protein	282	1455
lpg0846	hypothetical BolA like protein	246	1455
lpg0847	UDP-N-acetylglucosamine 1-carboxyvinyltransferase	1269	1455
lpg0848	hypothetical TIGR00486	759	1455
lpg0851	membrane fusion protein	1017	1455
lpg0852	hypothetical protein	594	1455
lpg0856	heme exporter protein CcmA	741	1455
lpg0858	heme exporter protein CcmC	792	1455
lpg0859	cytochrome c-type biogenesis protein CcmD	132	1455
lpg0860	cytochrome c-type biogenesis protein CcmE	432	1455
lpg0862	thiol:disulfide interchange protein DsbE	534	1455
lpg0867	ATP-dependent DNA helicase RecQ	1827	1455
lpg0869	3-hydroxyisobutyryl Coenzyme A hydrolase	780	1455
lpg0872	peptide chain release factor 3	1581	1455
lpg0874	NAD(P) transhydrogenase	1422	1455
lpg0875	transmembrane NAD(P) transhydrogenase	297	1455
lpg0877	hypothetical transporter	555	1455
lpg0878	hypothetical protein	300	1455
lpg0882	hypothetical protein	435	1455
lpg0887	N-succinyl-diaminopimelate desuccinylase	1134	1455
lpg0888	2,3,4,5-tetrahydropyridine-2-carboxylate N-succinyltransferase DapD	831	1455
lpg0889	1-acyl-sn-glycerol-3-phosphate acyltransferase	894	1455
lpg0892	kynurenine 3-monooxygenase	1350	1455
lpg0896	hypothetical protein	378	1455
lpg0897	Na/Ca antiporter	960	1455
lpg0899	A/G specific adenine glycosylase	1068	1455
lpg0900	hypothetical protein	1569	1455
lpg0902	hypothetical protein	903	1455
lpg0904	hydrolase, isochorismatase family	546	1455
lpg0905	3-oxoacyl-(acyl carrier protein) reductase	744	1455
lpg0906	flagellar biosynthesis/type III secretory pathway chaperone	498	1455
lpg0907	negative regulator of flagellin synthesis	321	1455
lpg0908	flagella basal body P-ring formation protein FlgA	702	1455
lpg0909	cytochrome c5	408	1455
lpg0910	enhanced entry protein EnhA	558	1455

lpg0915	cell division transmembrane protein FtsL	279	1455
lpg0917	UDP-N-acetylmuramyl-tripeptide synthetase MurE	1452	1455
lpg0918	erythronate-4-phosphate dehydrogenase	1053	1455
lpg0920	phosphatidylglycerophosphatase B	648	1455
lpg0925	penicillin binding protein 1A	2385	1455
lpg0926	hypothetical protein	1011	1455
lpg0927	type IV pilus biogenesis protein PilM	1065	1455
lpg0928	type IV pilus biogenesis protein PilN	549	1455
lpg0929	type IV pilus biogenesis protein PilO	636	1455
lpg0932	shikimate kinase	528	1455
lpg0933	3-dehydroquinate synthetase	1110	1455
lpg0934	DamX-related protein	1452	1455
lpg0935	universal stress protein A (UspA)	432	1455
lpg0937	isoleucyl tRNA synthetase	2796	1455
lpg0938	lipoprotein signal peptidase	366	1455
lpg0940	LidA	2190	1455
lpg0941	hypothetical protein	3126	1455
lpg0942	GTP-binding protein Era	936	1455
lpg0943	DNA repair protein RecO	690	1455
lpg0946	pyridoxal phosphate biosynthetic protein PdxJ	807	1455
lpg0949	carrier/transport protein	675	1455
lpg0951	TldD protein	1443	1455
lpg0953	AMP-binding protein	1668	1455
lpg0955	transmembrane protein	1263	1455
lpg0956	hypothetical protein	1179	1455
lpg0960	peptide ABC transporter, permease protein	978	1455
lpg0961	peptide ABC transporter, permease protein	1374	1455
lpg0962	DNA polymerase III, alpha subunit	3447	1455
lpg0966	nucleoside-diphosphate sugar epimerases	1878	1455
lpg0970	amino acid permeases	1464	1455
lpg1117	hypothetical protein	474	1455
lpg1119	major acid phosphatase	1065	1455
lpg1122	membrane bound lytic murein transglycosylase D	1329	1455
lpg1131	cyclopropane fatty acid synthase	1167	1455
lpg1135	bacterial regulatory proteins, TetR family	612	1455
lpg1136	hypothetical protein	903	1455
lpg1137	hypothetical protein	969	1455
lpg1138	spermidine/putrescine-binding periplasmic protein PotD	1023	1455
lpg1144	hypothetical protein	507	1455
lpg1146	thermostable carboxypeptidase 1	1482	1455
lpg1147	hypothetical protein	504	1455
lpg1148	hypothetical protein	1512	1455
lpg1155	pyruvate decarboxylase	1680	1455
lpg1161	phosphoribosyltransferase	663	1455
lpg1164	acetylornithine deacetylase	1155	1455
lpg1165	uridine kinase	798	1455

lpg1166	hypothetical protein	2013	1455
lpg1167	hypothetical protein	522	1455
lpg1171	hypothetical protein	420	1455
lpg1172	TPR repeat protein	1488	1455
lpg1174	two component response regulator PilR	1329	1455
lpg1176	Zn-dependent protease	1449	1455
lpg1178	riboflavin synthase, alpha subunit RibE	615	1455
lpg1186	competence lipoprotein ComL	783	1455
lpg1188	Kup system potassium uptake protein	1896	1455
lpg1189	hypothetical protein	1002	1455
lpg1190	SAM-dependent methyltransferase	1173	1455
lpg1191	glycosyl hydrolase family 3	1188	1455
lpg1195	phosphoribosylformimino-5-aminoimidazole carboxamide ribotide isomerase	720	1455
lpg1196	amidotransferase HisH	600	1455
lpg1197	histidinol phosphatase and imidazoleglycerol-phosphate dehydratase = bifunctional protein HisB	1059	1455
lpg1203	cytochrome D ubiquinol oxidase, subunit II	1137	1455
lpg1205	cold shock domain family protein CspA	240	1455
lpg1206	sigma 54 modulation protein YhbH	573	1455
lpg1207	hypothetical protein	456	1455
lpg1208	transcriptional regulator MarR family	420	1455
lpg1212	IAA acetyltransferase/MarR transcriptional regulatory protein	972	1455
lpg1214	2-acylglycerophosphoethanolamine acyltransferase	1308	1455
lpg1215	oxygen-dependent coproporphyrinogen III oxidase	951	1455
lpg1216	flagellar basal body rod protein FlgB	393	1455
lpg1217	flagellar basal body rod protein FlgC	423	1455
lpg1220	flagellar basal body rod protein FlgF	747	1455
lpg1221	flagellar basal body rod protein FlgG	786	1455
lpg1277	ABC transporter ATP binding protein	1827	1455
lpg1278	hypothetical protein	318	1455
lpg1279	hypothetical protein	372	1455
lpg1281	hypothetical protein	384	1455
lpg1282	stationary phase surival protein SurE	756	1455
lpg1284	stationary phase specific sigma factor RpoS	1080	1455
lpg1286	YebC	744	1455
lpg1288	Holliday junction DNA helicase RuvA	600	1455
lpg1291	two component sensor kinase	1416	1455
lpg1292	DNA-binding response regulator	678	1455
lpg1293	intracellular septation protein A	546	1455
lpg1294	membrane bound lytic murein transglycosylase D	1440	1455
lpg1297	5,10-methylenetetrahydrofolate dehydrogenase	855	1455
lpg1300	integral membrane protein	468	1455
lpg1301	oxidoreductase	1299	1455
lpg1303	phosphoribosyl anthranilate isomerase	624	1455
lpg1305	tryptophan synthetase, alpha chain TrpA	819	1455
lpg1306	glutaminyl-tRNA synthetase	1656	1455

lpg1307	cysteinyl-tRNA synthetase	1371	1455
lpg1320	type II protein secretion LspD	2376	1455
lpg1324	multidrug resistance efflux pump	1215	1455
lpg1331	protease DO	1401	1455
lpg1332	hypothetical protein	726	1455
lpg1333	ribosomal large subunit pseudouridine synthase D, RluD	966	1455
lpg1334	tRNA thiotransferase	1344	1455
lpg1336	enhanced entry protein EnhA	747	1455
lpg1337	flagellar protein FliS	411	1455
lpg1339	hypothetical protein	282	1455
lpg1341	acetyl CoA carboxylase, carboxyltransferase, beta subunit	885	1455
lpg1342	FolC bifunctional protein	1287	1455
lpg1344	colicin V	534	1455
lpg1346	DNA polymerase III, delta subunit	1026	1455
lpg1349	apolipoprotein N-acyltransferase	1536	1455
lpg1350	L-lysine dehydrogenase	1137	1455
lpg1352	3-hydroxyacyl CoA dehydrogenase oxidoreductase protein/	2370	1455
lpg1359	general secretion pathway protein LspJ	618	1455
lpg1363	type II protein secretion LspF	1200	1455
lpg1369	chaperone Hsp90 HtpG	1872	1455
lpg1370	hypothetical protein	297	1455
lpg1372	oxidoreductase	963	1455
lpg1373	ribonuclease HII	576	1455
lpg1375	penicillin binding protein 2	1881	1455
lpg1376	hypothetical protein	471	1455
lpg1377	hypothetical protein	339	1455
lpg1385	hypothetical protein	378	1455
lpg1388	hypothetical protein	393	1455
lpg1391	50S ribosomal protein L32	192	1455
lpg1392	fatty acid/phospholipid synthesis protein PlsX	1029	1455
lpg1397	beta-ketoacyl-acyl carrier protein synthase II	1239	1455
lpg1398	periplasmic solute-binding protein	999	1455
lpg1401	type 4 fimbrial biogenesis protein PilZ	342	1455
lpg1403	hypothetical protein	1101	1455
lpg1404	major facilitator family transporter	1293	1455
lpg1405	multidrug translocase MdfA, chloramphenicol resistance pump Cmr	1281	1455
lpg1406	glycosyltransferase	1176	1455
lpg1408	choline kinase	1152	1455
lpg1409	hypothetical protein	771	1455
lpg1410	transcriptional regulatory protein	597	1455
lpg1411	adenylate kinase	711	1455
lpg1415	citrate synthase	1272	1455
lpg1416	purine nucleoside phosphorylase II	882	1455
lpg1417	DNA gyrase, A subunit	2616	1455
lpg1419	3-phosphoshikimate 1-carboxyvinyltransferase	1302	1455

lpg1421	30S ribosomal protein S1	1740	1455
lpg1429	hypothetical protein	537	1455
lpg1430	4-hydroxybenzoate octaprenyltransferase UbiA	849	1455
lpg1432	FAD linked oxidase	1797	1455
lpg1435	cytidine deaminase	396	1455
lpg1441	phosphate starvation-inducible protein PhoH	951	1455
lpg1444	tryptophanyl tRNA synthetase	1218	1455
lpg1445	hypothetical protein	792	1455
lpg1446	hypothetical protein	591	1455
lpg1447	pseudouridine synthase	747	1455
lpg1451	hypothetical protein	312	1455
lpg1453	hypothetical protein	519	1455
lpg1455	phospholipase C	1257	1455
lpg1456	23S rRNA (uracil-5-)methyltransferase RumA	1335	1455
lpg1459	aspartate aminotransferase	1194	1455
lpg1460	hypothetical protein	810	1455
lpg1461	single stranded DNA specific exonuclease RecJ	1740	1455
lpg1462	zinc binding TIM barrel protein, YjbN family	1005	1455
lpg1463	preprotein translocase; secretion protein SecA	2709	1455
lpg1466	hypothetical protein	747	1455
lpg1472	biotin synthase BioB	948	1455
lpg1473	8-amino-7-oxononanoate synthase	1146	1455
lpg1474	biotin biosynthesis protein BioH	720	1455
lpg1475	dethiobiotin synthetase	639	1455
lpg1476	hypothetical protein	315	1455
lpg1477	transmembrane protein	630	1455
lpg1482	hypothetical protein	837	1455
lpg1483	serine/threonine-protein kinase	1590	1455
lpg1484	hypothetical protein	810	1455
lpg1487	acetyltransferase, GNAT family	531	1455
lpg1502	dihydrolipoamide dehydrogenase	1440	1455
lpg1504	pyruvate dehydrogenase E1 component oxidoreductase protein AceE	2682	1455
lpg1505	hypothetical protein	390	1455
lpg1506	inner membrane protein AmpE	780	1455
lpg1511	lipoate-protein ligase B	600	1455
lpg1513	type I secretion system LssZ	615	1455
lpg1517	HlyD family secretion protein	1137	1455
lpg1519	purine/pyrimidine phosphoribosyltransferase	570	1455
lpg1520	hypothetical protein	327	1455
lpg1524	type 4 (IV) prepilin-like protein leader peptide processing enzyme PilD	870	1455
lpg1527	hypothetical protein	456	1455
lpg1529	2-methylcitrate dehydratase PrpD	1473	1455
lpg1530	2-methylcitrate synthase	1119	1455
lpg1535	rubredoxin (rubredoxin-type Fe(Cys)4 protein)	177	1455
lpg1536	transmembrane protein	423	1455
lpg1539	2-amino-4-hydroxy-6-	426	1455

	hydroxymethyldihydropteridine pyrophosphokinase FolK		
lpg1541	GTP-binding protein EngA	1389	1455
lpg1542	PQQ (pyrrolo quinoline) WD40-like repeat, enzyme repeat domain protein	1164	1455
lpg1545	DNA-binding protein, putative	570	1455
lpg1546	fimbrial biogenesis and twitching motility protein PilF	783	1455
lpg1547	radical SAM enzyme, Cfr family	1161	1455
lpg1549	hypothetical protein	885	1455
lpg1550	tRNA-(ms(2)io(6)a)-hydrolase(tRNA hydroxylase)	636	1455
lpg1553	septum site determining protein MinC	711	1455
lpg1554	long chain fatty acid-CoA ligase	1710	1455
lpg1559	pyruvate dehydrogenase E1 beta subunit	975	1455
lpg1564	integral membrane protein	744	1455
lpg1566	thiamine biosynthesis oxidoreductase ThiO	1071	1455
lpg1567	thiamine (thiazole) biosynthesis protein ThiG	792	1455
lpg1568	phosphomethylpyrimidine kinase ThiD/thiamin-phosphate pyrophosphorylase fused protein ThiE	1488	1455
lpg1573	biopolymer transport protein TolR	456	1455
lpg1575	esterase	396	1455
lpg1577	RNA polymerase sigma E factor RpoE	564	1455
lpg1578	hypothetical protein	450	1455
lpg1579	glycine cleavage T protein	1059	1455
lpg1580	cytochrome b-561 transmembrane protein	549	1455
lpg1582	hypothetical protein	441	1455
lpg1584	(CDP-alcohol) phosphatidyltransferase	768	1455
lpg1585	hypothetical protein	489	1455
lpg1587	hypothetical thiol-disulfide isomerase and thioredoxins family	558	1455
lpg1589	50S ribosomal protein L9	450	1455
lpg1592	30S ribosomal protein S6	339	1455
lpg1593	carbon storage regulator CsrA	198	1455
lpg1595	hypothetical protein	735	1455
lpg1596	enoyl CoA hydratase	2019	1455
lpg1605	hypothetical protein	480	1455
lpg1612	transcriptional regulator SkgA, mercury resistance	753	1455
lpg1618	beta-lactamase AmpS	843	1455
lpg1623	hydrogenase	1032	1455
lpg1624	alpha/beta hydrolase	984	1455
lpg1636	acetyltransferase, GNAT family	849	1455
lpg1638	drug:proton antiporter	1314	1455
lpg1640	transmembrane protein	558	1455
lpg1645	hypothetical protein	576	1455
lpg1650	myo-inositol catabolism protein IolD	1872	1455
lpg1653	D-xylose-proton symporter	1416	1455
lpg1656	hypothetical protein	954	1455
lpg1657	NG,NG-dimethylarginine dimethylaminohydrolase	768	1455
lpg1661	hypothetical protein	1119	1455

lpg1662	putative transport protein	597	1455
lpg1663	hypothetical protein	507	1455
lpg1667	hypothetical protein	1392	1455
lpg1679	hypothetical protein	714	1455
lpg1682	oxidoreductase, short chain dehydrogenase/reductase family	852	1455
lpg1696	proline dehydrogenase/delta-1-pyrroline-5-carboxylate dehydrogenase = bifunctional PutA protein	3165	1455
lpg1697	hypothetical protein	678	1455
lpg1698	ProQ-like, activator of ProP osmoprotectant transporter	372	1455
lpg1699	3-demethylubiquinone-9 3-methyltransferase UbiG	693	1455
lpg1701	kinectin 1 (kinesin receptor)	1683	1455
lpg1705	carboxypeptidase G2	1224	1455
lpg1706	arginine/ornithine succinyltransferase	1044	1455
lpg1707	succinylglutamic-5-semialdehyde dehydrogenase	1491	1455
lpg1710	hypothetical protein	366	1455
lpg1711	ribosome recycling factor	558	1455
lpg1712	uridylate kinase	744	1455
lpg1713	translation elongation factor Ts (EF-Ts)	900	1455
lpg1714	30S ribosomal protein S2	816	1455
lpg1720	protein-PII uridylyltransferase	2586	1455
lpg1723	inosine-5'-monophosphate dehydrogenase	1473	1455
lpg1724	septum site-determining protein MinD	831	1455
lpg1725	similar to cell division inhibitor MinE, putative pseudogene	221	1455
lpg1727	hydrolase	786	1455
lpg1731	sn-glycerol-3-phosphate transmembrane ABC transporter	879	1455
lpg1732	quinone oxidoreductase	1002	1455
lpg1733	chloride channel protein EriC (voltage gated)	1284	1455
lpg1734	anthranilate synthase (glutamine amidotransferase) component I	2157	1455
lpg1743	Fis transcriptional activator	288	1455
lpg1747	RNA methyltransferase	774	1455
lpg1749	signal peptide peptidase	957	1455
lpg1750	ClpB protein	2577	1455
lpg1751	hypothetical protein	1311	1455
lpg1752	hypothetical protein	648	1455
lpg1753	UDP-N-acetylmuramate:L-alanyl-gamma-D-glutamyl-meso-diaminopimelate ligase	1368	1455
lpg1754	hypothetical protein	621	1455
lpg1755	transmembrane protein	3822	1455
lpg1757	nucleotide binding protein FliI	1353	1455
lpg1758	flagellar assembly protein FliH	639	1455
lpg1762	sigma 54-dependent response regulator	1362	1455
lpg1764	ATPase associated with chromosome architecture	1305	1455
lpg1765	outer membrane lipoprotein carrier protein	615	1455
lpg1766	cell division protein FtsK	2385	1455

lpg1767	thioredoxin reductase	1050	1455
lpg1768	leucyl/phenylalanyl-tRNA protein transferase	669	1455
lpg1770	translation initiation factor IF-1	222	1455
lpg1778	peptide chain release factor 2 (RF-2)	1008	1455
lpg1788	flagellar biosynthetic protein FliQ	270	1455
lpg1792	flagellar protein	1068	1455
lpg1793	hypothetical protein	255	1455
lpg1803	hypothetical protein	936	1455
lpg1804	hypothetical 17.2kDa protein, CinA-related competence damage protein	495	1455
lpg1806	outer membrane protein	1689	1455
lpg1807	periplasmic protein	2541	1455
lpg1814	hypothetical protein	555	1455
lpg1815	hydrogen peroxide-inducible genes activator OxyR	891	1455
lpg1821	dihydroorotate oxidase	1167	1455
lpg1823	hypothetical protein	687	1455
lpg1825	acyl CoA C-acetyltransferase	1185	1455
lpg1826	hypothetical protein	321	1455
lpg1830	hydroxymethylglutaryl-CoA lyase	909	1455
lpg1831	acetoacetyl CoA synthetase	2001	1455
lpg1832	hypothetical protein	417	1455
lpg1833	D-methionine transport ATP binding protein MetN	1101	1455
lpg1834	ATP binding protein, permease protein	648	1455
lpg1835	29 kDa immunogenic protein	792	1455
lpg1837	SAM-dependent methyltransferase	732	1455
lpg1838	histidinol phosphate phosphatase	531	1455
lpg1840	glycyl tRNA synthetase, alpha subunit	924	1455
lpg1841	27 kDa outer membrane protein	786	1455
lpg1842	DNA dependent ATPase I and helicase II	2193	1455
lpg1843	proline iminopeptidase	960	1455
lpg1846	glutathione synthetase	963	1455
lpg1849	hypothetical protein	282	1455
lpg1851	hypothetical protein	663	1455
lpg1854	enoyl reductase	807	1455
lpg1858	HupB DNA binding protein HU-beta	303	1455
lpg1859	ATP-dependent protease La	2451	1455
lpg1860	ATP-dependent Clp protease, ATP binding subunit ClpX	1281	1455
lpg1861	ATP-dependent Clp protease, proteolytic subunit ClpP	645	1455
lpg1870	transmembrane protein	393	1455
lpg1871	signal peptidase I (lepB-1)	783	1455
lpg1873	membrane bound lytic murein transglycosylase	1044	1455
lpg1883	transmembrane protein	528	1455
lpg1887	hypothetical protein	354	1455
lpg1888	hypothetical protein	1332	1455
lpg1889	lipase	966	1455
lpg1891	hypothetical protein HI1736	303	1455

lpg1892	hypothetical protein	384	1455
lpg1894	chloride channel protein (voltage gated)	1314	1455
lpg1895	hypothetical protein	525	1455
lpg1896	hypothetical protein	480	1455
lpg1904	integral membrane protein	903	1455
lpg1905	ectonucleoside triphosphate diphosphohydrolase I	1182	1455
lpg1908	glutathione S-transferase	612	1455
lpg1913	6-phosphofructokinase	1245	1455
lpg1915	Tfp pilus assembly protein, major type IV pilin class A	423	1455
lpg1919	3-deoxy-manno-octulosonate cytidylyltransferase	753	1455
lpg1920	tetraacyldisaccharide-1-P-4'-kinase	180	1455
lpg1924	hypothetical protein	2793	1455
lpg1927	hypothetical protein	270	1455
lpg1942	3-hydroxyacyl CoA dehydrogenase	855	1455
lpg1949	hypothetical protein	1341	1455
lpg1993	polysaccharide deacetylase	879	1455
lpg1994	(outer) membrane bound lytic murein transglycosylase family protein	1194	1455
lpg2000	protein export protein SecF	918	1455
lpg2001	protein export protein SecD	1857	1455
lpg2004	S-adenosylmethionine:tRNA ribosyltransferase-isomerase	1050	1455
lpg2007	aspartyl protease	492	1455
lpg2009	guanosine-3, 5-bis(diphosphate)-3-pyrophosphohydrolase	2148	1455
lpg2010	guanylate kinase	630	1455
lpg2011	stress-induced protein	867	1455
lpg2012	ribonuclease PH	708	1455
lpg2013	twitching motility protein PilT	1035	1455
lpg2017	hypothetical protein	615	1455
lpg2018	hypothetical protein	282	1455
lpg2020	transcriptional regulator OruR, AraC family	1014	1455
lpg2021	adenosylhomocysteinase	1326	1455
lpg2023	carbamoyl phosphate synthase, small subunit	1125	1455
lpg2024	heat shock protein DnaJ, chaperone protein	1140	1455
lpg2027	2-keto-3-deoxy-D-arabino-heptulosonate 7-phosphate synthase	1338	1455
lpg2028	uroporphyrinogen decarboxylase	1062	1455
lpg2029	dihydroneopterin aldolase FolB, putative kinase	339	1455
lpg2031	arginyl tRNA synthetase	1770	1455
lpg2032	transporter, permease	1137	1455
lpg2033	ATP dependent DNA helicase RecG	2073	1455
lpg2034	cation efflux family protein	1164	1455
lpg2038	transmembrane protein	270	1455
lpg2039	putative mevalonate kinase	882	1455
lpg2041	radical activating enzyme	654	1455
lpg2042	outer membrane protein	969	1455
lpg2043	peptidoglycan associated lipoprotein	531	1455

lpg2044	conserved domain protein	603	1455
lpg2045	ABC transport system periplasmic substrate binding protein	924	1455
lpg2047	ABC transporter, permease	1125	1455
lpg2048	hypothetical protein	852	1455
lpg2051	isopentenyl-diphosphate delta-isomerase	1029	1455
lpg2052	hydroxymethylglutaryl CoA reductase	1299	1455
lpg2175	(2-pyrone-4,6-)dicarboxylic acid hydrolase	768	1455
lpg2176	sphingosine-1-phosphate lyase I	1827	1455
lpg2178	probable multidrug-efflux system transmembrane protein	3156	1455
lpg2186	polyketide synthase, type I	11343	1455
lpg2187	hypothetical protein	402	1455
lpg2194	(beta)-carbonic anhydrase	627	1455
lpg2203	alginate O-acetylation protein AlgJ	1560	1455
lpg2204	alginate O-acetylation protein	1422	1455
lpg2206	hypothetical protein	1101	1455
lpg2210	hypothetical protein	1050	1455
lpg2211	hypothetical protein	405	1455
lpg2212	acetylpolyamine aminohydolase	1281	1455
lpg2213	hemin binding protein Hbp	453	1455
lpg2220	hypothetical protein	1500	1455
lpg2222	TPR repeat protein, protein-protein interaction	1128	1455
lpg2225	expressed protein (GH3 homolog)	1530	1455
lpg2228	3-oxoacyl-(acyl carrier protein) synthase III	1062	1455
lpg2233	acyl carrier protein	228	1455
lpg2234	multidrug resistance protein D	1368	1455
lpg2235	sterol desaturase	1203	1455
lpg2238	transmembrane protein	387	1455
lpg2240	dipeptidyl aminopeptidase/acylaminoacyl peptidase	1209	1455
lpg2246	hypothetical protein	510	1455
lpg2248	hypothetical protein	2235	1455
lpg2249	glutamine amidotransferase, class I	699	1455
lpg2255	hypothetical protein	252	1455
lpg2256	metallo-beta-lactamase superfamily protein	1419	1455
lpg2258	hypothetical protein	291	1455
lpg2260	PHA synthase	1851	1455
lpg2261	phosphate acetyl/butyryltransferase family protein) includes: (de)hydratase mit MaoC domain)	1407	1455
lpg2262	acetate kinase	1119	1455
lpg2263	curved DNA binding protein DnaJ	891	1455
lpg2266	hypothetical protein	552	1455
lpg2267	prolidase	1239	1455
lpg2271	hypothetical protein	651	1455
lpg2272	transmembrane protein	492	1455
lpg2274	glycerophosphoryl diester esterase	720	1455
lpg2275	hypothetical protein	708	1455
lpg2276	Glu/Leu/Phe/Val dehydrogenase	1074	1455

lpg2277	O-methyltransferase, SAM-dependent	657	1455
lpg2278	4-hydroxyphenylpyruvate dioxygenase	1086	1455
lpg2279	fumarylacetoacetate hydrolase	999	1455
lpg2280	glutathione S-transferase	639	1455
lpg2281	hypothetical protein	561	1455
lpg2282	asparaginyl tRNA synthetase	1437	1455
lpg2285	lipoprotein ABC transporter	1356	1455
lpg2295	ribosomal large subunit (23S rRNA) pseudouridine synthase C	954	1455
lpg2297	ribonuclease E	2004	1455
lpg2298	inclusion membrane protein A	1278	1455
lpg2302	aspartate semialdehyde dehydrogenase	1023	1455
lpg2306	rhodanese domain protein	420	1455
lpg2307	glutaredoxin 3	255	1455
lpg2310	glutamate racemase	867	1455
lpg2312	hypothetical protein	303	1455
lpg2314	dihydropicolinate synthase	873	1455
lpg2315	hypothetical protein	285	1455
lpg2318	chemotaxis (motility protein A) transmembrane	906	1455
lpg2319	chemotaxis (motility protein B) transmembrane	939	1455
lpg2321	serine transporter	1362	1455
lpg2322	cardiac ankyrin repeat protein	1926	1455
lpg2327	CG18304 gene product	894	1455
lpg2328	hypothetical protein	384	1455
lpg2334	hypothetical protein	279	1455
lpg2335	glutamyl tRNA reductase	1383	1455
lpg2338	DnaK suppressor protein	477	1455
lpg2340	3-deoxy-D-manno-oct-2-ulosonic acid transferase	1266	1455
lpg2343	lysophospholipase A	978	1455
lpg2347	2,4-dienoyl-CoA reductase FadH1	2025	1455
lpg2348	superoxide dismutase (copper-zinc)	489	1455
lpg2352	malate dehydrogenase	993	1455
lpg2353	NUDIX hydrolase	564	1455
lpg2354	(oxygen-independent) coproporphyrinogen III oxidase	1128	1455
lpg2386	hypothetical protein	564	1455
lpg2388	amino acid permease	1731	1455
lpg2389	catalase-peroxidase KatB	2196	1455
lpg2391	SdbC	1305	1455
lpg2396	transcriptional regulator	1071	1455
lpg2404	hypothetical protein	915	1455
lpg2405	mutator MutT protein	402	1455
lpg2411	hypothetical protein	828	1455
lpg2413	hypothetical protein	438	1455
lpg2414	hypothetical protein	294	1455
lpg2434	hypothetical protein	495	1455
lpg2435	hypothetical protein	1044	1455
lpg2436	hypothetical protein	381	1455

lpg2438	florfenicol efflux pump	1191	1455
lpg2440	glutathione S-transferase	1017	1455
lpg2442	PhnB protein	411	1455
lpg2443	hypothetical protein	558	1455
lpg2445	hypothetical protein	486	1455
lpg2453	hypothetical protein	450	1455
lpg2459	guanylate cyclase	561	1455
lpg2460	hypothetical protein	384	1455
lpg2461	hypothetical protein	639	1455
lpg2463	peptide aspartate b-dioxygenase	720	1455
lpg2472	hydrogenase expression/formation protein HypD	1107	1455
lpg2473	hydrogenase expression/formation protein HypC	228	1455
lpg2475	hydrogenase expression/formation protein HypB	759	1455
lpg2476	hydrogenase nickel incorporation protein HypA	342	1455
lpg2483	hypothetical protein	558	1455
lpg2484	ribosomal protein Ham1	585	1455
lpg2485	TPR domain protein	1716	1455
lpg2487	deoxyuridinetriphosphatase	471	1455
lpg2491	hypothetical protein	564	1455
lpg2495	homospermidine synthase	1419	1455
lpg2497	hypothetical protein	654	1455
lpg2500	carbonic anhydrase Mig5	738	1455
lpg2513	RND multidrug efflux membrane fusion protein	1167	1455
lpg2514	outer membrane efflux protein (RND multidrug efflux)	1563	1455
lpg2516	major facilitator family transporter	1266	1455
lpg2517	transcriptional regulator, AsnC family	474	1455
lpg2520	hypothetical protein	369	1455
lpg2531	chorismate mutase/prephenate dehydratase (P-protein)	585	1455
lpg2532	aspartate aminotransferase	1167	1455
lpg2534	hypothetical protein	432	1455
lpg2535	myoglobin-like	408	1455
lpg2538	hypothetical protein	1416	1455
lpg2544	membrane-bound lytic murein transglycosylase A	1374	1455
lpg2549	transcriptional regulator, AraC-family	771	1455
lpg2554	rare lipoprotein A	483	1455
lpg2576	hypothetical, uroporphyrin-III C-methyltransferase	378	1455
lpg2578	hypothetical protein	255	1455
lpg2579	hypothetical protein	414	1455
lpg2580	glutaryl CoA dehydrogenase	1158	1455
lpg2585	D-alanyl-D-alanine dipeptidase	732	1455
lpg2587	probable thermolabile hemolysin	1551	1455
lpg2589	D-alanyl-D-alanine carboxypeptidase, fraction B; penicillin binding protein 4	1794	1455
lpg2592	hypothetical protein	750	1455
lpg2596	signal peptide protein, LysM domain protein	1038	1455
lpg2598	hypothetical protein	417	1455

lpg2601	hypothetical protein	441	1455
lpg2602	conserved domain protein	423	1455
lpg2604	hypothetical protein	804	1455
lpg2606	glutamine amidotransferase	867	1455
lpg2611	cell division protein FtsQ	720	1455
lpg2614	UDP-N-acetylmuramate:L-alanine ligase MurC	1410	1455
lpg2615	cell division protein FtsW	1185	1455
lpg2619	cell division protein ZipA	780	1455
lpg2621	acid phosphatase, class B	681	1455
lpg2622	hypothetical protein	1062	1455
lpg2624	transcription elongation factor GreA	483	1455
lpg2626	hypothetical protein	273	1455
lpg2628	membrane protein	753	1455
lpg2629	permease	1071	1455
lpg2632	DNA polymerase III, chi subunit	435	1455
lpg2636	30S ribosomal protein S20	267	1455
lpg2641	enhanced entry protein EnhA	723	1455
lpg2645	excinuclease ABC subunit	1857	1455
lpg2651	50S ribosomal protein L21	312	1455
lpg2652	50S ribosomal protein L25, ribosomal 5S rRNA E-loop binding protein	660	1455
lpg2653	peptidyl tRNA hydrolase	570	1455
lpg2656	octaprenyl diphosphate synthase IspB	969	1455
lpg2658	ferrous iron transporter A	228	1455
lpg2659	ATPase N2B (nucleotide (GTP) binding protein)	1092	1455
lpg2661	3-methyl-2-oxobutanoate hydroxymethyltransferase	867	1455
lpg2663	hypothetical protein	534	1455
lpg2666	probable hydrolase	882	1455
lpg2667	RNA polymerase sigma-32 factor RpoH	879	1455
lpg2668	cell division ATP transporter FtsX	930	1455
lpg2672	zinc protease (peptidase, M16 family)	1305	1455
lpg2673	N6-adenine specific methylase	546	1455
lpg2677	5'-nucleotidase	1728	1455
lpg2678	hypothetical protein	798	1455
lpg2682	hypothetical with two candidate membrane-spanning segments	708	1455
lpg2684	hypothetical protein	861	1455
lpg2687	IcmV	456	1455
lpg2688	IcmW	456	1455
lpg2690	LphB	1632	1455
lpg2692	hypothetical protein	531	1455
lpg2693	hypothetical SnoK-like protein	801	1455
lpg2694	phytanoyl-CoA dioxygenase	858	1455
lpg2696	tRNA delta(2)-isopentenylpyrophosphate transferase	966	1455
lpg2698	N-acetylmuramoyl-L-alanine amidase	1431	1455
lpg2701	stringent starvation protein B	396	1455
lpg2704	ubiquinol-cytochrome c reductase, cytochrome b	1215	1455

lpg2705	ubiquinol-cytochrome c reductase, iron-sulfur subunit	627	1455
lpg2706	30S ribosomal protein S9	432	1455
lpg2707	50S ribosomal protein L13	459	1455
lpg2709	integration host factor (IHF) alpha subunit	300	1455
lpg2712	50S ribosomal protein L20	360	1455
lpg2714	threonyl tRNA synthase	1941	1455
lpg2716	hypothetical protein	288	1455
lpg2717	hypothetical protein	486	1455
lpg2719	hypothetical protein	1152	1455
lpg2720	cNMP binding domain-containing protein	1032	1455
lpg2722	NADH-dependent flavin oxidoreductase, Oye family	1077	1455
lpg2724	hypothetical protein	345	1455
lpg2726	peptidylprolyl cis-trans isomerase B (cyclophilin-type) Lcy	495	1455
lpg2727	queuine/archaeosine tRNA-ribosyltransferase	1167	1455
lpg2732	(two component) response regulator	1026	1455
lpg2735	porphobilinogen deaminase	966	1455
lpg2737	uroporphyrinogen III methylase	1125	1455
lpg2739	cation efflux system protein	924	1455
lpg2740	hypothetical protein	663	1455
lpg2741	oligoribonuclease	564	1455
lpg2742	tRNA nucleotidyltransferase	1275	1455
lpg2743	EngC GTPase	978	1455
lpg2755	hypothetical protein	339	1455
lpg2756	recombinational DNA repair protein RecR	600	1455
lpg2758	hypothetical protein	1998	1455
lpg2763	Mg2+ and Co2+ transporter CorB, hemolysin	1266	1455
lpg2766	GTP cyclohydrolase I	567	1455
lpg2769	30S ribosomal protein S15 (S15/S13E)	276	1455
lpg2773	N utilization substance protein A	1479	1455
lpg2774	hypothetical protein	444	1455
lpg2777	NADH dehydrogenase I, M subunit	1506	1455
lpg2779	NADH dehydrogenase I, K subunit	306	1455
lpg2780	NADH dehydrogenase I, J subunit	660	1455
lpg2781	NADH dehydrogenase I, I subunit	501	1455
lpg2783	NADH dehydrogenase I, G subunit	2352	1455
lpg2786	NADH dehydrogenase I, D subunit	1269	1455
lpg2787	NADH dehydrogenase I, C subunit	684	1455
lpg2789	NADH dehydrogenase I, A subunit	357	1455
lpg2791	preprotein translocase, SecG subunit	306	1455
lpg2792	triosephosphate isomerase (TIM)	750	1455
lpg2795	7,8-dihydropteroate synthase	876	1455
lpg2796	cell division protein FtsH	1920	1455
lpg2797	ribosomal RNA large subunit methyltransferase J	744	1455
lpg2798	RNA-binding protein containing KH domina, putative pseudogene	251	1455
lpg2799	O-acetyltransferase	1977	1455

lpg2809	aminopeptidase N	2598	1455
lpg2812	sporulation protein	1524	1455
lpg2817	heat shock protein 33, redox regulated chaperonin	864	1455
lpg2818	hypothetical protein	498	1455
lpg2822	virulence regulator BipA	1827	1455
lpg2823	sugar kinase	888	1455
lpg2825	cold shock protein CspE	207	1455
lpg2827	hypothetical protein	978	1455
lpg2833	acyl-CoA thioester hydrolase	381	1455
lpg2835	thiopurine S-methyltransferase	666	1455
lpg2836	glucosamine-fructose-6-phosphate aminotransferase, isomerizing	1815	1455
lpg2837	phospholipase/lecithinase/hemolysin, lysophospholipase A, glycerophospholipid-cholesterol acyltransferase	1302	1455
lpg2838	rhodanese domain protein	765	1455
lpg2842	PhoH protein (phosphate starvation inducible protein)	1407	1455
lpg2847	hypothetical protein	963	1455
lpg2851	protoporphyrinogen oxidase	1509	1455
lpg2853	hypothetical protein, KQDN repeats	1659	1455
lpg2855	TPR (repeat) domain protein	933	1455
lpg2858	hypothetical protein	912	1455
lpg2868	thymidylate synthase (TS)	795	1455
lpg2872	(di)nucleoside polyphosphate hydrolase	528	1455
lpg2873	L-asparaginase I (cytoplasmic)	1011	1455
lpg2875	UDP-N-acetylglucosamine pyrophosphorylase	1386	1455
lpg2879	hypothetical protein	1752	1455
lpg2881	iron-sulfur cluster binding protein	615	1455
lpg2883	3-octaprenyl-4-hydroxybenzoate carboxy-lyase	570	1455
lpg2884	hypothetical protein	738	1455
lpg2885	hypothetical protein	555	1455
lpg2886	ExsB protein	702	1455
lpg2887	phosphomannose isomerase GDP mannose pyrophosphorylase	1494	1455
lpg2891	sporulation initiation inhibitor protein Soj	771	1455
lpg2894	cytochrome c oxidase, subunit III	870	1455
lpg2897	cytochrome c oxidase, subunit II	1206	1455
lpg2899	ferredoxin component, putative pseudogene	350	1455
lpg2900	CapM protein, capsular polysaccharide biosynthesis	1029	1455
lpg2901	transporter, LysE family	606	1455
lpg2905	ubiquinone biosynthesis AarF	1650	1455
lpg2907	hypothetical protein	1263	1455
lpg2916	hypothetical protein	537	1455
lpg2924	lipoprotein	1146	1455
lpg2926	bis(5'-nucleosyl)tetraphosphatase, symmetrical	846	1455
lpg2928	dimethyladenosine transferase	771	1455
lpg2929	aspartate-1-decarboxylase	402	1455
lpg2931	hypothetical protein	324	1455

lpg2937	fumarate hydratase	1395	1455
lpg2951	cystathionine beta synthase	951	1455
lpg2953	hypothetical protein	726	1455
lpg2956	deoxycytidine triphosphate deaminase	567	1455
lpg2960	major outer membrane protein	972	1455
lpg2962	sodium-type flagellar protein	900	1455
lpg2963	dihydroorotase, homodimeric type	1080	1455
lpg2964	ribonuclease T	624	1455
lpg2966	glutaredoxin-related protein	270	1455
lpg2968	N-acetylornithine aminotransferase ArgD	1170	1455
lpg2970	glycerophosphoryl diester phosphodiesterase	789	1455
lpg2971	malate dehydrogenase (NAD-linked), malic enzyme	1671	1455
lpg2972	SUA5/yciO/yrdC family:Sua5/YciO/YrdC/YwlC protein family	969	1455
lpg2974	phosphatidylserine decarboxylase	852	1455
lpg2976	hypothetical protein	1530	1455
lpg2982	H+-transporting two-sector ATPase, ATP synthase F1 subunit beta	1377	1455
lpg2985	ATP synthase F1, delta subunit	558	1455
lpg2986	ATP synthase F0, B subunit	471	1455
lpg2990	hypothetical protein	144	1455
lpg2991	hemolysin, lipoprotein	588	1455
lpg2993	phosphoheptose isomerase	600	1455
lpg2995	lipoprotein	1812	1455
lpg2996	tetrapyrrole (corrin/porphyrin) methylase	852	1455
lpg2998	sulfate transporter	2178	1455
lpg3002	inner membrane protein, 60 kDa	1671	1455

Table S9. 200 “accessory” genes used in the gene presence/absence scheme.
The reference gene sequences are deposited in the ENA under the accession numbers FJOD01000001-FJOD01000200.

**Gene no.**	**Annotation**	**Length (bp)**	**Reference isolate**
1	hypothetical protein	2895	EUL 24
2	hypothetical protein	339	EUL 24
3	hypothetical protein	1410	EUL 24
4	Fatty acid hydroxylase superfamily	873	EUL 24
5	hypothetical protein	621	EUL 24
6	hypothetical protein	1440	EUL 24
7	hypothetical protein	231	EUL 24
8	hypothetical protein	1413	EUL 24
9	Serine/threonine-protein kinase HipA	306	EUL 24
10	Heme NO binding	540	EUL 24
11	Transcriptional repressor smtB homolog	294	EUL 24

12	transcriptional repressor DicA	252	EUL 24
13	Carbon storage regulator	198	EUL 24
14	integrating conjugative element protein PilL	411	EUL 24
15	hypothetical protein	585	EUL 24
16	Phage integrase	804	EUL 24
17	Phage integrase	897	EUL 24
18	hypothetical protein	1260	EUL 24
19	hypothetical protein	666	EUL 24
20	hypothetical protein	1065	EUL 24
21	Hsp20/alpha crystallin family	567	EUL 24
22	Opacity protein and related surface antigens	783	EUL 24
23	hypothetical protein	621	EUL 24
24	conjugal transfer protein TrbB	492	EUL 24
25	conjugal pilus assembly protein TraF	780	EUL 24
26	conjugal transfer mating pair stabilization protein TraN	1809	EUL 24
27	conjugal transfer pilus assembly protein TrbC	663	EUL 24
28	hypothetical protein	2547	EUL 24
29	conjugal transfer pilus assembly protein TraB	1464	EUL 24
30	conjugal transfer protein TraK	732	EUL 24
31	Predicted acetyltransferase	1248	EUL 24
32	aminoalkylphosphonic acid N-acetyltransferase	450	EUL 24
33	Predicted acetyltransferase	1035	EUL 24
34	Domain of unknown function (DUF932)	801	EUL 24
35	hypothetical protein	384	EUL 24
36	Predicted acetyltransferase	1008	EUL 24
37	hypothetical protein	1380	EUL 36
38	phosphonate utilization associated putative membrane protein	831	EUL 36
39	Ribonuclease TTHA0252	1359	EUL 36
40	hypothetical protein	450	EUL 36
41	poly(R)-hydroxyalkanoic acid synthase	1677	EUL 36
42	Predicted membrane protein	525	EUL 36
43	Protein of unknown function (DUF2933)	294	EUL 36
44	Putative protein-S-isoprenylcysteine methyltransferase	663	EUL 36
45	hypothetical protein	819	EUL 36
46	hypothetical protein	759	EUL 36
47	3-ketosteroid-9-alpha-hydroxylase reductase subunit	1887	EUL 36
48	ATP synthase subunit alpha	1470	EUL 36
49	F-type ATPase subunit b	741	EUL 36
50	Lipid-binding protein	273	EUL 36
51	F-ATPase subunit 6	690	EUL 36
52	putative F0F1-ATPase subunit	276	EUL 36
53	F0F1 ATP synthase subunit epsilon	408	EUL 36
54	hypothetical protein	429	EUL 36
55	hypothetical protein	255	EUL 36
56	Predicted transcriptional regulator	201	EUL 36
57	Putative prophage CPS-53 integrase	1179	EUL 36
58	hypothetical protein	270	EUL 36

59	hypothetical protein	807	EUL 36
60	hypothetical protein	207	EUL 36
61	Pathogenicity locus	294	EUL 36
62	Thiocyanate hydrolase subunit beta	423	EUL 36
63	Thiocyanate hydrolase subunit gamma	666	EUL 36
64	HupE / UreJ protein	624	EUL 36
65	Opacity protein and related surface antigens	708	EUL 36
66	SNARE domain	291	EUL 36
67	Universal stress protein E homolog	936	EUL 36
68	Ankyrin repeats (3 copies)	1932	EUL 36
69	HTH-type transcriptional regulator gltR	873	EUL 36
70	Aspartate aminotransferase	1356	EUL 36
71	Proline porter II	1275	EUL 36
72	Hypoxic response protein 1	447	EUL 36
73	hypothetical protein	1434	EUL 36
74	hypothetical protein	870	EUL 36
75	type IV secretion system protein VirB3	279	EUL 36
76	Type IV secretion system protein virB4	2478	EUL 36
77	P-type DNA transfer protein VirB5	708	EUL 36
78	TrbL/VirB6 plasmid conjugal transfer protein	1038	EUL 36
79	Type IV secretion system protein virB8	714	EUL 36
80	Type IV secretion system protein virB9 precursor	750	EUL 36
81	Type IV secretion system protein virB10	1089	EUL 36
82	Conjugal transfer protein traG	1899	EUL 36
83	hypothetical protein	204	EUL 36
84	hypothetical protein	456	EUL 36
85	hypothetical protein	351	EUL 36
86	Bacterial regulatory proteins	699	EUL 36
87	Dot/Icm substrate protein	4605	EUL 36
88	hypothetical protein	318	EUL 36
89	hypothetical protein	1917	EUL 36
90	hypothetical protein	2502	EUL 36
91	Transposase	1194	EUL 36
92	Legionella pneumophila major outer membrane protein precursor	975	EUL 36
93	phenylacetate-CoA ligase	1380	EUL 36
94	hypothetical protein	522	EUL 36
95	hypothetical protein	1137	EUL 36
96	Inner membrane protein ybaL	1692	EUL 36
97	hypothetical protein	249	EUL 36
98	hypothetical protein	1206	EUL 48
99	Rieske [2Fe-2S] domain	258	EUL 48
100	hypothetical protein	1392	EUL 48
101	Flavin reductase like domain	633	EUL 48
102	hypothetical protein	1344	EUL 48
103	precorrin 6A synthase	753	EUL 48
104	hypothetical protein	195	EUL 48
105	Major Facilitator Superfamily	1224	EUL 48

106	hypothetical protein	369	EUL 48
107	Dipeptide and tripeptide permease A	1479	EUL 48
108	ATP synthase subunit beta	1422	EUL 48
109	hypothetical protein	372	EUL 48
110	hypothetical protein	165	EUL 48
111	Ribose-phosphate pyrophosphokinase	930	EUL 48
112	Pyrimidine-nucleoside phosphorylase	1515	EUL 48
113	Serine/threonine-protein kinase HipA	1302	EUL 48
114	serine/threonine protein kinase	978	EUL 48
115	hypothetical protein	948	EUL 48
116	hypothetical protein	150	EUL 48
117	Uncharacterized protein conserved in bacteria	2745	EUL 48
118	Superfamily II helicase and inactivated derivatives	1773	EUL 48
119	Regulator of chromosome condensation (RCC1) repeat	1440	EUL 48
120	hypothetical protein	816	EUL 48
121	hypothetical protein	558	EUL 48
122	hypothetical protein	900	EUL 48
123	Transposase and inactivated derivatives	1173	EUL 54
124	Antitoxin HipB	273	EUL 54
125	Phage integrase	801	EUL 54
126	hypothetical protein	381	EUL 54
127	hypothetical protein	2229	EUL 54
128	Integrase core domain	1032	EUL 54
129	GIY-YIG nuclease superfamily protein	291	EUL 55
130	Dot/Icm substrate protein	4599	EUL 55
131	Ribulose-5-phosphate 4-epimerase and related epimerases and aldolases	1752	EUL 55
132	Ribulose-5-phosphate 4-epimerase and related epimerases and aldolases	1740	EUL 55
133	GIY-YIG nuclease superfamily protein	354	EUL 55
134	hypothetical protein	750	EUL 55
135	anaerobic benzoate catabolism transcriptional regulator	261	EUL 55
136	hypothetical protein	420	EUL 55
137	hypothetical protein	900	EUL 55
138	Phage integrase	900	EUL 55
139	hypothetical protein	171	EUL 55
140	Relaxosome protein	351	EUL 123
141	Conjugal transfer protein traG	1887	EUL 123
142	conjugal transfer protein TrbJ	738	EUL 123
143	conjugal transfer protein TrbG	873	EUL 123
144	conjugal transfer protein TrbC	375	EUL 123
145	Pertussis toxin liberation protein H	963	EUL 123
146	Carbon storage regulator	258	EUL 123
147	hypothetical protein	882	EUL 123
148	Pyrimidine-nucleoside phosphorylase	1512	EUL 123
149	Phosphate acetyltransferase	1404	EUL 123
150	Enoyl-[acyl-carrier-protein] reductase [NADH] FabI	753	EUL 123
151	Spermidine N(1)-acetyltransferase	555	EUL 123

152	Tetrapyrrole (Corrin/Porphyrin) Methylases	774	EUL 123
153	hypothetical protein	183	EUL 123
154	Aminoglycoside phosphotransferase	879	EUL 123
155	hypothetical protein	1923	EUL 63
156	Sodium/proton antiporter nhaA	1152	EUL 63
157	Regulator of chromosome condensation (RCC1) repeat	1440	EUL 63
158	Integrase	897	EUL 63
159	Ankyrin repeats (3 copies)	1524	EUL 63
160	hypothetical protein	1083	EUL 63
161	DNA primase TraC	2196	EUL 63
162	Probable cadmium-transporting ATPase	1905	EUL 69
163	hypothetical protein	1365	EUL 71
164	hypothetical protein	1050	H123640643
165	Calcium-transporting ATPase	2706	EUL 88
166	hypothetical protein	366	LC6408
167	hypothetical protein	903	EUL 159
168	hypothetical protein	240	EUL 167
169	hypothetical protein	342	EUL 167
170	conjugal transfer mating pair stabilization protein TraG	2889	H073900557
171	Transposase and inactivated derivatives	1209	H081180019
172	Murein tetrapeptide carboxypeptidase	939	H113660550
173	Thiocyanate hydrolase subunit alpha	309	H064380001
174	hypothetical protein	1134	H064180019
175	F0F1 ATP synthase subunit gamma	945	H073340594
176	hypothetical protein	468	H073340594
177	Transposase and inactivated derivatives	747	EUL 18
178	Cyn operon transcriptional activator	942	EUL 25
179	Cation efflux system protein CzcC	1329	EUL 25
180	Type IV secretion system protein virB11	1056	EUL 25
181	hypothetical protein	990	EUL 25
182	conjugal transfer protein TrbF	741	EUL 140
183	Uncharacterized conserved protein (contains double-stranded beta-helix domain)	858	EUL 140
184	hypothetical protein	393	EUL 140
185	Site-specific DNA methylase	1413	EUL 126
186	Aminoglycoside phosphotransferase	1614	EUL 4
187	hypothetical protein	1299	EUL 103
188	Putative prophage CPS-53 integrase	1242	EUL 111
189	acetyl-CoA acetyltransferase	1059	EUL 144
190	hypothetical protein	1950	EUL 149
191	Domain of unknown function (DUF1768)	1842	EUL 154
192	conjugal transfer protein TrbL	1440	EUL 162
193	Bacteriophytochrome cph2	2634	EUL 162
194	Superfamily II helicase and inactivated derivatives	1752	EUL 163
195	Guanine deaminase	480	HL06041035
196	hypothetical protein	450	H093380153
197	Antirestriction protein	507	H044500045

198	Ran GTPase-activating protein (RanGAP) involved in mRNA processing and transport	993	H091960009
199	Transcriptional regulatory protein RstA	726	H091960009
200	hypothetical protein	516	H071260094

Table S10. A summary of sequencing statistics for the typing panel isolates and all isolates used in this study (excluding the two complete genomes).

**Quality criteria**	**Mean (and range)**	
	Typing panel only (n=106)	*All isolates except complete genomes (n=333)*
Number of reads	4,445,116 (2,659,918 – 5,908,566)	4,427,999 (1,750,804 – 20,104,220)
Mapping depth	124.6x (71.4x - 164.2x)	122.8x (49.4x– 211.2x)
% of the reference length mapped	96.9 (92.3-100)	97.3 (92.3-100)
Assembly length (bp)	3,471,546 (3,229,839 – 3,682,698)	3,476,413 (3,229,839 – 3,710,927)
Number of contigs	35.1 (15 - 72)	39.9 (12 - 140)
N50 (bp)	250,252 (86,373 - 726,453)	249,102 (81,272 – 2,134,649)

Table S11. The number of typable loci in each isolate for each extended MLST scheme.
The number of loci identified as typable by BIGSdb (i.e. only excluding absent or truncated loci) and after the additional QC steps (excluding any loci containing "Ns", any loci with <20 nucleotides, or any loci not validated by mapping data) are given.

**EUL/isolate number**	**Number of alleles called pre- and post-QC**											
	**rMLST (53)**		**cgMLST (50)**		**cgMLST (100)**		**cgMLST (500)**		**cgMLST (1455)**		**cgMLST (1521)**	
1	53	53	50	50	100	100	500	500	1455	1455	1521	1521
2	53	53	50	50	100	100	500	500	1455	1455	1521	1521
3	53	53	50	50	100	100	500	500	1455	1455	1521	1521
4	53	53	50	50	100	100	500	500	1455	1455	1521	1518
6	53	52	50	50	100	100	500	500	1455	1455	1521	1519
7	53	53	50	50	100	100	500	500	1455	1455	1521	1519
8	53	53	50	50	100	100	500	500	1455	1455	1521	1520
13	53	53	50	50	100	100	500	500	1455	1455	1521	1520
14	53	52	50	50	100	100	500	500	1455	1455	1521	1520
16	53	53	50	50	100	100	500	500	1455	1455	1521	1521
17	53	53	50	50	100	100	500	500	1455	1455	1521	1521
18	53	52	50	50	100	100	500	500	1455	1454	1519	1517
19	53	53	50	50	100	100	500	500	1455	1455	1521	1520
20	53	53	50	50	100	100	500	500	1455	1455	1521	1520
25	53	53	50	50	100	100	500	500	1455	1455	1521	1520
26	53	53	50	50	100	100	500	500	1455	1454	1521	1520
27	53	53	50	50	100	100	500	500	1455	1455	1521	1517
27 (replicate)	53	53	50	50	100	100	500	500	1455	1455	1521	1519

28	53	53	50	50	100	100	500	500	1455	1455	1521	1519
29	53	53	50	50	100	100	500	500	1455	1455	1521	1520
30	53	53	50	50	100	100	500	500	1455	1455	1521	1521
31	53	53	50	50	100	100	500	500	1455	1455	1521	1519
32	53	53	50	50	100	100	500	500	1455	1455	1521	1521
33	53	53	50	50	100	100	500	500	1455	1455	1521	1520
33 (replicate)	53	53	50	50	100	100	500	500	1455	1455	1521	1521
36	53	53	50	50	100	100	500	500	1455	1455	1521	1520
37	53	53	50	50	100	100	500	500	1455	1455	1521	1521
38	53	53	50	50	100	100	500	500	1455	1455	1521	1520
39	53	53	50	50	100	100	500	500	1455	1455	1521	1521
40	53	53	50	50	100	100	500	500	1455	1455	1521	1520
41	53	53	50	50	100	100	500	500	1455	1455	1521	1520
42	53	53	50	50	100	100	500	500	1455	1455	1521	1520
43	53	53	50	50	100	100	500	500	1455	1455	1521	1520
48	53	52	50	50	100	100	500	500	1455	1455	1521	1519
49	53	53	50	50	100	100	500	500	1455	1455	1521	1521
50	53	53	50	48	100	98	500	497	1455	1451	1521	1514
51	53	53	50	50	100	100	500	500	1455	1454	1521	1519
52	53	53	50	50	100	100	500	500	1455	1455	1521	1521
53	53	53	50	50	100	100	500	500	1455	1455	1521	1520
54	53	53	50	50	100	100	500	499	1455	1454	1521	1519
55	53	53	50	50	100	100	500	500	1455	1455	1521	1521
60	53	53	50	50	100	100	500	500	1455	1455	1521	1520
63	53	53	50	50	100	100	500	500	1455	1455	1521	1520
66	53	53	50	50	100	100	500	500	1455	1455	1521	1520
67	53	53	50	50	100	100	500	500	1455	1455	1521	1521
68	53	52	50	50	100	100	500	500	1455	1455	1521	1520
69	53	53	50	50	100	100	500	500	1455	1455	1521	1521
69 (replicate)	53	53	50	50	100	100	500	500	1455	1455	1521	1520
70	53	53	50	50	100	100	500	500	1455	1455	1521	1521
71	53	53	50	50	100	100	500	500	1455	1455	1521	1518
72	53	53	50	50	100	100	500	500	1455	1455	1521	1521
73	53	53	50	50	100	100	500	500	1455	1455	1521	1521
74	53	53	50	50	100	100	500	500	1455	1454	1521	1521
75	53	53	50	50	100	100	500	500	1455	1455	1521	1520
75 (replicate)	53	52	50	50	100	100	500	500	1455	1455	1520	1519
81	53	53	50	50	100	100	500	500	1455	1455	1521	1521
82	53	53	50	50	100	100	500	500	1455	1454	1521	1520
83	53	53	50	50	100	100	500	500	1455	1455	1521	1520
84	53	53	50	50	100	100	500	500	1455	1455	1521	1519
85	53	53	50	50	100	100	500	500	1455	1455	1521	1520
86	53	53	50	50	100	100	500	500	1455	1455	1521	1520
87	53	53	50	50	100	100	500	500	1455	1455	1521	1521
88	53	53	50	50	100	100	500	500	1455	1455	1521	1520
91	53	53	50	50	100	100	500	500	1455	1454	1521	1520
92	53	53	50	50	100	100	500	500	1455	1455	1521	1520
92 (replicate)	53	53	50	50	100	100	500	500	1455	1455	1521	1521
93	53	53	50	50	100	100	500	500	1455	1455	1521	1518
97	53	52	50	50	100	100	500	500	1455	1455	1521	1521

98	53	53	50	50	100	100	500	500	1455	1455	1521	1521
99	53	53	50	50	100	100	500	500	1455	1455	1521	1521
100	53	53	50	50	100	100	500	500	1455	1455	1521	1520
101	53	53	50	50	100	100	500	500	1455	1455	1521	1519
102	53	53	50	50	100	100	500	500	1455	1455	1521	1519
103	53	53	50	50	100	100	500	500	1455	1455	1521	1518
104	53	53	50	50	100	100	500	500	1455	1454	1521	1519
105	53	53	50	50	100	100	500	500	1455	1455	1521	1521
110	53	53	50	50	100	100	500	500	1455	1455	1521	1521
111	53	53	50	50	100	100	500	500	1455	1454	1519	1512
111 (replicate)	53	52	50	50	100	100	500	500	1455	1455	1518	1519
114	53	53	50	50	100	100	500	500	1455	1454	1521	1521
116	53	52	50	50	100	100	500	500	1455	1455	1521	1521
117	53	53	50	50	100	100	500	500	1455	1455	1521	1520
118	53	53	50	50	100	100	500	500	1455	1455	1521	1521
119	53	53	50	50	100	100	500	500	1455	1455	1521	1521
120	53	53	50	50	100	100	500	500	1455	1455	1521	1521
9	53	53	50	50	100	100	500	500	1455	1455	1521	1519
10	53	53	50	50	100	100	500	500	1455	1455	1521	1521
11	53	53	50	50	100	100	500	500	1455	1455	1521	1521
12	53	53	50	50	100	100	500	500	1455	1455	1521	1520
22	53	53	50	50	100	100	500	500	1455	1455	1521	1521
23	53	53	50	50	100	100	500	500	1455	1455	1521	1521
24	53	53	50	50	100	100	500	500	1455	1455	1521	1517
34	53	53	50	50	100	100	500	500	1455	1455	1521	1521
35	53	53	50	50	100	100	500	499	1455	1454	1521	1518
44	53	53	50	50	100	100	500	500	1455	1455	1521	1520
45	53	53	50	50	100	100	500	500	1455	1455	1521	1521
46	53	53	50	50	100	100	500	500	1455	1455	1521	1521
47	53	53	50	50	100	100	500	500	1455	1455	1521	1521
56	53	53	50	50	100	100	500	500	1455	1455	1521	1521
57	53	53	50	50	100	100	500	500	1455	1455	1521	1519
58	53	53	50	50	100	100	500	500	1455	1455	1521	1521
59	53	53	50	50	100	100	500	500	1455	1454	1521	1518
76	53	53	50	50	100	100	500	500	1455	1455	1521	1518
77	53	53	50	50	100	100	500	500	1455	1455	1521	1520
78	53	53	50	50	100	100	500	500	1455	1455	1521	1520
79	53	53	50	50	100	100	500	500	1455	1455	1521	1521
94	53	53	50	50	100	100	500	500	1455	1454	1521	1520
95	53	53	50	50	100	100	500	500	1455	1455	1521	1519
96	53	53	50	50	100	100	500	500	1455	1455	1521	1519
106	53	52	50	50	100	100	500	500	1455	1455	1521	1519
107	53	52	50	50	100	100	500	500	1455	1455	1521	1520
121	53	52	50	50	100	100	500	500	1455	1455	1521	1521
LC 202/EUL 153	53	53	50	50	100	100	500	500	1455	1455	1521	1519
LC 206/EUL 158	53	53	50	50	100	100	500	500	1455	1455	1521	1520
LC 569/EUL 154	53	53	50	50	100	100	500	500	1455	1455	1521	1521
LC 606/EUL 155	53	53	50	50	100	100	500	500	1455	1455	1521	1521
LC 384/EUL 156	53	53	50	50	100	100	500	500	1455	1455	1516	1514
LC 395/EUL 159	53	53	50	50	100	100	500	500	1455	1455	1516	1514

LC6379-1/EUL 145	53	53	50	50	100	100	500	500	1455	1455	1521	1521
LC6376	53	53	50	50	100	100	500	500	1455	1455	1521	1520
LC6382	53	53	50	50	100	100	500	500	1455	1455	1521	1521
LC6391	53	53	50	50	100	100	500	500	1455	1455	1521	1520
LC6394	53	53	50	50	100	100	500	500	1455	1455	1521	1520
LC6397	53	53	50	50	100	100	500	500	1455	1455	1521	1519
LC6406	53	53	50	50	100	100	500	500	1455	1455	1521	1519
LC6407	53	53	50	50	100	100	500	500	1455	1455	1521	1520
LC6408	53	53	50	50	100	100	500	500	1455	1455	1521	1520
LC6411	53	53	50	50	100	100	500	500	1455	1455	1521	1521
LC6412	53	52	50	50	100	100	500	500	1455	1455	1521	1518
LC6413	53	53	50	50	100	100	500	500	1455	1455	1521	1520
LC6416	53	53	50	50	100	100	500	500	1455	1455	1521	1521
LC6418	53	53	50	50	100	100	500	500	1455	1455	1521	1519
LC6385	53	53	50	50	100	100	500	500	1455	1455	1521	1521
LC6388	53	53	50	50	100	100	500	500	1455	1455	1521	1520
LC6409	53	53	50	50	100	100	500	500	1455	1455	1521	1519
LC6410	53	53	50	50	100	100	500	500	1455	1455	1521	1521
LC0537/EUL 132	53	53	50	50	100	100	500	500	1455	1455	1521	1521
LC0539/EUL 133	53	52	50	50	100	100	500	500	1455	1454	1521	1519
LC0540/EUL 134	53	53	50	50	100	100	500	500	1455	1455	1521	1521
LC0565	53	53	50	50	100	100	500	500	1455	1455	1521	1521
LC0583	53	53	50	50	100	100	500	500	1455	1455	1521	1521
H034680033	53	52	50	46	100	95	500	480	1450	1400	1511	1455
H034680035/EUL 165	53	52	50	50	100	100	500	500	1455	1455	1521	1520
H034690056/EUL 166	53	52	50	50	100	100	500	500	1455	1455	1521	1519
H034800427	53	52	50	49	100	98	499	492	1454	1444	1519	1513
H034980467	53	53	50	50	100	100	500	500	1455	1454	1521	1520
Paris (complete genome)	53	NA	50	NA	100	NA	500	NA	1455	NA	1521	NA
H034800423	52	42	50	39	97	79	479	364	1394	1064	1456	1067
OLDA1 (NCTC12008)	53	53	50	50	100	100	500	500	1455	1454	1521	1519
EUL 109	53	53	50	50	100	100	500	500	1455	1455	1521	1521
H064240448	53	53	50	50	100	100	500	500	1455	1455	1521	1521
LC0731	53	53	50	50	100	100	500	500	1455	1455	1521	1521
LC0732	53	53	50	50	100	100	500	500	1455	1455	1521	1521
LC0763	53	53	50	50	100	100	500	500	1455	1455	1521	1521
LC0782	53	53	50	50	100	100	500	500	1455	1455	1521	1521
LC0795	53	52	50	50	100	100	500	500	1455	1455	1521	1521
LC0801	53	53	50	50	100	100	500	500	1455	1455	1521	1520
LC5694	53	53	50	50	100	100	500	500	1455	1455	1504	1504
LC5722	53	53	50	50	100	100	500	500	1455	1455	1521	1519
LC5738	53	53	50	50	100	100	500	500	1455	1455	1504	1504
LC5755	53	53	50	50	100	100	500	500	1455	1455	1521	1521
LC6163	53	53	50	50	100	100	500	500	1455	1455	1521	1520
LC6267	53	53	50	50	100	100	500	500	1455	1455	1521	1521
LC6268	53	53	50	50	100	100	500	500	1455	1455	1521	1519
LC6228	53	53	50	50	100	100	500	500	1455	1455	1521	1521
H041380048	53	53	50	50	100	100	500	500	1455	1455	1521	1521
H041640791	53	53	50	50	100	100	500	500	1455	1455	1521	1521
H042960010	53	53	50	50	100	100	500	500	1455	1455	1521	1521

H061140013	53	53	50	50	100	100	500	500	1455	1455	1521	1521
H071880001	53	53	50	50	100	100	500	500	1455	1455	1521	1520
H073060003	53	53	50	50	100	100	500	500	1455	1455	1521	1521
H080820009	53	53	50	50	100	100	500	500	1455	1454	1521	1520
LC6058	53	53	50	50	100	100	500	500	1455	1455	1521	1520
LC6293	53	52	50	50	100	100	500	500	1455	1455	1521	1519
LC6788	53	53	50	50	100	100	500	500	1455	1455	1521	1521
H062660463	53	53	50	50	100	100	500	500	1455	1455	1521	1521
H073900557	53	53	50	50	100	100	500	500	1455	1455	1521	1520
LC1127	53	53	50	50	100	100	500	500	1455	1454	1521	1520
H084760449	53	53	50	50	100	100	500	500	1455	1455	1521	1520
H085020185	53	53	50	50	100	100	500	500	1455	1455	1521	1521
H090320386	53	53	50	50	100	100	500	500	1455	1455	1521	1521
H044260061	53	53	50	50	100	100	500	500	1455	1455	1521	1521
H093140322	53	53	50	50	100	100	500	500	1455	1455	1521	1521
H093160422	53	53	50	50	100	100	500	500	1455	1455	1521	1521
H092760433	53	52	50	50	100	100	500	500	1455	1454	1521	1518
H100940111	53	53	50	50	100	100	500	500	1455	1455	1521	1521
H101760092	53	53	50	50	100	100	500	500	1455	1455	1521	1521
H101820190	53	53	50	50	100	100	500	500	1455	1455	1521	1521
H102020414	53	53	50	50	100	100	500	500	1455	1455	1521	1521
H101980130	53	53	50	50	100	100	500	500	1455	1455	1521	1521
H103820081	53	53	50	50	100	100	500	500	1455	1455	1521	1520
H120240685	53	53	50	50	100	100	500	500	1455	1455	1521	1520
H104320293	53	53	50	50	100	100	500	500	1455	1455	1521	1520
H113180118	53	53	50	50	100	100	500	500	1455	1455	1504	1504
H113340664	53	53	50	50	100	100	500	500	1455	1455	1504	1504
H113280076	53	53	50	50	100	100	500	500	1455	1455	1521	1521
H113660550	53	53	50	50	100	100	500	500	1455	1455	1521	1519
H114740454	53	53	50	50	100	100	500	500	1455	1455	1521	1520
H115040456	53	53	50	50	100	100	500	500	1455	1455	1521	1521
H111580389	53	52	50	50	100	100	500	500	1455	1455	1521	1520
H113780240	53	53	50	50	100	100	500	500	1455	1455	1521	1521
H083920177	53	53	50	50	100	100	500	500	1455	1455	1519	1517
H084140691	53	53	50	50	100	100	500	500	1455	1455	1521	1521
H081180019	53	53	50	50	100	100	500	500	1455	1455	1521	1521
H103260667	53	53	50	50	100	100	500	500	1455	1455	1521	1521
LC464	53	53	50	50	100	100	500	500	1455	1455	1521	1521
LC0512	53	53	50	50	100	100	500	500	1455	1455	1521	1521
LC0794	53	53	50	50	100	100	500	500	1455	1455	1521	1521
LC0798	53	53	50	50	100	100	500	500	1454	1454	1519	1519
LC0536/EUL 131	53	53	50	50	100	100	500	500	1455	1455	1521	1520
LC230/EUL 122	53	53	50	50	100	100	500	500	1455	1455	1521	1521
LC231/EUL 123	53	53	50	50	100	100	500	500	1455	1455	1521	1519
LC0462/EUL 124	53	53	50	50	100	100	500	500	1455	1455	1521	1518
LC0463/EUL 125	53	53	50	50	100	100	500	500	1455	1455	1521	1520
Lorraine (complete genome)	53	NA	50	NA	100	NA	500	NA	1455	NA	1521	NA
H063920004/EUL 169	53	53	50	50	100	100	500	500	1455	1455	1521	1519
H064160534/EULV0410	53	53	50	50	100	100	500	500	1455	1455	1521	1518
H064160538/EUL 170	53	53	50	50	100	100	500	500	1455	1455	1521	1518

H034700617	53	53	50	50	100	100	500	498	1455	1449	1521	1510
H043580159	53	52	50	50	100	100	500	500	1455	1455	1521	1519
H043580160	53	52	50	50	100	100	500	500	1455	1455	1521	1520
H043660021	53	53	50	50	100	100	500	500	1455	1455	1521	1519
H043680663	53	53	50	50	100	100	500	500	1455	1455	1521	1517
H043700021	53	52	50	50	100	100	500	500	1455	1455	1521	1518
H043790008	53	53	50	50	100	100	500	500	1455	1455	1521	1521
H052920051	53	53	50	50	100	100	500	500	1455	1455	1521	1518
H053540106	53	52	50	50	100	100	500	500	1455	1455	1521	1520
H063660005	53	53	50	50	100	100	500	500	1455	1455	1521	1520
H063660006	53	53	50	50	100	100	500	500	1455	1455	1521	1520
H063760006	53	53	50	50	100	100	500	500	1455	1455	1521	1521
H063660009	53	53	50	50	100	100	500	500	1455	1455	1521	1520
H063680006	53	53	50	50	100	100	500	500	1455	1455	1521	1520
H063680007	53	53	50	50	100	100	500	500	1455	1455	1521	1520
H063740003	53	53	50	50	100	100	500	500	1455	1455	1521	1521
H063740018	53	53	50	50	100	100	500	500	1455	1455	1521	1520
H063780007	53	53	50	50	100	100	500	500	1455	1455	1521	1521
H063780008	53	53	50	50	100	100	500	500	1455	1455	1521	1521
H063860003	53	53	50	50	100	100	500	500	1455	1455	1521	1521
H063960001	53	53	50	50	100	100	500	500	1455	1455	1521	1521
LC5759	53	53	50	50	100	100	500	500	1455	1455	1521	1519
H070420013	53	53	50	50	100	100	500	500	1455	1455	1521	1518
LC5822	53	53	50	50	100	100	500	500	1455	1455	1521	1520
H040260015	53	53	50	50	100	100	500	500	1455	1455	1521	1520
H055140095	53	53	50	50	100	100	500	500	1455	1455	1521	1520
H060780053	53	53	50	50	100	100	500	500	1455	1455	1521	1520
H061120064	53	53	50	50	100	100	500	500	1455	1455	1521	1520
H062840608	53	53	50	50	100	100	500	500	1455	1455	1521	1520
H062940111	53	53	50	50	100	100	500	500	1455	1455	1521	1520
H064320006	53	53	50	50	100	100	500	500	1455	1455	1521	1520
H064280005	53	53	50	50	100	100	500	500	1455	1455	1521	1521
H064380002	53	53	50	50	100	100	500	500	1455	1455	1521	1520
H064380001	53	53	50	50	100	100	500	500	1455	1455	1521	1520
H064560527	53	53	50	50	100	100	500	500	1455	1455	1521	1520
H064660638	53	53	50	50	100	100	500	500	1455	1455	1521	1521
H070160015	53	53	50	50	100	100	500	500	1455	1455	1521	1521
H071120010	53	53	50	50	100	100	500	500	1455	1455	1521	1520
H071360036	53	53	50	50	100	100	500	500	1455	1455	1521	1519
H072740002	53	53	50	50	100	100	500	500	1455	1455	1521	1521
H073000045	53	53	50	50	100	100	500	500	1455	1455	1521	1520
H073380007	53	53	50	50	100	100	500	500	1455	1455	1521	1520
H073600182	53	53	50	50	100	100	500	500	1455	1455	1521	1519
H073640185	53	53	50	50	100	100	500	500	1455	1455	1521	1521
H074960018	53	52	50	50	100	100	500	500	1455	1455	1521	1519
H080780059	53	53	50	50	100	100	500	500	1455	1455	1521	1521
H053840008	53	53	50	50	100	100	500	500	1455	1455	1521	1520
H072520002	53	53	50	50	100	100	500	500	1455	1455	1521	1520
H081340222	53	53	50	50	100	100	500	500	1455	1455	1521	1520
H082520613	53	53	50	50	100	100	500	500	1455	1455	1521	1520

H083120262	53	53	50	50	100	100	500	500	1455	1455	1521	1520
H083620580	53	52	50	50	100	100	500	500	1455	1455	1521	1518
H083960064	53	53	50	50	100	100	500	500	1455	1455	1521	1520
H084620118	53	53	50	50	100	100	500	499	1455	1454	1521	1518
H090140214	53	53	50	50	100	100	500	500	1455	1455	1521	1521
H090440226	53	52	50	50	100	100	500	500	1455	1455	1521	1519
H040960441	53	53	50	50	100	100	500	500	1455	1455	1521	1520
H041120007	53	53	50	50	100	100	500	500	1455	1455	1521	1520
H093480403	53	53	50	50	100	100	500	500	1455	1455	1521	1520
H094340202	53	53	50	50	100	100	500	500	1455	1455	1521	1519
H095060125	53	53	50	50	100	100	500	500	1455	1455	1521	1521
H100140151	53	53	50	50	100	100	500	500	1455	1455	1521	1519
H100660110	53	53	50	50	100	100	500	500	1455	1455	1521	1520
H100700025	53	53	50	50	100	100	500	500	1455	1455	1521	1520
H103140121	53	52	50	50	100	100	500	500	1455	1455	1521	1517
H103620160	53	53	50	50	100	100	500	500	1455	1455	1521	1521
H103660126	53	52	50	50	100	100	500	500	1455	1455	1521	1519
H103660121	53	53	50	50	100	100	500	500	1455	1455	1521	1521
H104420240	53	53	50	50	100	100	500	500	1455	1455	1521	1521
H110480273	53	53	50	50	100	100	500	500	1455	1455	1521	1519
H112320437	53	53	50	50	100	100	500	500	1455	1455	1521	1518
H112080616	53	53	50	50	100	100	500	500	1455	1455	1521	1520
H112380374	53	53	50	50	100	100	500	500	1455	1455	1521	1518
H120160499	53	53	50	50	100	100	500	500	1455	1455	1521	1521
H120200371	53	53	50	50	100	100	500	500	1455	1455	1521	1520
H105140391	53	53	50	50	100	100	500	500	1455	1455	1521	1520
H121040204	53	53	50	50	100	100	500	500	1455	1455	1521	1520
H121420445	53	53	50	50	100	100	500	500	1455	1455	1521	1520
H102240357	53	52	50	50	100	100	500	500	1455	1455	1521	1519
H122500497	53	53	50	50	100	100	500	500	1455	1455	1521	1519
H122820408	53	53	50	50	100	100	500	500	1455	1455	1521	1521
H123620597	53	52	50	50	100	100	500	500	1455	1455	1521	1519
H123840629	53	53	50	50	100	100	500	500	1455	1455	1521	1520
H123940534	53	53	50	50	100	100	500	500	1455	1455	1521	1519
H124920387	53	53	50	50	100	100	500	500	1455	1455	1521	1521
H131340777	53	53	50	50	100	100	500	500	1455	1455	1521	1519
H131480353	53	53	50	50	100	100	500	500	1455	1455	1521	1518
H131480354	53	52	50	50	100	100	500	500	1455	1455	1521	1518
H131840211	53	53	50	50	100	100	500	500	1455	1455	1521	1520
H131460248	53	53	50	50	100	100	500	500	1455	1455	1521	1520
H132140863	53	53	50	50	100	100	500	500	1455	1455	1521	1519
H053640534/EUL 168	53	53	50	50	100	100	500	500	1455	1455	1521	1521
H064180002	53	53	50	50	100	100	500	500	1455	1455	1521	1521
H064180019	53	53	50	50	100	100	500	500	1455	1455	1521	1519
H043540106	53	53	50	50	100	100	500	500	1455	1455	1521	1518
H044120014	53	53	50	50	100	100	500	500	1455	1455	1521	1521
H052780022	53	53	50	50	100	100	500	500	1455	1455	1521	1521
H054280040	53	53	50	50	100	100	500	500	1455	1455	1521	1515
H063680003	53	53	50	50	100	100	500	500	1455	1455	1521	1519
H063840008	53	53	50	50	100	100	500	500	1455	1455	1521	1521

H073660582	53	53	50	50	100	100	500	500	1455	1455	1521	1521
LC5804	53	53	50	50	100	100	500	500	1455	1455	1521	1520
H063760005	53	53	50	50	100	100	500	500	1455	1455	1521	1521
H064240003	53	53	50	50	100	100	500	500	1455	1455	1521	1520
H065040012	53	53	50	50	100	100	500	500	1455	1455	1521	1520
H070140635	53	53	50	50	100	100	500	500	1455	1455	1521	1517
H073020039	53	53	50	50	100	100	500	500	1455	1455	1521	1520
H073320399	53	53	50	50	100	100	500	500	1455	1455	1521	1519
H073440003	53	53	50	50	100	100	500	500	1455	1455	1521	1520
LC6009	53	53	50	50	100	100	500	500	1455	1454	1521	1519
H083140015	53	53	50	50	100	100	500	500	1455	1455	1521	1520
H093400182	53	53	50	50	100	100	500	500	1455	1455	1521	1521
H094760070	53	53	50	50	100	100	500	500	1455	1455	1521	1520
H094800237	53	53	50	50	100	100	500	500	1455	1455	1521	1519
H110480715	53	53	50	50	100	100	500	500	1455	1455	1521	1519
H112840293	53	53	50	50	100	100	500	500	1455	1455	1521	1521
H114100406	53	53	50	50	100	100	500	500	1455	1455	1521	1521
H120240362	53	53	50	50	100	100	500	500	1455	1455	1521	1521
H104640262	53	52	50	50	100	100	500	500	1455	1455	1521	1519
H123140428	53	53	50	50	100	100	500	500	1455	1455	1521	1520
H123460520	53	53	50	50	100	100	500	500	1455	1455	1521	1521
H124360642	53	53	50	50	100	100	500	500	1455	1455	1521	1521
Pontiac-1	53	53	50	50	100	100	499	495	1444	1437	1507	1501

Table S12. 61 untypable genes in the six extended MLST schemes and the number of affected isolates in the typing panel.

**Gene**	**Scheme(s)**	**Number of affected isolates in typing panel**
lpg0328	cgMLST (1521), rMLST	10
lpg1614	cgMLST (1521)	9
lpg0703	cgMLST (1521)	5
lpg1615	cgMLST (1521)	5
lpg0568	cgMLST (1455)	4
lpg2871	cgMLST (1521)	4
lpg0639	cgMLST (1521)	3
lpg0857	cgMLST (1521)	3
lpg1125	cgMLST (1521)	3
lpg1612	cgMLST (1455), cgMLST (1521)	3
lpg1872	cgMLST (1521)	3
lpg2121	cgMLST (1521)	3
lpg2361	cgMLST (1521)	3
lpg2422	cgMLST (1521)	3
lpg2452	cgMLST (1521)	3
lpg2888	cgMLST (1521)	3

lpg0179	cgMLST (1521)	2
lpg0735	cgMLST (1521)	2
lpg0744	cgMLST (1521)	2
lpg0903	cgMLST (1521)	2
lpg1099	cgMLST (1521)	2
lpg1100	cgMLST (1521)	2
lpg1169	cgMLST (1521)	2
lpg1371	cgMLST (1521)	2
lpg1664	cgMLST (1521)	2
lpg0049	cgMLST (1521)	1
lpg0073	cgMLST (1521)	1
lpg0121	cgMLST (1521)	1
lpg0286	cgMLST (1521)	1
lpg0316	cgMLST (1521)	1
lpg0326	cgMLST (1521)	1
lpg0329	cgMLST (50), cgMLST (100), cgMLST (500), cgMLST (1455), cgMLST (1521), rMLST	1
lpg0342	cgMLST (500), cgMLST (1455), cgMLST (1521), rMLST	1
lpg0409	cgMLST (50), cgMLST (100), cgMLST (500), cgMLST (1455), cgMLST (1521)	1
lpg0549	cgMLST (1521)	1
lpg0707	cgMLST (1521)	1
lpg0952	cgMLST (1521)	1
lpg1181	cgMLST (1521)	1
lpg1187	cgMLST (1521)	1
lpg1199	cgMLST (1521)	1
lpg1209	cgMLST (1521)	1
lpg1335	cgMLST (1521)	1
lpg1564	cgMLST (1455)	1
lpg1567	cgMLST (1455)	1
lpg1581	cgMLST (1521)	1
lpg1665	cgMLST (1521)	1
lpg1751	cgMLST (1455), cgMLST (1521)	1
lpg1868	cgMLST (1521)	1
lpg2016	cgMLST (1521)	1
lpg2044	cgMLST (1455), cgMLST (1521)	1
lpg2146	cgMLST (1521)	1
lpg2196	cgMLST (1521)	1
lpg2208	cgMLST (500), cgMLST (1455), cgMLST (1521)	1
lpg2227	cgMLST (1521)	1
lpg2395	cgMLST (1521)	1
lpg2446	cgMLST (1521)	1
lpg2462	cgMLST (1521)	1
lpg2506	cgMLST (500), cgMLST (1455), cgMLST (1521)	1
lpg2639	cgMLST (1521)	1
lpg2856	cgMLST (1521)	1
lpg2984	cgMLST (1521)	1

Table S13. The mean and range of mapping coverage, number of contigs and N50 values of isolates that produce complete or incomplete profiles in the extended MLST schemes.

	**Mean (and range) of mapping depth**	**Mean (and range) of contigs**	**Mean (and range) of N50 values (bp)**
Isolates with a full profile in all extended MLST schemes	125.4x (77.1x-162.3x)	35.2 (17-72)	246,381 (101,986-657,238)
Isolates with an incomplete profile in 1 or more extended MLST schemes	121.8x (71.4x-164.2x)	35.0 (15-65)	264,222 (86,373-726,453)
Significant difference via unpaired t-test?	No significant difference	No significant difference	No significant difference

Table S14. The number of accessory genes scored as present, absent or untypable using the gene presence/absence typing method.

**EUL/isolate number**	**No. genes present**	**No. genes absent**	**No. untypable genes**
1	97	103	0
2	50	148	2
3	97	103	0
4	116	82	2
6	85	112	3
7	117	82	1
8	87	110	3
13	104	93	3
14	104	93	3
16	106	93	1
17	92	108	0
18	37	163	0
19	66	131	3
20	59	139	2
25	55	145	0
26	59	141	0
27	102	96	2
27 (replicate)	102	96	2
28	100	97	3
29	99	101	0
30	62	136	2
31	101	99	0
32	90	109	1
33	62	137	1

33 (replicate)	62	136	2
36	87	113	0
37	92	108	0
38	97	103	0
39	61	138	1
40	30	168	2
41	88	110	2
42	97	103	0
43	96	103	1
48	108	92	0
49	96	103	1
50	86	112	2
51	41	156	3
52	54	143	3
53	96	103	1
54	104	96	0
55	97	103	0
60	96	102	2
63	80	118	2
66	80	118	2
67	96	103	1
68	84	115	1
69	70	130	0
69 (replicate)	69	129	2
70	102	98	0
71	115	85	0
72	47	151	2
73	69	129	2
74	61	137	2
75	87	112	1
75 (replicate)	87	112	1
81	66	132	2
82	101	99	0
83	62	136	2
84	96	104	0
85	101	99	0
86	108	92	0
87	73	124	3
88	101	99	0
91	28	169	3
92	65	132	3
92 (replicate)	66	133	1
93	101	99	0
97	52	144	4
98	52	143	5
99	52	146	2

100	81	119	0
101	85	112	3
102	75	123	2
103	107	92	1
104	107	93	0
105	87	113	0
110	106	94	0
111	59	141	0
111 (replicate)	59	140	1
114	91	109	0
116	58	141	1
117	92	107	1
118	68	132	0
119	97	103	0
120	87	112	1
9	97	103	0
10	97	103	0
11	88	110	2
12	88	110	2
22	53	146	1
23	53	145	2
24	53	146	1
34	62	136	2
35	62	135	3
44	95	105	0
45	97	103	0
46	97	103	0
47	30	168	2
56	108	92	0
57	103	95	2
58	97	103	0
59	42	158	0
76	115	85	0
77	115	85	0
78	69	129	2
79	69	129	2
94	101	99	0
95	101	99	0
96	66	132	2
106	53	144	3
107	52	143	5
121	87	112	1
LC 202/EUL 153	66	132	2
LC 206/EUL 158	66	132	2
LC 569/EUL 154	69	131	0
LC 606/EUL 155	69	131	0

LC 384/EUL 156	66	132	2
LC 395/EUL 159	66	132	2
LC6379-1/EUL 145	74	126	0
LC6376	73	125	2
LC6382	74	126	0
LC6391	74	126	0
LC6394	73	126	1
LC6397	74	126	0
LC6406	74	126	0
LC6407	74	126	0
LC6408	74	126	0
LC6411	74	126	0
LC6412	73	126	1
LC6413	73	126	1
LC6416	74	126	0
LC6418	74	126	0
LC6385	74	126	0
LC6388	74	126	0
LC6409	74	126	0
LC6410	73	125	2
LC0537/EUL 132	69	129	2
LC0539/EUL 133	69	129	2
LC0540/EUL 134	69	129	2
LC0565	69	129	2
LC0583	69	129	2
H034680033	71	124	5
H034680035/EUL 165	73	125	2
H034690056/EUL 166	73	126	1
H034800427	73	125	2
H034980467	72	125	3
Paris	92	107	1
H034800423	95	102	3
OLDA1 (NCTC12008)	97	101	2
EUL 109	107	93	0
H064240448	69	129	2
LC0731	62	136	2
LC0732	62	136	2
LC0763	62	136	2
LC0782	62	136	2
LC0795	62	136	2
LC0801	62	136	2
LC5694	67	132	1
LC5722	55	143	2
LC5738	67	133	0
LC5755	69	129	2
LC6163	70	130	0

LC6267	69	129	2
LC6268	69	129	2
LC6228	73	125	2
H041380048	69	129	2
H041640791	69	128	3
H042960010	69	129	2
H061140013	69	129	2
H071880001	69	129	2
H073060003	69	129	2
H080820009	84	114	2
LC6058	69	129	2
LC6293	69	129	2
LC6788	69	129	2
H062660463	69	129	2
H073900557	79	119	2
LC1127	62	136	2
H084760449	73	125	2
H085020185	73	125	2
H090320386	69	129	2
H044260061	72	125	3
H093140322	73	125	2
H093160422	73	125	2
H092760433	69	129	2
H100940111	69	129	2
H101760092	69	129	2
H101820190	69	129	2
H102020414	72	126	2
H101980130	69	129	2
H103820081	68	129	3
H120240685	69	129	2
H104320293	69	130	1
H113180118	67	132	1
H113340664	67	132	1
H113280076	78	120	2
H113660550	70	128	2
H114740454	69	129	2
H115040456	69	129	2
H111580389	73	125	2
H113780240	69	129	2
H083920177	73	125	2
H084140691	69	129	2
H081180019	69	128	3
H103260667	78	122	0
LC464	69	129	2
LC0512	69	130	1
LC0794	69	129	2

LC0798	69	129	2
LC0536/EUL 131	69	125	6
LC230/EUL 122	89	110	1
LC231/EUL 123	89	111	0
LC0462/EUL 124	87	112	1
LC0463/EUL 125	87	112	1
Lorraine	87	113	0
EUL 169	101	97	2
H064160534	102	98	0
H064160538/EUL 170	101	98	1
H034700617	100	97	3
H043580159	102	98	0
H043580160	102	98	0
H043660021	102	98	0
H043680663	102	98	0
H043700021	111	89	0
H043790008	102	97	1
H052920051	101	99	0
H053540106	102	98	0
H063660005	101	97	2
H063660006	101	97	2
H063760006	100	97	3
H063660009	102	97	1
H063680006	107	89	4
H063680007	101	96	3
H063740003	101	97	2
H063740018	102	97	1
H063780007	102	98	0
H063780008	102	98	0
H063860003	98	96	6
H063960001	100	96	4
LC5759	102	98	0
H070420013	102	98	0
LC5822	102	98	0
H040260015	100	97	3
H055140095	101	97	2
H060780053	102	97	1
H061120064	101	97	2
H062840608	100	98	2
H062940111	102	98	0
H064320006	99	98	3
H064280005	102	97	1
H064380002	102	98	0
H064380001	102	97	1
H064560527	101	97	2
H064660638	102	98	0

H070160015	102	98	0
H071120010	101	97	2
H071360036	101	97	2
H072740002	102	97	1
H073000045	101	97	2
H073380007	102	98	0
H073600182	102	98	0
H073640185	102	97	1
H074960018	101	97	2
H080780059	101	97	2
H053840008	102	98	0
H072520002	102	97	1
H081340222	101	97	2
H082520613	101	97	2
H083120262	102	98	0
H083620580	102	97	1
H083960064	102	97	1
H084620118	102	98	0
H090140214	102	98	0
H090440226	102	97	1
H040960441	101	97	2
H041120007	102	98	0
H093480403	102	98	0
H094340202	102	98	0
H095060125	102	98	0
H100140151	102	98	0
H100660110	102	98	0
H100700025	102	98	0
H103140121	102	98	0
H103620160	101	97	2
H103660126	102	97	1
H103660121	102	98	0
H104420240	102	98	0
H110480273	102	98	0
H112320437	102	98	0
H112080616	102	98	0
H112380374	102	98	0
H120160499	102	98	0
H120200371	100	97	3
H105140391	102	98	0
H121040204	102	98	0
H121420445	102	98	0
H102240357	102	98	0
H122500497	101	98	1
H122820408	101	99	0
H123620597	88	112	0

H123840629	102	98	0
H123940534	102	98	0
H124920387	102	98	0
H131340777	102	98	0
H131480353	102	98	0
H131480354	103	97	0
H131840211	102	98	0
H131460248	102	98	0
H132140863	100	98	2
H053640534/EUL 168	102	98	0
H064180002	77	122	1
H064180019	77	122	1
H043540106	104	96	0
H044120014	112	88	0
H052780022	114	86	0
H054280040	115	85	0
H063680003	124	75	1
H063840008	115	85	0
H073660582	124	76	0
LC5804	126	74	0
H063760005	103	97	0
H064240003	103	95	2
H065040012	104	96	0
H070140635	115	85	0
H073020039	115	85	0
H073320399	114	85	1
H073440003	113	86	1
LC6009	104	96	0
H083140015	104	96	0
H093400182	113	87	0
H094760070	124	76	0
H094800237	124	76	0
H110480715	114	86	0
H112840293	124	76	0
H114100406	104	96	0
H120240362	124	76	0
H104640262	82	118	0
H123140428	114	86	0
H123460520	121	79	0
H124360642	124	76	0
Pontiac-1	113	84	3

Table S15. 21 genes that were scored as untypable in one or more typing panel isolates using the gene presence/absence typing method and the number of affected typing panel isolates.

**Gene number**	**Number of affected isolates in typing panel**
130	38
87	36
187	10
91	4
81	4
47	4
39	4
133	3
31	3
49	2
46	2
45	2
163	2
171	2
108	2
155	1
172	1
18	1
28	1
123	1
128	1

Table S16. The number of differences identified between isolates from epidemiologically "related" sets in the typing panel using each of the WGS-based methods.
Sets in which one or more isolates could not be fully typed by a particular scheme are marked with an asterisk.

**EUL numbers**	**Mean (and range) of pairwise differences**							
	**SNP-based**	**rMLST (53)**	**cgMLST (50)**	**cgMLST (100)**	**cgMLST (500)**	**cgMLST (1455)**	**cgMLST (1521)**	**Gene pres./ abs.**	**Kmer-based**
	SNPs	*Alleles*	*Alleles*	*Alleles*	*Alleles*	*Alleles*	*Alleles*	*Genes*	*Jaccard distance*
Typing panel subdivision I sets ("definitely related")									
48, 56	0	0*	0	0	0	0	0*	0	0.065
71, 76, 77	0.67 (0-1)	0 (0-0)	0 (0-0)	0.67 (0-1)	0.67 (0-1)	0.67 (0-1)	0.67 (0-1)*	0 (0-0)	0.065 (0.065-

									0.065)
73, 78, 79	0 (0-0)	0 (0-0)	0 (0-0)	0 (0-0)	0 (0-0)	0 (0-0)	0 (0-0)*	0 (0-0)*	0.064 (0.061-0.065)
120, 121	0	0*	0	0	0	0	0	0*	0.0646
Typing panel subdivision II sets ("probably related")									
3, 9, 10	2.67 (0-4)	0 (0-0)	0 (0-0)	0 (0-0)	0.67 (0-1)	1.33 (0-2)	2.67 (1-4)*	0 (0-0)	0.065 (0.064-0.065)
8, 11, 12	10.33 (0-16)	0 (0-0)	0 (0-0)	0 (0-0)	2 (0-3)	5.33 (0-8)	8.67 (1-13)*	0 (0-0)*	0.065 (0.065-0.065)
19, 22, 23, 24	20.67 (0-50)	0 (0-0)	0 (0-0)	0 (0-0)	0.5 (0-1)	1.5 (0-3)	4 (0-8)*	24.5 (0-49)*	0.10 (0.057-0.14)
33, 34, 35	1 (0-2)	0 (0-0)	0 (0-0)	0 (0-0)	0 (0-0)*	0 (0-0)*	3.67 (3-5)*	0 (0-0)*	0.064 (0.063-0.065)
37, 44, 45	1915 (179-2786)	0 (0-0)	3.33 (1-5)	4.67 (1-7)	24 (6-35)	58 (13-82)	60 (9-87)*	4.67 (0-7)	0.11 (0.10-0.13)
38, 46	5	0	0	1	2	3	5*	0	0.064
40, 47	0	0	0	0	0	0	0*	0*	0.054
51, 59	15	0	0	0	1	8*	8*	0*	0.066
54, 57	2	0	0	0	0*	0*	1*	0*	0.063
55, 58	0	0	0	0	0	0	0	0	0.063
81, 96	6	0	0	0	0	1	2*	0*	0.059
93, 94, 95	0.67 (0-1)	0 (0-0)	0 (0-0)	0 (0-0)	0 (0-0)	0 (0-0)*	0 (0-0)*	0 (0-0)	0.065 (0.065-0.065)
97, 106, 107	0 (0-0)	0 (0-0)*	0 (0-0)	0 (0-0)	0 (0-0)	0 (0-0)	0 (0-0)*	0 (0-0)*	0.065 (0.064-0.065)

Table S17. The number of differences between isolates belonging to an additional 20 epidemiologically "related" sets, as analysed by each of the WGS-based methods.
Sets in which one or more isolates could not be fully typed by a particular scheme are marked with an asterisk.

**Isolate names**	**Mean (and range) of pairwise differences**								
	SNP-based	rMLST (53)	cgMLST (50)	cgMLST (100)	cgMLST (500)	cgMLST (1455)	cgMLST (1521)	Gene pres./abs.	Kmer-based
	SNPs	*Alleles*	*Alleles*	*Alleles*	*Alleles*	*Alleles*	*Alleles*	*Genes*	*Jaccard distance*
EUL 153/ EUL 158	0	0	0	0	0	0	0*	0*	0.00017
EUL 154/ EUL 155	3	0	0	0	0	1	2	0	0.00020
EUL 156/ EUL 159	2	0	0	0	0	1	1*	0*	0.00065
Barrow outbreak (*n*=18)	0.48 (0-2)	0 (0-0)	0 (0-0)	0 (0-0)	0 (0-0)	0 (0-0)	0 (0-0)*	0 (0-0)*	0.00052 (0.00030-0.00075)
BBC outbreak	1 (0-2)	0 (0-0)	0 (0-0)	0 (0-0)	0 (0-0)	0.4	0.4	0 (0-0)*	0.00052

(*n*=5)						(0-1)*	(0-1)*		(0.00039-0.00071)
Hereford outbreak (*n*=5)	4 (0-9)	0(0-0)*	0 (0-0)*	0 (0-0)*	0.4 (0-1)*	0.8 (0-2)*	1.6 (0-4)*	0 (0-0)*	0.0061 (0.00044-0.13)
LC0731/ LC0732/ LC0763/ LC0782/ LC0795/ LC0801	0.33 (0-1)	0 (0-0)*	0 (0-0)	0 (0-0)	0 (0-0)	0.33 (0-1)	0 (0-0)*	0 (0-0)*	0.064 (0.063-0.065)
H041380048/ H041640791	4	0	0	0	1	1	1	0*	0.066
H093140322/ H093160422	4	1	0	0	0	1	3	0*	0.060
H113180118/ H113340664	1	0	0	0	0	0	1*	0*	0.064
H083920177/ H084140691	5	0	0	0	0	4*	6*	4*	0.075
LC0794/ LC0798	1	0	0	0	0	1*	1*	0*	0.065
EUL 122/ EUL 123	0	0	0	0	0	0	0*	0*	0.062
EUL 124/ EUL 125	2	0	0	0	0	1*	0*	0*	0.064
EUL 169/ H064160534/ EUL 170	1.33 (1-2)	0 (0-0)	0 (0-0)	0 (0-0)	0 (0-0)	0 (0-0)	0.67 (0-1)*	0 (0-0)*	0.064 (0.062-0.064)
H063660005/ H063660006/ H063760006	3.33 (2-5)	0 (0-0)	0 (0-0)	0 (0-0)	0 (0-0)	1.33 (1-2)	0.67 (0-1)*	0 (0-0)*	0.064 (0.061-0.065)
H063680006/ H063680007	11	0	0	0	1	5	7*	10*	0.076
H063780007/ H063780008	2	0	0	0	0	2	1	0	0.064
H131340777/ H131480353/ H131480354/ H131840211	2.17 (0-4)	0 (0-0)*	0 (0-0)	0.5 (0-1)	1.5 (0-3)	2.17 (0-4)	1 (0-2)*	0.5 (0-1)	0.073 (0.061-0.080)
H064180002/ H064180019	1	0	0	0	0	0	1*	0*	0.065

Table S18. The indices of discrimination (*D*) for 53 ribosomal genes, calculated using 79 epidemiologically “unrelated” isolates from the typing panel.

**Gene name**	***D* value**
lpg0342/rpsN	0.728
lpg0343/rpsH	0.846
lpg0344/rplF	0.853
lpg0345/rplR	0.586
lpg0346/rpsE	0.771
lpg0347/rpmD	0.611
lpg0348/rplO	0.844
lpg0350/rpmJ	0.025
lpg0351/rpsM	0.677
lpg0352/rpsK	0.758

lpg0353/rpsD	0.873
lpg0355/rplQ	0.848
lpg0395/rplS	0.801
lpg0399/rpsP	0.542
lpg0478/rpmG	0.607
lpg0479/rpmB	0.802
lpg0650/rpmE	0.520
lpg1391/rpmF	0.625
lpg1421/rpsA	0.866
lpg1589/rplI	0.836
lpg1591/rpsR	0.730
lpg1592/rpsF	0.808
lpg1714/rpsB	0.858
lpg2358/rpsU	0.249
lpg2636/rpsT	0.561
lpg2650/rpmA	0.075
lpg2651/rplU	0.678
lpg2706/rpsI	0.802
lpg2707/rplM	0.677
lpg2712/rplT	0.525
lpg2769/rpsO	0.824
lpg3005/rpmH	0.516
Unannotated/*rpmI*	0.075

Table S19. The indices of discrimination (*D*) for 200 accessory genes, calculated using 79 epidemiologically "unrelated" isolates from the typing panel.

**Gene number**	***D* value**
1	0.500
2	0.142
3	0.418
4	0.504
5	0.356
6	0.483
7	0.483
8	0.493
9	0.506
10	0.488
11	0.483
12	0.477
13	0.477
14	0.477

15	0.477
16	0.342
17	0.483
18	0.504
19	0.504
20	0.488
21	0.500
22	0.342
23	0.327
24	0.327
25	0.327
26	0.327
27	0.327
28	0.327
29	0.327
30	0.327
31	0.471
32	0.356
33	0.356
34	0.184
35	0.506
36	0.477
37	0.506
38	0.500
39	0.383
40	0.327
41	0.327
42	0.395
43	0.502
44	0.327
45	0.327
46	0.327
47	0.327
48	0.327
49	0.327
50	0.295
51	0.295
52	0.295
53	0.327
54	0.243
55	0.502
56	0.500
57	0.500
58	0.312
59	0.295
60	0.383
61	0.506

62	0.506
63	0.506
64	0.506
65	0.463
66	0.493
67	0.488
68	0.383
69	0.356
70	0.356
71	0.356
72	0.493
73	0.383
74	0.483
75	0.488
76	0.493
77	0.295
78	0.497
79	0.483
80	0.506
81	0.488
82	0.497
83	0.204
84	0.477
85	0.477
86	0.477
87	0.500
88	0.261
89	0.261
90	0.261
91	0.463
92	0.502
93	0.502
94	0.356
95	0.483
96	0.456
97	0.500
98	0.497
99	0.497
100	0.497
101	0.497
102	0.497
103	0.497
104	0.497
105	0.497
106	0.477
107	0.025
108	0.312

109	0.477
110	0.504
111	0.504
112	0.327
113	0.493
114	0.477
115	0.502
116	0.477
117	0.438
118	0.074
119	0.463
120	0.506
121	0.164
122	0.506
123	0.312
124	0.493
125	0.506
126	0.504
127	0.243
128	0.471
129	0.506
130	0.493
131	0.504
132	0.504
133	0.502
134	0.504
135	0.506
136	0.370
137	0.463
138	0.456
139	0.502
140	0.278
141	0.428
142	0.447
143	0.447
144	0.395
145	0.418
146	0.418
147	0.447
148	0.395
149	0.261
150	0.261
151	0.261
152	0.261
153	0.356
154	0.383
155	0.488

156	0.488
157	0.477
158	0.395
159	0.502
160	0.493
161	0.074
162	0.224
163	0.164
164	0.261
165	0.327
166	0.204
167	0.477
168	0.164
169	0.383
170	0.327
171	0.295
172	0.025
173	0.506
174	0.000
175	0.050
176	0.025
177	0.502
178	0.504
179	0.025
180	0.050
181	0.500
182	0.164
183	0.356
184	0.502
185	0.456
186	0.383
187	0.483
188	0.074
189	0.502
190	0.164
191	0.504
192	0.295
193	0.483
194	0.025
195	0.395
196	0.471
197	0.502
198	0.395
199	0.488
200	0.164

Table S20. The indices of discrimination (D) for 1455 core genes, calculated using 79 epidemiologically "unrelated" isolates from the typing panel.

**Gene name**	***D* value**		**Gene name**	***D* value**		**Gene name**	***D* value**
lpg0001	0.862		*lpg0930*	0.733		*lpg2013*	0.873
lpg0002	0.866		*lpg0932*	0.850		*lpg2014*	0.871
lpg0004	0.866		*lpg0933*	0.837		*lpg2015*	0.869
lpg0005	0.881		*lpg0934*	0.846		*lpg2017*	0.859
lpg0009	0.278		*lpg0935*	0.769		*lpg2018*	0.868
lpg0010	0.880		*lpg0936*	0.829		*lpg2020*	0.858
lpg0011	0.879		*lpg0937*	0.887		*lpg2021*	0.881
lpg0014	0.880		*lpg0938*	0.838		*lpg2023*	0.879
lpg0018	0.878		*lpg0940*	0.874		*lpg2024*	0.879
lpg0021	0.835		*lpg0941*	0.864		*lpg2025*	0.868
lpg0022	0.901		*lpg0942*	0.849		*lpg2027*	0.880
lpg0023	0.852		*lpg0943*	0.840		*lpg2028*	0.870
lpg0024	0.793		*lpg0946*	0.809		*lpg2029*	0.784
lpg0025	0.883		*lpg0949*	0.840		*lpg2031*	0.873
lpg0027	0.894		*lpg0950*	0.849		*lpg2032*	0.870
lpg0028	0.896		*lpg0951*	0.861		*lpg2033*	0.873
lpg0032	0.885		*lpg0953*	0.845		*lpg2034*	0.880
lpg0033	0.862		*lpg0954*	0.870		*lpg2036*	0.879
lpg0035	0.731		*lpg0955*	0.861		*lpg2037*	0.874
lpg0037	0.870		*lpg0956*	0.846		*lpg2038*	0.805
lpg0040	0.866		*lpg0957*	0.872		*lpg2039*	0.896
lpg0043	0.862		*lpg0958*	0.864		*lpg2040*	0.889
lpg0047	0.875		*lpg0960*	0.829		*lpg2041*	0.856
lpg0048	0.882		*lpg0961*	0.847		*lpg2042*	0.878
lpg0052	0.877		*lpg0962*	0.858		*lpg2043*	0.806
lpg0059	0.867		*lpg0963*	0.853		*lpg2044*	0.857
lpg0075	0.843		*lpg0966*	0.857		*lpg2045*	0.875
lpg0076	0.857		*lpg0970*	0.848		*lpg2046*	0.872
lpg0078	0.874		*lpg0971*	0.858		*lpg2047*	0.871
lpg0079	0.873		*lpg1117*	0.862		*lpg2048*	0.873
lpg0083	0.864		*lpg1119*	0.874		*lpg2049*	0.782
lpg0084	0.873		*lpg1121*	0.861		*lpg2051*	0.876
lpg0085	0.851		*lpg1122*	0.864		*lpg2052*	0.882
lpg0089	0.841		*lpg1131*	0.875		*lpg2053*	0.884
lpg0091	0.851		*lpg1135*	0.870		*lpg2175*	0.863
lpg0094	0.856		*lpg1136*	0.868		*lpg2176*	0.871
lpg0095	0.860		*lpg1137*	0.857		*lpg2178*	0.878
lpg0098	0.853		*lpg1138*	0.874		*lpg2186*	0.903
lpg0099	0.851		*lpg1139*	0.853		*lpg2187*	0.849
lpg0100	0.859		*lpg1140*	0.853		*lpg2189*	0.855
lpg0101	0.858		*lpg1141*	0.857		*lpg2191*	0.859

lpg0102	0.876		*lpg1143*	0.849		*lpg2193*	0.842
lpg0103	0.854		*lpg1144*	0.871		*lpg2194*	0.861
lpg0104	0.840		*lpg1146*	0.869		*lpg2200*	0.848
lpg0105	0.816		*lpg1147*	0.856		*lpg2201*	0.870
lpg0106	0.866		*lpg1148*	0.865		*lpg2202*	0.848
lpg0110	0.878		*lpg1154*	0.863		*lpg2203*	0.867
lpg0111	0.868		*lpg1155*	0.858		*lpg2204*	0.864
lpg0115	0.845		*lpg1157*	0.855		*lpg2206*	0.864
lpg0116	0.885		*lpg1159*	0.859		*lpg2207*	0.863
lpg0117	0.848		*lpg1161*	0.819		*lpg2208*	0.824
lpg0118	0.778		*lpg1162*	0.852		*lpg2209*	0.854
lpg0119	0.854		*lpg1164*	0.864		*lpg2210*	0.864
lpg0120	0.856		*lpg1165*	0.853		*lpg2211*	0.851
lpg0122	0.882		*lpg1166*	0.858		*lpg2212*	0.862
lpg0125	0.856		*lpg1167*	0.825		*lpg2213*	0.806
lpg0127	0.865		*lpg1171*	0.851		*lpg2214*	0.869
lpg0128	0.864		*lpg1172*	0.875		*lpg2220*	0.871
lpg0129	0.865		*lpg1174*	0.874		*lpg2222*	0.842
lpg0130	0.870		*lpg1176*	0.880		*lpg2225*	0.865
lpg0131	0.858		*lpg1178*	0.885		*lpg2228*	0.888
lpg0136	0.872		*lpg1179*	0.871		*lpg2229*	0.885
lpg0137	0.869		*lpg1180*	0.867		*lpg2231*	0.889
lpg0138	0.863		*lpg1186*	0.865		*lpg2232*	0.853
lpg0140	0.871		*lpg1188*	0.877		*lpg2233*	0.757
lpg0153	0.868		*lpg1189*	0.867		*lpg2234*	0.886
lpg0165	0.854		*lpg1190*	0.883		*lpg2235*	0.886
lpg0175	0.796		*lpg1191*	0.824		*lpg2238*	0.867
lpg0183	0.875		*lpg1195*	0.866		*lpg2240*	0.891
lpg0188	0.875		*lpg1196*	0.868		*lpg2242*	0.904
lpg0189	0.861		*lpg1197*	0.873		*lpg2243*	0.840
lpg0194	0.872		*lpg1198*	0.869		*lpg2245*	0.908
lpg0197	0.833		*lpg1202*	0.856		*lpg2246*	0.833
lpg0206	0.858		*lpg1203*	0.845		*lpg2247*	0.863
lpg0209	0.869		*lpg1205*	0.631		*lpg2248*	0.873
lpg0212	0.878		*lpg1206*	0.883		*lpg2249*	0.850
lpg0213	0.870		*lpg1207*	0.852		*lpg2250*	0.870
lpg0217	0.867		*lpg1208*	0.848		*lpg2255*	0.741
lpg0218	0.857		*lpg1212*	0.853		*lpg2256*	0.870
lpg0227	0.864		*lpg1214*	0.872		*lpg2258*	0.833
lpg0229	0.866		*lpg1215*	0.865		*lpg2259*	0.826
lpg0232	0.777		*lpg1216*	0.839		*lpg2260*	0.852
lpg0238	0.868		*lpg1217*	0.843		*lpg2261*	0.866
lpg0239	0.866		*lpg1218*	0.838		*lpg2262*	0.895
lpg0241	0.871		*lpg1219*	0.853		*lpg2263*	0.881
lpg0243	0.860		*lpg1220*	0.854		*lpg2264*	0.728
lpg0244	0.870		*lpg1221*	0.845		*lpg2266*	0.884

lpg0245	0.882		*lpg1225*	0.858		*lpg2267*	0.889
lpg0248	0.861		*lpg1226*	0.864		*lpg2271*	0.858
lpg0252	0.856		*lpg1276*	0.858		*lpg2272*	0.850
lpg0256	0.862		*lpg1277*	0.908		*lpg2273*	0.866
lpg0257	0.867		*lpg1278*	0.844		*lpg2274*	0.868
lpg0260	0.854		*lpg1279*	0.848		*lpg2275*	0.860
lpg0264	0.858		*lpg1280*	0.859		*lpg2276*	0.856
lpg0267	0.860		*lpg1281*	0.775		*lpg2277*	0.850
lpg0268	0.852		*lpg1282*	0.838		*lpg2278*	0.861
lpg0269	0.864		*lpg1283*	0.862		*lpg2279*	0.856
lpg0271	0.844		*lpg1284*	0.864		*lpg2280*	0.870
lpg0276	0.874		*lpg1285*	0.858		*lpg2281*	0.857
lpg0282	0.893		*lpg1286*	0.851		*lpg2282*	0.853
lpg0287	0.883		*lpg1287*	0.852		*lpg2285*	0.853
lpg0288	0.893		*lpg1288*	0.771		*lpg2295*	0.855
lpg0289	0.896		*lpg1291*	0.853		*lpg2297*	0.868
lpg0290	0.893		*lpg1292*	0.848		*lpg2298*	0.857
lpg0291	0.876		*lpg1293*	0.818		*lpg2299*	0.872
lpg0293	0.902		*lpg1294*	0.858		*lpg2300*	0.854
lpg0294	0.896		*lpg1296*	0.858		*lpg2302*	0.834
lpg0295	0.881		*lpg1297*	0.837		*lpg2303*	0.820
lpg0296	0.889		*lpg1298*	0.765		*lpg2304*	0.883
lpg0298	0.877		*lpg1300*	0.852		*lpg2306*	0.848
lpg0299	0.885		*lpg1301*	0.857		*lpg2307*	0.828
lpg0301	0.876		*lpg1302*	0.852		*lpg2310*	0.889
lpg0317	0.857		*lpg1303*	0.843		*lpg2312*	0.763
lpg0318	0.850		*lpg1304*	0.859		*lpg2313*	0.855
lpg0319	0.876		*lpg1305*	0.853		*lpg2314*	0.850
lpg0320	0.704		*lpg1306*	0.858		*lpg2315*	0.604
lpg0321	0.716		*lpg1307*	0.858		*lpg2316*	0.858
lpg0322	0.900		*lpg1319*	0.818		*lpg2317*	0.867
lpg0323	0.879		*lpg1320*	0.885		*lpg2318*	0.861
lpg0324	0.294		*lpg1323*	0.849		*lpg2319*	0.858
lpg0325	0.688		*lpg1324*	0.861		*lpg2320*	0.863
lpg0329	0.851		*lpg1331*	0.864		*lpg2321*	0.883
lpg0330	0.855		*lpg1332*	0.833		*lpg2322*	0.870
lpg0331	0.566		*lpg1333*	0.873		*lpg2323*	0.870
lpg0332	0.776		*lpg1334*	0.849		*lpg2325*	0.871
lpg0335	0.767		*lpg1336*	0.847		*lpg2327*	0.877
lpg0336	0.844		*lpg1337*	0.848		*lpg2328*	0.834
lpg0337	0.227		*lpg1338*	0.853		*lpg2331*	0.862
lpg0338	0.288		*lpg1339*	0.837		*lpg2333*	0.870
lpg0339	0.830		*lpg1340*	0.866		*lpg2334*	0.796
lpg0340	0.844		*lpg1341*	0.841		*lpg2335*	0.866
lpg0341	0.780		*lpg1342*	0.857		*lpg2336*	0.867
lpg0342	0.724		*lpg1344*	0.847		*lpg2337*	0.854

lpg0343	0.846		*lpg1346*	0.845		*lpg2338*	0.846
lpg0346	0.771		*lpg1347*	0.674		*lpg2339*	0.873
lpg0347	0.611		*lpg1348*	0.860		*lpg2340*	0.874
lpg0348	0.844		*lpg1349*	0.858		*lpg2343*	0.868
lpg0349	0.865		*lpg1350*	0.853		*lpg2345*	0.867
lpg0352	0.758		*lpg1351*	0.852		*lpg2346*	0.866
lpg0353	0.873		*lpg1352*	0.897		*lpg2347*	0.863
lpg0354	0.887		*lpg1358*	0.865		*lpg2348*	0.863
lpg0355	0.848		*lpg1359*	0.863		*lpg2349*	0.850
lpg0356	0.900		*lpg1360*	0.835		*lpg2350*	0.858
lpg0357	0.911		*lpg1363*	0.866		*lpg2352*	0.868
lpg0359	0.873		*lpg1364*	0.849		*lpg2353*	0.765
lpg0361	0.916		*lpg1365*	0.752		*lpg2354*	0.858
lpg0362	0.905		*lpg1366*	0.857		*lpg2355*	0.859
lpg0363	0.850		*lpg1367*	0.859		*lpg2356*	0.874
lpg0365	0.876		*lpg1369*	0.858		*lpg2358*	0.249
lpg0366	0.860		*lpg1370*	0.659		*lpg2359*	0.849
lpg0369	0.877		*lpg1372*	0.854		*lpg2386*	0.857
lpg0370	0.822		*lpg1373*	0.854		*lpg2387*	0.873
lpg0371	0.854		*lpg1374*	0.851		*lpg2388*	0.886
lpg0372	0.851		*lpg1375*	0.856		*lpg2389*	0.888
lpg0374	0.823		*lpg1376*	0.828		*lpg2391*	0.891
lpg0376	0.884		*lpg1377*	0.681		*lpg2393*	0.850
lpg0377	0.857		*lpg1385*	0.843		*lpg2396*	0.882
lpg0380	0.806		*lpg1386*	0.846		*lpg2401*	0.883
lpg0382	0.858		*lpg1388*	0.848		*lpg2404*	0.877
lpg0383	0.851		*lpg1391*	0.625		*lpg2405*	0.697
lpg0384	0.867		*lpg1392*	0.845		*lpg2411*	0.834
lpg0385	0.847		*lpg1394*	0.862		*lpg2413*	0.844
lpg0386	0.855		*lpg1395*	0.830		*lpg2414*	0.761
lpg0387	0.820		*lpg1396*	0.610		*lpg2433*	0.878
lpg0388	0.863		*lpg1397*	0.804		*lpg2434*	0.873
lpg0391	0.855		*lpg1398*	0.865		*lpg2435*	0.866
lpg0392	0.868		*lpg1399*	0.847		*lpg2436*	0.876
lpg0393	0.860		*lpg1400*	0.771		*lpg2438*	0.876
lpg0394	0.867		*lpg1401*	0.735		*lpg2439*	0.877
lpg0395	0.801		*lpg1402*	0.868		*lpg2440*	0.888
lpg0396	0.860		*lpg1403*	0.876		*lpg2442*	0.863
lpg0399	0.542		*lpg1404*	0.870		*lpg2443*	0.867
lpg0400	0.863		*lpg1405*	0.863		*lpg2445*	0.864
lpg0404	0.862		*lpg1406*	0.854		*lpg2453*	0.844
lpg0405	0.850		*lpg1408*	0.858		*lpg2454*	0.851
lpg0406	0.755		*lpg1409*	0.863		*lpg2457*	0.714
lpg0407	0.841		*lpg1410*	0.858		*lpg2459*	0.863
lpg0408	0.845		*lpg1411*	0.850		*lpg2460*	0.868
lpg0409	0.854		*lpg1414*	0.862		*lpg2461*	0.869

lpg0410	0.841		*lpg1415*	0.737		*lpg2463*	0.811
lpg0411	0.857		*lpg1416*	0.811		*lpg2467*	0.884
lpg0413	0.849		*lpg1417*	0.852		*lpg2468*	0.863
lpg0414	0.849		*lpg1419*	0.859		*lpg2469*	0.864
lpg0415	0.771		*lpg1420*	0.838		*lpg2472*	0.880
lpg0418	0.867		*lpg1421*	0.866		*lpg2473*	0.862
lpg0419	0.857		*lpg1422*	0.766		*lpg2475*	0.889
lpg0421	0.858		*lpg1424*	0.850		*lpg2476*	0.748
lpg0422	0.866		*lpg1425*	0.863		*lpg2481*	0.896
lpg0423	0.834		*lpg1429*	0.826		*lpg2483*	0.825
lpg0424	0.849		*lpg1430*	0.845		*lpg2484*	0.867
lpg0425	0.865		*lpg1431*	0.769		*lpg2485*	0.893
lpg0426	0.625		*lpg1432*	0.848		*lpg2487*	0.830
lpg0428	0.858		*lpg1434*	0.830		*lpg2491*	0.841
lpg0432	0.863		*lpg1435*	0.831		*lpg2493*	0.855
lpg0433	0.850		*lpg1441*	0.840		*lpg2494*	0.870
lpg0439	0.859		*lpg1444*	0.841		*lpg2495*	0.881
lpg0440	0.706		*lpg1445*	0.846		*lpg2497*	0.875
lpg0442	0.821		*lpg1446*	0.851		*lpg2500*	0.888
lpg0443	0.847		*lpg1447*	0.847		*lpg2506*	0.908
lpg0444	0.843		*lpg1451*	0.849		*lpg2507*	0.898
lpg0445	0.854		*lpg1452*	0.858		*lpg2513*	0.885
lpg0446	0.870		*lpg1453*	0.847		*lpg2514*	0.876
lpg0447	0.844		*lpg1455*	0.763		*lpg2515*	0.859
lpg0448	0.847		*lpg1456*	0.844		*lpg2516*	0.878
lpg0449	0.758		*lpg1457*	0.876		*lpg2517*	0.695
lpg0450	0.865		*lpg1459*	0.846		*lpg2518*	0.864
lpg0452	0.822		*lpg1460*	0.847		*lpg2520*	0.827
lpg0453	0.827		*lpg1461*	0.855		*lpg2526*	0.863
lpg0454	0.771		*lpg1462*	0.837		*lpg2528*	0.877
lpg0455	0.812		*lpg1463*	0.858		*lpg2530*	0.862
lpg0456	0.813		*lpg1464*	0.845		*lpg2531*	0.855
lpg0457	0.817		*lpg1466*	0.846		*lpg2532*	0.861
lpg0458	0.832		*lpg1469*	0.849		*lpg2534*	0.736
lpg0459	0.797		*lpg1472*	0.867		*lpg2535*	0.856
lpg0460	0.879		*lpg1473*	0.877		*lpg2536*	0.856
lpg0461	0.875		*lpg1474*	0.875		*lpg2538*	0.890
lpg0462	0.860		*lpg1475*	0.853		*lpg2544*	0.890
lpg0463	0.788		*lpg1476*	0.786		*lpg2547*	0.850
lpg0464	0.737		*lpg1477*	0.872		*lpg2549*	0.836
lpg0468	0.850		*lpg1482*	0.842		*lpg2552*	0.849
lpg0469	0.823		*lpg1483*	0.860		*lpg2554*	0.818
lpg0471	0.735		*lpg1484*	0.852		*lpg2576*	0.873
lpg0473	0.651		*lpg1485*	0.842		*lpg2577*	0.890
lpg0474	0.728		*lpg1486*	0.853		*lpg2578*	0.753
lpg0475	0.581		*lpg1487*	0.829		*lpg2579*	0.862

lpg0476	0.654		*lpg1502*	0.856		*lpg2580*	0.866
lpg0477	0.862		*lpg1503*	0.857		*lpg2581*	0.870
lpg0478	0.607		*lpg1504*	0.854		*lpg2585*	0.863
lpg0479	0.802		*lpg1505*	0.596		*lpg2586*	0.863
lpg0481	0.833		*lpg1506*	0.841		*lpg2587*	0.878
lpg0482	0.859		*lpg1507*	0.843		*lpg2589*	0.865
lpg0483	0.866		*lpg1508*	0.853		*lpg2590*	0.859
lpg0485	0.747		*lpg1509*	0.848		*lpg2592*	0.840
lpg0491	0.865		*lpg1511*	0.851		*lpg2594*	0.835
lpg0493	0.845		*lpg1512*	0.864		*lpg2595*	0.846
lpg0497	0.856		*lpg1513*	0.844		*lpg2596*	0.866
lpg0498	0.888		*lpg1514*	0.840		*lpg2597*	0.885
lpg0499	0.858		*lpg1517*	0.846		*lpg2598*	0.834
lpg0500	0.885		*lpg1519*	0.879		*lpg2601*	0.829
lpg0506	0.900		*lpg1520*	0.865		*lpg2602*	0.861
lpg0507	0.873		*lpg1524*	0.863		*lpg2604*	0.867
lpg0510	0.859		*lpg1526*	0.835		*lpg2605*	0.862
lpg0511	0.809		*lpg1527*	0.879		*lpg2606*	0.837
lpg0512	0.669		*lpg1529*	0.888		*lpg2608*	0.835
lpg0513	0.901		*lpg1530*	0.882		*lpg2611*	0.850
lpg0525	0.865		*lpg1531*	0.881		*lpg2614*	0.755
lpg0528	0.836		*lpg1534*	0.882		*lpg2615*	0.841
lpg0529	0.827		*lpg1535*	0.714		*lpg2616*	0.858
lpg0530	0.862		*lpg1536*	0.867		*lpg2619*	0.858
lpg0531	0.828		*lpg1537*	0.867		*lpg2620*	0.908
lpg0532	0.884		*lpg1539*	0.850		*lpg2621*	0.832
lpg0533	0.864		*lpg1540*	0.843		*lpg2622*	0.882
lpg0534	0.860		*lpg1541*	0.887		*lpg2623*	0.840
lpg0535	0.856		*lpg1542*	0.883		*lpg2624*	0.832
lpg0536	0.859		*lpg1543*	0.880		*lpg2625*	0.899
lpg0539	0.838		*lpg1545*	0.849		*lpg2626*	0.831
lpg0540	0.883		*lpg1546*	0.844		*lpg2627*	0.846
lpg0541	0.838		*lpg1547*	0.849		*lpg2628*	0.769
lpg0542	0.483		*lpg1548*	0.837		*lpg2629*	0.790
lpg0547	0.849		*lpg1549*	0.861		*lpg2630*	0.870
lpg0548	0.740		*lpg1550*	0.842		*lpg2631*	0.871
lpg0551	0.846		*lpg1553*	0.845		*lpg2632*	0.764
lpg0552	0.727		*lpg1554*	0.873		*lpg2633*	0.842
lpg0556	0.753		*lpg1558*	0.841		*lpg2634*	0.811
lpg0557	0.827		*lpg1559*	0.844		*lpg2635*	0.829
lpg0558	0.780		*lpg1562*	0.857		*lpg2636*	0.561
lpg0559	0.843		*lpg1564*	0.852		*lpg2641*	0.863
lpg0560	0.834		*lpg1565*	0.850		*lpg2643*	0.869
lpg0561	0.859		*lpg1566*	0.849		*lpg2645*	0.874
lpg0562	0.754		*lpg1567*	0.853		*lpg2650*	0.075
lpg0563	0.672		*lpg1568*	0.864		*lpg2651*	0.678

lpg0564	0.871		*lpg1573*	0.837		*lpg2652*	0.761
lpg0565	0.815		*lpg1575*	0.847		*lpg2653*	0.757
lpg0566	0.758		*lpg1576*	0.862		*lpg2654*	0.877
lpg0568	0.868		*lpg1577*	0.840		*lpg2655*	0.866
lpg0577	0.854		*lpg1578*	0.664		*lpg2656*	0.864
lpg0580	0.853		*lpg1579*	0.847		*lpg2657*	0.877
lpg0581	0.833		*lpg1580*	0.878		*lpg2658*	0.772
lpg0583	0.843		*lpg1582*	0.846		*lpg2659*	0.861
lpg0584	0.820		*lpg1584*	0.854		*lpg2660*	0.858
lpg0585	0.798		*lpg1585*	0.790		*lpg2661*	0.856
lpg0586	0.756		*lpg1586*	0.864		*lpg2662*	0.786
lpg0587	0.734		*lpg1587*	0.751		*lpg2663*	0.782
lpg0588	0.840		*lpg1589*	0.836		*lpg2666*	0.865
lpg0591	0.560		*lpg1592*	0.808		*lpg2667*	0.834
lpg0592	0.789		*lpg1593*	0.858		*lpg2668*	0.864
lpg0593	0.854		*lpg1595*	0.859		*lpg2671*	0.861
lpg0594	0.616		*lpg1596*	0.865		*lpg2672*	0.862
lpg0595	0.860		*lpg1597*	0.867		*lpg2673*	0.859
lpg0596	0.862		*lpg1604*	0.847		*lpg2674*	0.761
lpg0598	0.866		*lpg1605*	0.808		*lpg2677*	0.868
lpg0599	0.876		*lpg1612*	0.853		*lpg2678*	0.855
lpg0600	0.864		*lpg1618*	0.863		*lpg2679*	0.846
lpg0601	0.882		*lpg1620*	0.860		*lpg2680*	0.870
lpg0602	0.816		*lpg1623*	0.866		*lpg2682*	0.828
lpg0603	0.885		*lpg1624*	0.854		*lpg2684*	0.872
lpg0604	0.858		*lpg1636*	0.887		*lpg2687*	0.833
lpg0605	0.844		*lpg1638*	0.877		*lpg2688*	0.838
lpg0606	0.793		*lpg1639*	0.874		*lpg2690*	0.866
lpg0607	0.856		*lpg1640*	0.830		*lpg2691*	0.866
lpg0608	0.851		*lpg1641*	0.887		*lpg2692*	0.846
lpg0611	0.858		*lpg1644*	0.800		*lpg2693*	0.846
lpg0612	0.864		*lpg1645*	0.857		*lpg2694*	0.854
lpg0614	0.722		*lpg1646*	0.849		*lpg2696*	0.818
lpg0616	0.880		*lpg1650*	0.881		*lpg2698*	0.895
lpg0618	0.838		*lpg1653*	0.846		*lpg2699*	0.751
lpg0622	0.846		*lpg1656*	0.860		*lpg2700*	0.864
lpg0623	0.834		*lpg1657*	0.845		*lpg2701*	0.843
lpg0624	0.814		*lpg1659*	0.856		*lpg2702*	0.836
lpg0626	0.851		*lpg1661*	0.854		*lpg2703*	0.859
lpg0627	0.822		*lpg1662*	0.861		*lpg2704*	0.871
lpg0629	0.842		*lpg1663*	0.746		*lpg2705*	0.816
lpg0630	0.843		*lpg1666*	0.870		*lpg2706*	0.802
lpg0631	0.814		*lpg1667*	0.855		*lpg2707*	0.677
lpg0633	0.816		*lpg1669*	0.870		*lpg2708*	0.785
lpg0634	0.855		*lpg1672*	0.794		*lpg2709*	0.830
lpg0640	0.839		*lpg1674*	0.837		*lpg2710*	0.863

lpg0641	0.872		*lpg1679*	0.797		*lpg2711*	0.820
lpg0643	0.875		*lpg1680*	0.837		*lpg2712*	0.525
lpg0650	0.520		*lpg1682*	0.846		*lpg2713*	0.803
lpg0651	0.855		*lpg1690*	0.863		*lpg2714*	0.861
lpg0652	0.865		*lpg1696*	0.880		*lpg2716*	0.794
lpg0654	0.824		*lpg1697*	0.727		*lpg2717*	0.776
lpg0656	0.878		*lpg1698*	0.680		*lpg2719*	0.859
lpg0657	0.862		*lpg1699*	0.804		*lpg2720*	0.859
lpg0658	0.889		*lpg1700*	0.723		*lpg2722*	0.851
lpg0659	0.883		*lpg1701*	0.863		*lpg2724*	0.840
lpg0660	0.879		*lpg1705*	0.861		*lpg2725*	0.766
lpg0662	0.887		*lpg1706*	0.852		*lpg2726*	0.845
lpg0663	0.877		*lpg1707*	0.858		*lpg2727*	0.858
lpg0664	0.857		*lpg1710*	0.782		*lpg2732*	0.807
lpg0665	0.816		*lpg1711*	0.794		*lpg2735*	0.831
lpg0667	0.874		*lpg1712*	0.851		*lpg2736*	0.858
lpg0670	0.818		*lpg1713*	0.851		*lpg2737*	0.869
lpg0672	0.847		*lpg1714*	0.858		*lpg2739*	0.871
lpg0673	0.506		*lpg1720*	0.861		*lpg2740*	0.747
lpg0674	0.878		*lpg1721*	0.852		*lpg2741*	0.666
lpg0677	0.616		*lpg1722*	0.862		*lpg2742*	0.804
lpg0678	0.848		*lpg1723*	0.862		*lpg2743*	0.861
lpg0679	0.879		*lpg1724*	0.838		*lpg2755*	0.843
lpg0680	0.871		*lpg1725*	0.838		*lpg2756*	0.766
lpg0685	0.852		*lpg1727*	0.858		*lpg2757*	0.854
lpg0686	0.871		*lpg1730*	0.852		*lpg2758*	0.864
lpg0687	0.393		*lpg1731*	0.850		*lpg2760*	0.839
lpg0688	0.869		*lpg1732*	0.859		*lpg2762*	0.856
lpg0689	0.815		*lpg1733*	0.854		*lpg2763*	0.838
lpg0692	0.881		*lpg1734*	0.857		*lpg2764*	0.830
lpg0697	0.896		*lpg1735*	0.708		*lpg2765*	0.810
lpg0698	0.864		*lpg1736*	0.858		*lpg2766*	0.858
lpg0699	0.813		*lpg1737*	0.862		*lpg2769*	0.824
lpg0700	0.794		*lpg1743*	0.698		*lpg2772*	0.870
lpg0701	0.861		*lpg1744*	0.842		*lpg2773*	0.869
lpg0704	0.820		*lpg1746*	0.854		*lpg2774*	0.840
lpg0712	0.881		*lpg1747*	0.843		*lpg2777*	0.868
lpg0716	0.831		*lpg1748*	0.821		*lpg2778*	0.873
lpg0719	0.866		*lpg1749*	0.827		*lpg2779*	0.522
lpg0720	0.872		*lpg1750*	0.876		*lpg2780*	0.838
lpg0721	0.865		*lpg1751*	0.851		*lpg2781*	0.844
lpg0722	0.441		*lpg1752*	0.747		*lpg2782*	0.860
lpg0723	0.849		*lpg1753*	0.869		*lpg2783*	0.860
lpg0724	0.670		*lpg1754*	0.840		*lpg2785*	0.851
lpg0725	0.863		*lpg1755*	0.853		*lpg2786*	0.868
lpg0726	0.803		*lpg1756*	0.732		*lpg2787*	0.809

lpg0729	0.802		*lpg1757*	0.817		*lpg2788*	0.832
lpg0730	0.809		*lpg1758*	0.787		*lpg2789*	0.803
lpg0732	0.753		*lpg1759*	0.818		*lpg2791*	0.831
lpg0734	0.852		*lpg1761*	0.765		*lpg2792*	0.855
lpg0737	0.842		*lpg1762*	0.820		*lpg2794*	0.841
lpg0738	0.854		*lpg1763*	0.813		*lpg2795*	0.858
lpg0739	0.846		*lpg1764*	0.802		*lpg2796*	0.867
lpg0740	0.809		*lpg1765*	0.789		*lpg2797*	0.839
lpg0741	0.800		*lpg1766*	0.814		*lpg2798*	0.858
lpg0742	0.841		*lpg1767*	0.790		*lpg2799*	0.864
lpg0745	0.833		*lpg1768*	0.709		*lpg2805*	0.861
lpg0747	0.842		*lpg1770*	0.472		*lpg2806*	0.860
lpg0748	0.844		*lpg1771*	0.786		*lpg2808*	0.861
lpg0749	0.728		*lpg1772*	0.770		*lpg2809*	0.868
lpg0752	0.878		*lpg1778*	0.805		*lpg2812*	0.861
lpg0753	0.845		*lpg1779*	0.551		*lpg2814*	0.863
lpg0754	0.849		*lpg1782*	0.827		*lpg2817*	0.855
lpg0755	0.420		*lpg1785*	0.822		*lpg2818*	0.846
lpg0759	0.871		*lpg1788*	0.766		*lpg2819*	0.853
lpg0760	0.792		*lpg1789*	0.828		*lpg2822*	0.858
lpg0781	0.414		*lpg1791*	0.822		*lpg2823*	0.850
lpg0785	0.857		*lpg1792*	0.821		*lpg2824*	0.863
lpg0786	0.908		*lpg1793*	0.777		*lpg2825*	0.000
lpg0791	0.784		*lpg1798*	0.885		*lpg2827*	0.870
lpg0800	0.786		*lpg1800*	0.449		*lpg2833*	0.756
lpg0801	0.782		*lpg1803*	0.771		*lpg2835*	0.863
lpg0802	0.786		*lpg1804*	0.855		*lpg2836*	0.876
lpg0803	0.800		*lpg1805*	0.906		*lpg2837*	0.851
lpg0804	0.736		*lpg1806*	0.825		*lpg2838*	0.846
lpg0805	0.757		*lpg1807*	0.893		*lpg2842*	0.874
lpg0808	0.741		*lpg1808*	0.792		*lpg2843*	0.872
lpg0810	0.772		*lpg1809*	0.531		*lpg2847*	0.851
lpg0811	0.807		*lpg1810*	0.682		*lpg2848*	0.856
lpg0812	0.799		*lpg1811*	0.685		*lpg2851*	0.869
lpg0815	0.701		*lpg1812*	0.758		*lpg2853*	0.855
lpg0816	0.794		*lpg1813*	0.850		*lpg2855*	0.867
lpg0817	0.759		*lpg1814*	0.660		*lpg2858*	0.855
lpg0818	0.832		*lpg1815*	0.881		*lpg2859*	0.853
lpg0821	0.809		*lpg1816*	0.893		*lpg2860*	0.849
lpg0822	0.832		*lpg1821*	0.880		*lpg2861*	0.863
lpg0823	0.649		*lpg1823*	0.870		*lpg2864*	0.872
lpg0824	0.792		*lpg1824*	0.869		*lpg2865*	0.827
lpg0825	0.786		*lpg1825*	0.895		*lpg2867*	0.862
lpg0826	0.818		*lpg1826*	0.557		*lpg2868*	0.864
lpg0829	0.801		*lpg1830*	0.909		*lpg2869*	0.882
lpg0833	0.788		*lpg1831*	0.889		*lpg2872*	0.724

lpg0834	0.807		*lpg1832*	0.857		*lpg2873*	0.858
lpg0835	0.783		*lpg1833*	0.847		*lpg2874*	0.856
lpg0836	0.774		*lpg1834*	0.861		*lpg2875*	0.863
lpg0837	0.768		*lpg1835*	0.840		*lpg2878*	0.855
lpg0838	0.744		*lpg1836*	0.865		*lpg2879*	0.864
lpg0839	0.781		*lpg1837*	0.879		*lpg2880*	0.859
lpg0840	0.799		*lpg1838*	0.861		*lpg2881*	0.848
lpg0841	0.786		*lpg1839*	0.884		*lpg2882*	0.884
lpg0842	0.820		*lpg1840*	0.840		*lpg2883*	0.854
lpg0843	0.452		*lpg1841*	0.867		*lpg2884*	0.854
lpg0845	0.792		*lpg1842*	0.872		*lpg2885*	0.866
lpg0846	0.739		*lpg1843*	0.778		*lpg2886*	0.865
lpg0847	0.809		*lpg1844*	0.782		*lpg2887*	0.862
lpg0848	0.813		*lpg1845*	0.804		*lpg2890*	0.856
lpg0849	0.765		*lpg1846*	0.849		*lpg2891*	0.867
lpg0851	0.817		*lpg1847*	0.856		*lpg2894*	0.868
lpg0852	0.772		*lpg1849*	0.817		*lpg2897*	0.877
lpg0853	0.785		*lpg1850*	0.836		*lpg2898*	0.868
lpg0854	0.699		*lpg1851*	0.854		*lpg2899*	0.836
lpg0856	0.830		*lpg1854*	0.840		*lpg2900*	0.870
lpg0858	0.794		*lpg1855*	0.918		*lpg2901*	0.841
lpg0859	0.099		*lpg1858*	0.233		*lpg2902*	0.863
lpg0860	0.731		*lpg1859*	0.876		*lpg2903*	0.851
lpg0862	0.819		*lpg1860*	0.882		*lpg2904*	0.870
lpg0865	0.799		*lpg1861*	0.872		*lpg2905*	0.879
lpg0866	0.821		*lpg1869*	0.918		*lpg2907*	0.846
lpg0867	0.841		*lpg1870*	0.876		*lpg2908*	0.883
lpg0869	0.834		*lpg1871*	0.921		*lpg2916*	0.869
lpg0870	0.844		*lpg1873*	0.879		*lpg2924*	0.868
lpg0871	0.725		*lpg1874*	0.856		*lpg2925*	0.871
lpg0872	0.819		*lpg1882*	0.907		*lpg2926*	0.779
lpg0873	0.801		*lpg1883*	0.825		*lpg2927*	0.869
lpg0874	0.824		*lpg1887*	0.862		*lpg2928*	0.860
lpg0875	0.502		*lpg1888*	0.880		*lpg2929*	0.827
lpg0877	0.829		*lpg1889*	0.882		*lpg2930*	0.864
lpg0878	0.600		*lpg1891*	0.780		*lpg2931*	0.815
lpg0879	0.827		*lpg1892*	0.862		*lpg2933*	0.877
lpg0880	0.822		*lpg1893*	0.879		*lpg2934*	0.867
lpg0882	0.804		*lpg1894*	0.869		*lpg2935*	0.814
lpg0885	0.813		*lpg1895*	0.793		*lpg2937*	0.867
lpg0886	0.824		*lpg1896*	0.859		*lpg2951*	0.868
lpg0887	0.803		*lpg1904*	0.853		*lpg2953*	0.861
lpg0888	0.806		*lpg1905*	0.852		*lpg2955*	0.735
lpg0889	0.674		*lpg1906*	0.878		*lpg2956*	0.846
lpg0890	0.796		*lpg1908*	0.876		*lpg2957*	0.840
lpg0891	0.867		*lpg1909*	0.882		*lpg2960*	0.850

lpg0892	0.826		*lpg1910*	0.883		*lpg2962*	0.864
lpg0895	0.716		*lpg1911*	0.884		*lpg2963*	0.846
lpg0896	0.811		*lpg1913*	0.887		*lpg2964*	0.863
lpg0897	0.792		*lpg1915*	0.882		*lpg2965*	0.850
lpg0899	0.821		*lpg1916*	0.870		*lpg2966*	0.689
lpg0900	0.832		*lpg1917*	0.911		*lpg2967*	0.843
lpg0901	0.805		*lpg1918*	0.882		*lpg2968*	0.866
lpg0902	0.817		*lpg1919*	0.877		*lpg2969*	0.852
lpg0904	0.801		*lpg1920*	0.785		*lpg2970*	0.855
lpg0905	0.819		*lpg1921*	0.878		*lpg2971*	0.868
lpg0906	0.783		*lpg1924*	0.892		*lpg2972*	0.870
lpg0907	0.807		*lpg1927*	0.796		*lpg2974*	0.867
lpg0908	0.785		*lpg1942*	0.860		*lpg2975*	0.869
lpg0909	0.798		*lpg1943*	0.562		*lpg2976*	0.860
lpg0910	0.795		*lpg1944*	0.869		*lpg2982*	0.850
lpg0911	0.786		*lpg1945*	0.864		*lpg2983*	0.842
lpg0915	0.714		*lpg1949*	0.864		*lpg2985*	0.730
lpg0917	0.807		*lpg1993*	0.849		*lpg2986*	0.708
lpg0918	0.816		*lpg1994*	0.867		*lpg2987*	0.050
lpg0919	0.818		*lpg1999*	0.765		*lpg2990*	0.399
lpg0920	0.771		*lpg2000*	0.866		*lpg2991*	0.829
lpg0921	0.860		*lpg2001*	0.868		*lpg2993*	0.847
lpg0922	0.840		*lpg2002*	0.663		*lpg2994*	0.741
lpg0923	0.842		*lpg2004*	0.869		*lpg2995*	0.810
lpg0924	0.841		*lpg2007*	0.845		*lpg2996*	0.852
lpg0925	0.853		*lpg2008*	0.842		*lpg2997*	0.856
lpg0926	0.849		*lpg2009*	0.857		*lpg2998*	0.868
lpg0927	0.837		*lpg2010*	0.843		*lpg2999*	0.869
lpg0928	0.837		*lpg2011*	0.864		*lpg3002*	0.859
lpg0929	0.827		*lpg2012*	0.807		*lpg3005*	0.516

References

1. Cazalet C, Rusniok C, Bruggemann H, Zidane N, Magnier A, Ma L, Tichit M, Jarraud S, Bouchier C, Vandenesch F, Kunst F, Etienne J, Glaser P, Buchrieser C. 2004. Evidence in the Legionella pneumophila genome for exploitation of host cell functions and high genome plasticity. Nature Genetics 36:1165-1173.

2. Reuter S, Harrison TG, Koeser CU, Ellington MJ, Smith GP, Parkhill J, Peacock SJ, Bentley SD, Toeroek ME. 2013. A pilot study of rapid whole-genome sequencing for the investigation of a Legionella outbreak. Bmj Open 3.

3. Gomez-Valero L, Rusniok C, Jarraud S, Vacherie B, Rouy Z, Barbe V, Medigue C, Etienne J, Buchrieser C. 2011. Extensive recombination events and horizontal gene transfer shaped the Legionella pneumophila genomes. Bmc Genomics 12.

4. Underwood AP, Jones G, Mentasti M, Fry NK, Harrison TG. 2013. Comparison of the Legionella pneumophila population structure as determined by sequence-based typing and whole genome sequencing. Bmc Microbiology 13.

5. D'Auria G, Jimenez-Hernandez, N., Peris-Bondia, F., Moya, A., Latorre, A. 2010. Legionella pneumophila pangenome reveals strain-specific virulence factors. Bmc Genomics 11.

6. Chien MC, Morozova I, Shi SD, Sheng HT, Chen J, Gomez SM, Asamani G, Hill K, Nuara J, Feder M, Rineer J, Greenberg JJ, Steshenko V, Park SH, Zhao BH, Teplitskaya E, Edwards JR, Pampou S, Georghiou A, Chou IC, Iannuccilli W, Ulz ME, Kim DH, Geringer-Sameth A, Goldsberry C, Morozov P, Fischer SG, Segal G, Qu XY, Rzhetsky A, Zhang PS, Cayanis E, De Jong PJ, Ju JY, Kalachikov S, Shuman HA, Russo JJ. 2004. The genomic sequence of the accidental pathogen Legionella pneumophila. Science 305:1966-1968.

7. Gloeckner G, Albert-Weissenberger C, Weinmann E, Jacobi S, Schunder E, Steinert M, Hacker J, Heuner K. 2008. Identification and characterization of a new conjugation/type IVA secretion system (trb/tra) of Legionella pneumophila Corby localized on two mobile genomic islands. International Journal of Medical Microbiology 298:411-428.

8. Ma J, He, Y., Hu, B., Luo Z-Q. 2013. Genome sequence of an environmental isolate of the bacterial pathogen Legionella pneumophila. Genome Announcements 1.

9. Amaro F, Gilbert JA, Owens S, Trimble W, Shuman HA. 2012. Whole-genome sequence of the human pathogen Legionella pneumophila serogroup 12 strain 570-CO-H. Journal of Bacteriology 194:1613-1614.

10. Khan MA, Knox N, Prashar A, Alexander D, Abdel-Nour M, Duncan C, Tang P, Amatullah H, Dos Santos CC, Tijet N, Low DE, Pourcel C, Van Domselaar G, Terebiznik M, Ensminger AW, Guyard C. 2013. Comparative Genomics Reveal That Host-Innate Immune Responses Influence the Clinical Prevalence of Legionella pneumophila Serogroups. Plos One 8.
